# Assigning the absolute configuration of single aliphatic molecules by visual inspection

**DOI:** 10.1038/s41467-018-04843-z

**Published:** 2018-06-20

**Authors:** Daniel Ebeling, Marina Šekutor, Marvin Stiefermann, Jalmar Tschakert, Jeremy E. P. Dahl, Robert M. K. Carlson, André Schirmeisen, Peter R. Schreiner

**Affiliations:** 1 Institute of Applied Physics, Justus-Liebig University, Heinrich-Buff-Ring 16, 35392 Giessen, Germany; 2 Institute of Organic Chemistry, Justus-Liebig University, Heinrich-Buff-Ring 17, 35392 Giessen, Germany; 3 Stanford Institute for Materials and Energy Sciences, Stanford, CA 94305 USA

## Abstract

Deciphering absolute configuration of a single molecule by direct visual inspection is the next step in compound identification, with far-reaching implications for medicinal chemistry, pharmacology, and natural product synthesis. We demonstrate the feasibility of this approach utilizing low temperature atomic force microscopy (AFM) with a CO-functionalized tip to determine the absolute configuration and orientation of a single, adsorbed [123]tetramantane molecule, the smallest chiral diamondoid. We differentiate between single enantiomers on Cu(111) by direct visual inspection, and furthermore identify molecular dimers and molecular clusters. The experimental results are confirmed by a computational study that allowed quantification of the corresponding intermolecular interactions. The unique toolset of absolute configuration determination combined with AFM tip manipulation opens a route for studying molecular nucleation, including chirality-driven assembly or reaction mechanisms.

## Abstract (web summary)

Deciphering absolute configuration of individual molecules directly by visual inspection remains a highly attractive goal. Here, the authors determine the absolute configuration and orientation of a single [123]tetramantane molecule adsorbed on Cu(111) using low temperature atomic force microscopy with a CO-functionalized tip.

## Introduction

Life as we know it would not exist without chirality. Homochirality of biological building blocks, e.g., L-amino acids and D-sugars, is critical for molecular recognition and has been fundamental for the evolution of life on Earth^1^. The mirror images (enantiomers or right-handed and left-handed forms) of the same compound can interact with living organisms in a completely different manner. As a consequence, for individual enantiomers we often observe differences in taste, smell, and in the case of pharmaceuticals, adverse medical effects^2^. Louis Pasteur was the first to demonstrate molecular chirality by separating right-handed and left-handed forms of tartaric acid crystals using a light microscope and then analyzing their optical activity^3^. At first glance, preferential crystallization seems to be a facile method for obtaining enantiomerically pure material; however, compounds capable of spontaneous symmetry breaking upon crystallization are quite rare and this phenomenon is not well understood^4, 5^. Formation of single-enantiomer crystals occurs so rarely because the Gibbs free energy (∆*G*) of racemate crystallization is almost always negative, thereby favouring the formation of a racemic crystal containing both enantiomers^6^. Additionally, the chirality of crystals does not translate directly into the absolute stereochemistry of a molecule because the absolute configuration can be assigned only after fitting molecular orientation to the crystal polar axis^7^. Since the time of Pasteur’s experiment, various analytical techniques have been perfected to help determine the absolute configurations of compounds, e.g., measurement of optical rotation, circular dichroism, X-ray analysis, NMR spectroscopic methods, etc.^8, 9^, but to assign absolute configuration of individual molecules directly by visual inspection remains a highly attractive goal^10^. This would constitute a major advance for chemistry.

Previously, assignment of enantiomers without chromophores, e.g., chiral alkanes^11, 12^ and many natural products, which display very small optical rotations, was particularly difficult and led to high uncertainties and well-documented misassignments^13^. Although isolation and structure elucidation of natural products has progressed dramatically^14^, their identification by direct observation of single molecules would eliminate some persistent problems connected with natural product isolation and/or total synthesis, e.g., limited compound availability and a necessity for further functionalization requiring additional synthetic steps. Taking into account the present need for such a direct method, we decided to use helically chiral [123]tetramantane^15^ as a model system for determining absolute configuration by means of visual inspection of single molecules.

**Fig. 1:**
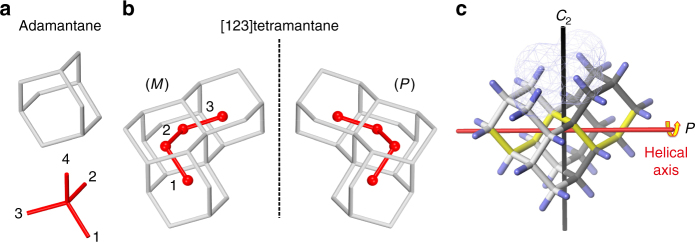
Structures of adamantane and enantiomers of [123]tetramantane. **a** Depiction of adamantane. **b** (*M*)-enantiomer and (*P*)-enantiomer of [123]tetramantane with hydrogen atoms omitted for clarity (numbers 1–4 are used to indicate the directions in Cartesian space of the diamond lattice and four red spheres in molecules indicate the centers of four corresponding adamantane cages). **c** The (*P*)-enantiomer illustrating the helical core (bonds marked in yellow) and close contacts between the hydrogens inside the molecular groove (transparent net area)

[123]Tetramantane, a natural product that can be isolated from petroleum^16^, is a chiral alkane belonging to the class of molecules known as diamondoids (Fig. 1)^17, 18^. Assignment of its enantiomers is particularly challenging using the established methods described above^15^ because it requires multiple tedious steps including: (1) HPLC separations using a chiral stationary phase, (2) crystal growth, (3) chemical functionalization, and (4) use of a variety of analytical and theoretical techniques to unambiguously assign the structures. To underscore the challenge of assigning the absolute configuration of some molecules without classic chromophores, it is noteworthy that the [*α*]_D_ value of [123]tetramantane enantiomers is only 34°. Therefore, there are no interpretable absorptions in the circular dichroism (CD) spectrum and the structure could only be reliably assigned using a match of computed and measured vibrational circular dichroism spectra^15^. Owing to these difficulties, we decided to approach the problem differently, by using a single molecule perspective.

With methods such as scanning tunneling microscopy (STM) or atomic force microscopy (AFM) it is possible to study chirality at the molecular level^19–21^. In particular, the reduction of the degrees of freedom, which results from analyzing adsorbed 2D molecular layers or small 2D clusters can facilitate differentiation between enantiomers. Following this strategy, along with choosing a substrate where surface reconstruction can take place, it is sometimes possible to identify the chirality of molecular dimers even without submolecular resolution^21^. However, to assign the absolute configuration at the level of individual molecules submolecular resolution is essential. For example, Ernst et al. recently demonstrated the concept of stereochemical assignment for helical molecules on a metal surface using the submolecular resolution capabilities of standard STM^10^. Furthermore, high-resolution AFM was also used to identify products of on-surface chirality transfer reactions from helicene substrates by determining the handedness of mostly planar single molecules^22^. In these two exemplary cases, however, the unique structure of the studied types of helical molecules facilitates their identification. Additionally, since such helicenes are non-natural aromatic hydrocarbons that typically have large optical rotations and intense CD spectra, their assignment by conventional means is straightforward. Extending the assignment of absolute configuration by direct visual inspection to single *sp*^3^ systems would therefore represent a significant advance.

For small bulky molecules it is difficult and unreliable to assign their configuration by using standard STM measurements (i.e., without tip functionalization) and only a rough estimation of their orientation is feasible, as demonstrated for single tetramantane molecules on a Au(111) surface^23, 24^. Hence, in order to study such types of molecules a technique with higher lateral resolution is needed, which can, e.g., be accomplished by functionalizing the STM/AFM tip. This so-called “bond imaging” technique has previously been introduced by Gross et al. who demonstrated that AFM resolution can be significantly enhanced by functionalizing the tip with a single CO molecule^25^. Similarly, proper tip functionalization can provide enhanced contrast in specific STM techniques^26–28^. In particular, for the study of aromatic compounds on surfaces these methods have been successfully applied to identify chemical structures, length, and order of intramolecular and intermolecular bonds, molecular assembly mechanisms, on-surface reaction pathways, and even chirality-related phenomena^22, 25, 29–38^.

Here we applied this technique to differentiate between single enantiomers of [123]tetramantane on a Cu(111) surface and determine their absolute configuration. This is the first time single enantiomers of naturally occurring bulky molecules have been distinguished solely by direct visual inspection. In addition, we studied the self-assembly of chiral molecules on metal surfaces and imaged [123]tetramantane dimers and small clusters which may act as centers for crystal nucleation.

## Results and Discussion

### STM/AFM imaging

**Fig. 2:**
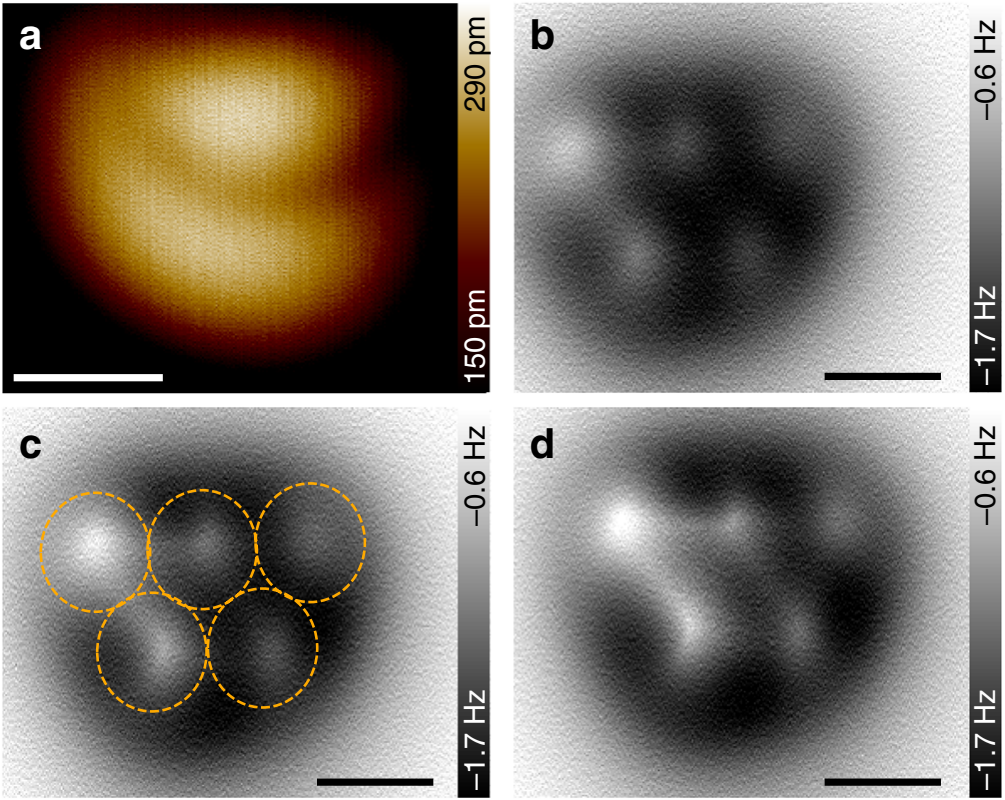
Single [123]tetramantane molecules on a Cu(111) surface. **a** Images obtained in STM scan mode. **b**–**d** Images obtained by constant height AFM scans. Parameters: **a** *U* = 200 mV, *I* = 10 pA, **b**–**d** *U* = −0.5 mV, Δ*z* = 170 pm (**b**), 160 pm (**c**), 150 pm (**d**), respectively. Scale bars: **a** 0.5 nm, **b**–**c** 0.3 nm

In order to investigate single [123]tetramantanes, the racemic mixture was sublimed onto a cold Cu(111) surface (below ≈15 K) through the opened temperature shields of the low-temperature STM/AFM instrument. At higher temperatures tetramantane tends to form islands^23, 36^, which are caused by a low diffusion barrier paired with strong intermolecular London dispersion (LD) interactions^36^. Figure 2 shows STM (a) and AFM (b–d) scans of single [123]tetramantanes that were measured at 5 K with a CO functionalized AFM tip^25^. The appearance of the molecules in two different imaging modes is rather different. In the STM mode the molecules appear as cloud-like structures that exhibit sub-molecular features (Fig. 2a). Since the STM contrast depends strongly on the local density of states, these features in general do not allow for direct identification of molecular structures. The AFM contrast, however, is mainly determined by short-range repulsive interactions, i.e., Pauli exchange repulsion and electrostatic interactions^25, 39^, which are known to produce a more detailed view of the chemical structure of adsorbed molecular species.

For 3D objects such as bulky cage molecules, however, direct identification of their structures and orientations on the surface is not straightforward^30, 38, 40–43^, although some recent advances in characterizing aliphatic moieties have been made^36, 43^. The CO molecule at the tip, which facilitates the submolecular contrast, is flexible and provokes distortions in the image contrast^25, 30, 41^. This flexibility of the CO tip leads to a force-dependent tilting that can impede the identification of 3D objects during constant height scanning. Therefore, the formation of the image contrast has to be addressed thoroughly at different imaging heights in order to resolve the atomic structure and determine the precise orientation of the molecules. Recently, we were able to image [121]tetramantanes with atomic resolution and determine their precise orientation on the surface^36^, so this method can be applied in a similar way to the [123]tetramantanes studied here.

In Fig. 2b-d three AFM images of the same molecule are depicted, which were determined at three different heights above the Cu substrate. All images were obtained in the constant height mode and the Δ*z*-offset is given with regard to the tip height over the Cu surface in tunneling feedback (*U* = 200 mV, *I* = 10 pA). Displayed is the frequency shift of the oscillating tuning fork sensor vs. its *x*/*y*-position. While repulsive tip–sample interactions lead to positive frequency shifts (bright colors), attractive interactions lead to negative frequency shifts (dark colors). The observed image contrast changes significantly when approaching the CO-tip to the sample surface. Initially, at distances relatively far away from the molecule, a dark region appears that is due to attractive LD interactions (not shown). Closer to the surface (Fig. 2b, c), the images reveal five bright spots, whose arrangement bears similarity to the Olympic rings (as indicated by dashed orange circles in c). When further approaching the surface artificial lines appear between the bright spots that are caused by the previously mentioned flexibility of the CO molecule (Fig. 2d). For closer tip–sample distances these effects get more severe and make the images non-analyzable.

### Determining the absolute configuration

**Fig. 3:**
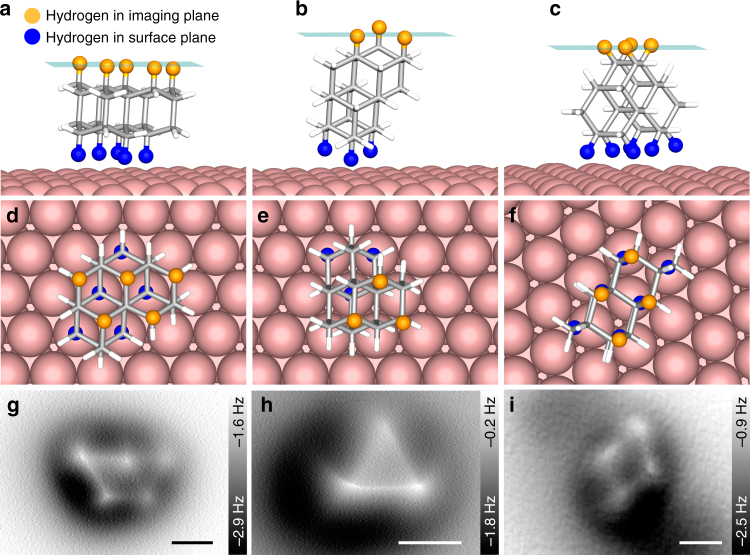
Three different orientations of (*M*)-[123]tetramantane. In the first two rows side (**a**–**c**) and top views (**d**–**f**) of the corresponding computed orientations are depicted. The planes in the first row indicate the imaging plane of the corresponding AFM scans (shown in the third row, **g**–**i**). Hydrogen atoms in the imaging and surface planes are marked in orange and blue, respectively. Each orientation results in a characteristic pattern of hydrogen atoms within the imaging plane, which resemble the shape of the Olympic rings (**d**, **g**), a triangle (**e**, **h**), and a rhombus (**f**, **i**). Scale bars: 0.3 nm

In the following we demonstrate that the observed Olympic ring pattern of five bright spots allows us to determine the precise orientation of the individual [123]tetramantane molecules and their absolute configuration. Fig. 3 shows three different orientations of [123]tetramantane that have been observed on the Cu(111) surface (left (a, d, g), middle (b, e, h), and right column (c, f, i)). The first two rows depict side and top views of the corresponding computed orientations (for details about the used GFN-xTB^44^ computational method vide infra). To each of the side views a semi-transparent plane was added, indicating the imaging planes of the corresponding AFM experiment. AFM images for each of the observed molecular orientations are depicted in the third row. Hydrogen atoms facing the Cu(111) surface are marked in blue, while hydrogen atoms within the imaging plane are marked in orange. All other hydrogen atoms are shown as white sticks for clarity. Each orientation leads to a characteristic pattern of hydrogen atoms within the imaging plane, which resemble the shape of the Olympic rings (d, g), a triangle (e, h), and a rhombus (f, i).

The majority of imaged molecules is found in the Olympic ring pattern (a, d, g) and the other two orientations are rarely observed. These orientations may be found after accidentally manipulating the molecules with the tip since this can happen during STM measurement with high tunneling setpoint or if the tip approaches the surface too closely during AFM constant height scanning. This observation is in agreement with our computational results, since the computed Gibbs energies at 15 K show that the orientation in the left column (Olympic ring pattern) is by 11.1 and 10.2 kcal mol^–1^ more favourable than the orientations in the middle and right columns, respectively (see Supplementary Table 7). In general, the adsorption of (*M*)-[123]tetramantane on a Cu(111) surface was found to be a favourable process, e.g., at 15 K the orientation in the left column is stabilized by –30.3 kcal mol^–1^ upon interaction of the isolated tetramantane molecule with the modelled copper slab.

In the following we will focus on identifying the absolute configuration of two [123]tetramantane enantiomers by visual inspection. Since the majority of the molecules are found with the Olympic ring pattern facing upwards (left column), we will describe the identification process using this orientation as an example.

**Fig. 4:**
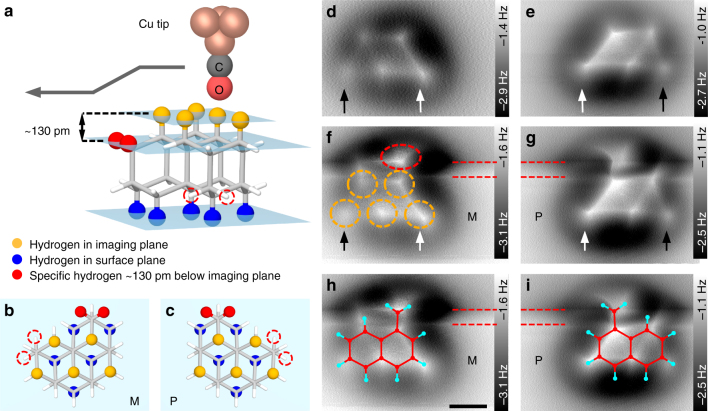
Adsorption structure of [123]tetramantanes. **a** Side view sketch of (*M*)-[123]tetramantane with the Olympic ring pattern of hydrogen atoms facing upwards (see five orange hydrogens). The absolute configuration can be determined by locating two specific hydrogen atoms (red atoms) ≈130 pm below the imaging plane. **b**, **c** Top view sketches of (*M*)-[123]tetramantane and (*P*)-[123]tetramantane showing the arrangement of Olympic ring patterns in the imaging and surface planes (orange and blue atoms) and the specific hydrogen atoms (red atoms and red dashed circles). **d**, **e** Constant height AFM scans of two different [123]tetramantanes. White and black arrows indicate the brighter (higher) and darker (lower) sides of the molecules, respectively. **f**, **g** Same molecules imaged with custom height profile, i.e., in the region between the two dashed red lines the imaging height was linearly decreased by ≈130 pm. Both scans were started in the bottom of the images. Both images reveal a bright halo (red dashed oval in **f**), which is either located on the right or the left side of the Olympic ring pattern (dashed orange circles). **h**, **i** Corresponding AFM scans with overlaid molecular structures of (*M*)-[123]tetramantane and (*P*)-[123]tetramantane. For clarity, only the plane containing the two specific hydrogens is plotted. Please note that the specific hydrogens are either connected to the C–12 or C–22 sites (according to IUPAC designated carbon numbers for [123]tetramantane). Scale bar: 0.3 nm

Fig. 4a-c shows side view and two top view sketches of the (*M*)-enantiomer and the (*P*)-enantiomer. In b and c two specific hydrogen atoms that are located ≈130 pm below the imaging plane are marked in red color, respectively. While these two specific hydrogens are located at the right side above the Olympic ring pattern for the (*M*)-type molecule, they are located at the left side for the (*P*)-type molecule (see top views b and c). Hence, for an unambiguous identification of the absolute configurations the positions of two specific hydrogen atoms, which reside ≈130 pm below the imaging plane, have to be determined. Figure 4d and e shows two constant height AFM scans of two different [123]tetramantanes. These scans reveal that the molecules are slightly tilted with respect to the surface plane, i.e., one side of the Olympic ring pattern appears slightly brighter than the other, as indicated by the white and dark arrows. To identify the absolute configuration of the molecules by imaging the specific hydrogens, we varied the height of the imaging plane during scanning as indicated in the sketch in Fig. 4a. The resulting AFM images are presented in Fig. 4f and g. Both scans were started at the bottom of the image and scanned in upward direction. After the tip has passed the Olympic ring pattern, the Δ*z*-offset was gradually reduced by ≈130 pm. The two red dashed lines indicate the image region where the offset was reduced. Apparently, both images reveal characteristic features (see red dashed oval in f) at the positions where the two specific hydrogen atoms are expected, i.e., at the right and left sides above the Olympic ring patterns. Herewith, we can unambiguously identify the (*M*)-enantiomer in Fig. 4f and the (*P*)-enantiomer in Fig. 4g (see also molecular overlays in Fig. 4h and i).

After performing this identification procedure, it is possible to distinguish between the two enantiomers by standard constant height AFM scans (i.e., without custom height profile). Since the molecules are not perfectly parallel to the surface plane, the brighter side of the Olympic ring pattern indicates the absolute configuration (see black and white arrows in Fig. 4d–g). Presumably the observed tilting of the molecules is caused by their *C*_2_-symmetry. The five hydrogen atoms within the Olympic ring pattern in the surface plane (see blue hydrogens in Fig. 4a–c) are rotated by 60° with respect to the Olympic ring pattern in the imaging plane (orange hydrogens). Two specific hydrogens (red atoms), which are indicators of handedness, also have corresponding counterparts close to the surface (red dashed circles in Fig. 4a–c). For the (*M*)-enantiomers and (*P*)-enantiomers, these hydrogens are located at opposite sides (cf. Fig. 4b and c). Interactions between these hydrogens and the Cu(111) surface are believed to pull down the molecules at the corresponding edges, provoking the observed tilting (see Supplementary Fig. 1 for more details).

### Computation of on-surface structures

To further confirm such tilting of [123]tetramantane on Cu(111) and to provide an estimate of the tilting angle, we performed semiempirical computations using the GFN-xTB approach^44^. This modern method allows for computation of large systems with the advantage of also taking non-covalent interactions into account. Since LD interactions play a vital role in the behavior of tetramantane molecules^36^, GFN-xTB that includes the well-established D3 dispersion correction was a method of choice for our system. As a sufficient representation of the Cu(111) surface we took a copper slab consisting of 216 Cu atoms, with dimensions of 18 × 18 × 5 Å. After introducing the corresponding orientation of tetramantane on the Cu slab and freezing the copper lattice, geometry optimizations and frequency analyses were performed. The obtained geometries along with their coordinates and corresponding energies are given in the Supplementary Information and the Supplementary Data (Supplementary Fig. 1, Supplementary Table 7, and Supplementary Data Set 3). Herewith, we successfully confirm that (*M*) and (*P*)-type molecules are tilted in opposite directions and we found tilting angles on the order of 4–5° with regard to the *x*/*y* plane.

Note that the shown molecular orientations correspond to local minima on the potential energy surface, i.e., other orientations with similar adsorption energies coexist. However, from our AFM experiments we can clearly infer the orientation of the Olympic ring pattern with regard to the Cu(111) lattice (see Supplementary Fig. 2). Our AFM images reveal that the Olympic ring pattern aligns to the crystallographic [1–10] direction (and equivalent [–101] and [01–1] directions), hence [123]tetramantanes are snapping to the Cu(111) lattice. We also used the tip as a manipulation tool in order to rotate the molecules. The manipulations result in rotations that correspond to integer multiples of 60°. Furthermore, the tilting with regard to the *x*/*y* plane was preserved after rotational manipulation, which supports the rationale that the observed tilting is not caused by a sometimes observed slight asymmetry of the CO-tip. Hence, from a number of computed orientations that correspond to different local minima we chose those that are most comparable to our experimental results.

### Imaging of dimers and small clusters

**Fig. 5:**
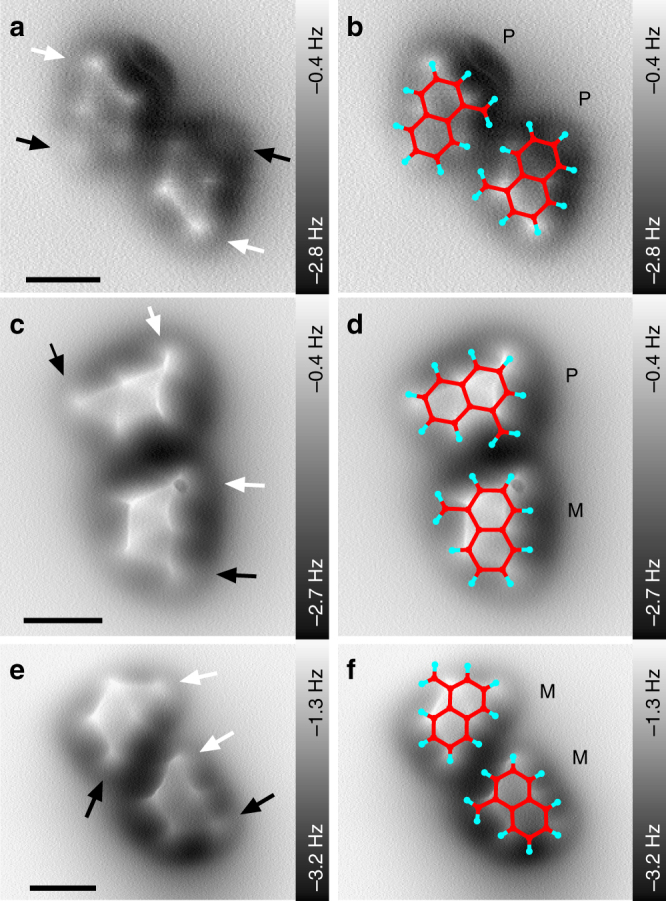
Constant height AFM scans of [123]tetramantane dimers. **a**, **b** (*P*,*P*)-[123]tetramantane dimers. **c**, **d** (*M*,*P*)-[123]tetramantane dimers. **e**, **f** (*M*,*M*)-[123]tetramantane dimers. Tilting of the molecules is indicated by black and white arrows (as in Fig. 4). **b**, **d**, **f** AFM scans overlaid with molecular structures. For clarity only the plane containing the two specific hydrogens is plotted (cf. Fig. 4). Scale bars: 0.5 nm

Next we applied the bond imaging method to different dimers and a small cluster of [123]tetramantanes. Fig. 5 depicts (*P*,*P*) (a, b), (*M*,*P*) (c, d), and (*M*,*M*) (e, f) dimers. The absolute configuration of each molecule was identified by the observed molecular tilting with regard to the *x*/*y* plane (see black and white arrows). Overlays with the corresponding molecular structures are depicted in the right column of Fig. 5b, d, f. In the case of molecular dimers the Olympic ring patterns of the individual molecules also align with the crystallographic [1–10] direction (see Supplementary Fig. 3). Precise knowledge about molecular orientation allows for determination of close contacts between two molecules within the dimers. Such information can also be used to quantify LD interactions between the molecules and pinpoint the onset of the crystallization process^36^. Using these structural data as a starting point, we performed a detailed computational study for two series of dispersion-bound complexes consisting of two enantiomers, (*M*,*P*) and (*M*,*M*)-[123]tetramantanes, respectively (details are provided in Supplementary Figs. 4, 5 Supplementary Tables 1–6). The obtained results confirmed that dimer formation is indeed an energetically favourable process driven by LD and the strength of these interactions was successfully quantified.

**Fig. 6:**
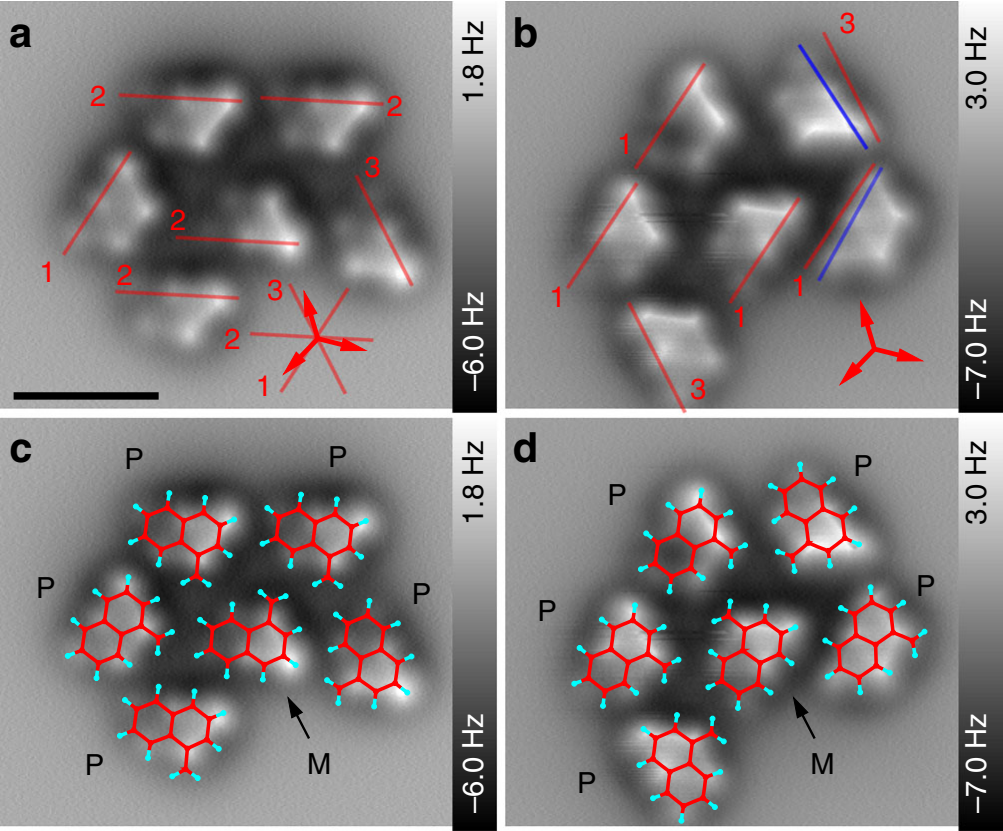
Constant height AFM scans of a molecular cluster consisting of [123]tetramantane. **a** Scan before manipulation by AFM tip. **b** Scan after manipulation by AFM tip. The numbered red lines indicate the three main directions of molecular alignment. The three red arrows indicate the crystallographic [0–11], [10–1], and [–110] directions. The two blue lines in **b** indicate molecules that were not rotated by inter multiples of 60°. **c**, **d** AFM scans overlaid with molecular structures. The cluster contains five (*P*)-enantiomers and one (*M*)-enantiomer. For clarity only the plane containing the two specific hydrogens is plotted (see Fig. 4). Scale bar: 1 nm

A cluster of six [123]tetramantanes is depicted in Fig. 6a and b. As in the previous figures, the AFM scans allow the assignment of absolute configuration and the precise determination of molecular orientation. An overlay of the molecular structures is shown in (c, d). Images (a, c) were taken before, while images (b, d) were obtained after deliberately manipulating the molecules with the AFM tip. Therefore, the molecular cluster has been imaged in STM mode while the gap voltage and tunneling current were systematically changed until manipulation of the molecules was observed. Fig. 6b reveals that five of the six molecules have been rotated by this procedure. Comparing the arrangement of the molecules before and after manipulation reveals very similar patterns of close contacts between the molecules, showing that intermolecular LD interactions have a significant influence on the process of molecular assembly.

Finally, we discuss how cluster formation depends on the interplay between intermolecular LD interactions and molecule–surface interactions. The observed snapping of single [123]tetramantanes and molecular dimers to the Cu lattice demonstrates that molecule–surface interactions play a significant role in on-surface assembly (see Supplementary Figs. 2, 3). The imaged molecular cluster, however, reveals a slight deviation regarding the molecular orientations, i.e., in Fig. 6a the three observed orientations of the Olympic ring patterns (see red lines indicated with numbers 1–3) are not perfectly parallel to the crystallographic [1–10], [10–1], and [01–1] directions (see three red arrows). This indicates that intermolecular LD interactions start to have an increased role in molecular assembly as more bulky molecules are being added to the cluster.

This observation is even more pronounced after deliberately manipulating the molecules with the CO tip. Fig. 6b shows that three out of five manipulated molecules are rotated by integer multiples of approx. 60°, while two molecules are found to be misaligned by a few degrees (see blue lines). The two blue lines show an even stronger deviation from the crystallographic [1–10] directions than the three red lines. Hence, for the studied molecular cluster intermolecular LD interactions start to dominate the assembling process, while molecule–surface interactions play here only a minor role. This is in line with our previous results, which show that LD interactions can direct the self-assembly of [121]tetramantane within larger clusters (approx. 10 molecules) and big islands^36^. Furthermore, the possibility to directly assign the precise orientation and the absolute configuration of the molecules paired with the manipulation capabilities of scanning probe techniques will allow future studies of chirality driven assembly mechanisms at a new level of accuracy.

To summarize, we assigned by direct visual inspection the absolute configuration and orientation of adsorbed [123]tetramantanes on Cu(111) using low temperature AFM with a CO functionalized tip. The approach was successfully applied to single molecules, molecular dimers, and small molecular clusters. We determined the intermolecular arrangement of enantiomers with atomic precision and supported the experimental findings with a systematic computational study. The present approach for assigning absolute configurations of chiral molecules can thus be considered as an emerging tool for studying molecular recognition and reactivity of chiral compounds on surfaces, capable of providing a new level of sophistication.

## Methods

### STM/AFM measurements

[123]Tetramantane was isolated from petroleum and purified by multiple HPLC separations as described previously^16^. We used an ultra-high vacuum low temperature STM/AFM (ScientaOmicron, Germany) with a base pressure below 1 × 10^−10^ mbar. The Cu(111) single crystal substrate (MaTecK, Germany) was cleaned prior to the experiment, by multiple (up to 100) cycles of Ar^•+^ sputtering (1.5 keV, 3–6 × 10^−6^ mbar, 3–4 μA) and subsequent annealing (up to 1000 K). For evaporation a small amount of molecule powder was inserted into a stainless steel tube that was attached to an Omicron sample plate and kept at room temperature^45^. The molecules were deposited onto the Cu surface (below 15 K) through the opened temperature shields of the STM/AFM instrument. All measurements were performed with the tip connected to ground and the sample connected to bias voltage at a temperature of 5 K. Commercial (ScientaOmicron) and homemade^46^ sensors with tungsten tips have been used (resonant frequency = 19.3 or 27.0 kHz, *Q*-factor = 15,000–30,000, oscillation amplitude = 60–160 pm), CO molecules have been picked up from the Cu surface using the recipe of Bartels et al.^47^ or by applying voltage pulses of 3–4 V.

### Computations

Semiempirical computations of [123]tetramantane molecules on a Cu(111) surface were performed using the GFN-xTB approach developed by Grimme et al^44^. A copper crystal surface cut-out with dimensions of 18 × 18 × 5 Å consisting of 216 Cu atoms was taken and the atoms were frozen to simulate the copper lattice. Tetramantane molecules of the corresponding orientation observed in the AFM images (Olympic rings, triangle, and rhombus) were placed on the copper slab, optimized and their frequency analyses were performed. The obtained geometries were then compared with the structural parameters obtained from AFM imaging.

### Data availability

The data supporting the findings of this study are available within the paper and its Supplementary Information files.

## Electronic supplementary material

Supplementary Information

Description of Additional Supplementary Files

Supplementary Data 1

Supplementary Data 2

Supplementary Data 3
