# Supplementary material for: Assigning the absolute configuration of single aliphatic molecules by visual inspection
Source: Nat Commun. 2018 Jun 20;9:2420. doi: 10.1038/s41467-018-04843-z (PMC6010418; doi:10.1038/s41467-018-04843-z)
Supplement: Supplementary file 1 — Supplementary Information [file 41467_2018_4843_MOESM1_ESM.docx]

**Supplementary Information**

**Assigning the absolute configuration of single aliphatic molecules by visual inspection**

Daniel Ebeling,^1,‡,*^ Marina Šekutor,^2,‡,*^ Marvin Stiefermann,^1^ Jalmar Tschakert,^1^ Jeremy E. P. Dahl,^3^ Robert M. K. Carlson,^3^ André Schirmeisen,^1,*^ and Peter R. Schreiner^2,*^

^1^Institute of Applied Physics, Justus-Liebig University, Heinrich-Buff-Ring 16, 35392 Giessen, Germany, [Daniel.Ebeling@ap.physik.uni-giessen.de](mailto:Daniel.Ebeling@ap.physik.uni-giessen.de), [Andre.Schirmeisen@ap.physik.uni-giessen.de](mailto:Andre.Schirmeisen@ap.physik.uni-giessen.de)

^2^Institute of Organic Chemistry, Justus-Liebig University, Heinrich-Buff-Ring 17, 35392 Giessen, Germany, [Marina.Sekutor@org.Chemie.uni-giessen.de](mailto:Marina.Sekutor@org.Chemie.uni-giessen.de), [prs@uni-giessen.de](mailto:prs@uni-giessen.de)

^3^Stanford Institute for Materials and Energy Sciences, Stanford, CA 94305, USA

^‡^Both contributors are considered first authors.

**Table of content**

| **Supplementary Discussion – AFM imaging** | 3 |
| --- | --- |
| **Supplementary Figure 1.** Top and side view of computed (*M*)- and (*P*)-[123]tetramantane on Cu(111). | 3 |
| **Supplementary Figure 2.** Constant height AFM scans of nine different [123]tetramantanes (6× (*M*)-type, 3× (*P*)-type). | 5 |
| **Supplementary Figure 3.** Constant height AFM scans of four different [123]tetramantane dimers. | 6 |
| **Supplementary Discussion – Computations** | 7 |
| **Supplementary Figure 4.** Optimized structures of (*M*)-[123]tetramantane LD-bound complexes (**M1**-**M3**) computed at the B3LYP-D3(BJ)/6-31G(d,p) level of theory. | 11 |
| **Supplementary Figure 5.** Optimized structures of (*M*)- and (*P*)-[123]tetramantane LD-bound complexes (**MP1**-**MP6**) computed at the B3LYP-D3(BJ)/6-31G(d,p) level of theory. | 12 |
| **Supplementary Table 1.** Interaction energies, Δ*H*(0 K), of LD-bound complexes of (*M*)-[123]tetramantane (**M1**‒**M3**) in kcal mol^−1^. | 13 |
| **Supplementary Table 2.** Interaction energies, Δ*H*(0 K), of LD-bound complexes of (*M*)- and (*P*)-[123]tetramantane (**MP1**‒**MP6**) in kcal mol^−1^. | 14 |
| **Supplementary Table 3.**  Electronic energies of (*M*)-[123]tetramantane (**1**) and LD-bound complexes of (*M*)-[123]tetramantane (**M1**‒**M3**) in hartree. | 15 |
| **Supplementary Table 4.** Electronic energies of LD-bound complexes of (*M*) and (*P*)-[123]tetramantane (**MP1**‒**MP6**) in hartree. | 16 |
| **Supplementary Table 5.** Zero-point vibrational energies (ZPVEs) of (*M*)-[123]tetramantane (**1**) and LD-bound complexes of (*M*)-[123]tetramantane (**M1**‒**M3**) and of (*M*)- and (*P*)-[123]tetramantane (**MP1**‒**MP6**) in hartree computed at the B3LYP-D3(BJ)/6-31G(d,p) level of theory. | 17 |
| **Supplementary Table 6.** Zero-point vibrational energies (ZPVEs) of (*M*)-[123]tetramantane (**1**) and LD-bound complexes of (*M*)-[123]tetramantane (**M1**‒**M3**) and of (*M*)- and (*P*)-[123]tetramantane (**MP1**‒**MP6**) in hartree computed at the M06-2X/6-31G(d,p) level of theory. | 17 |
| **Supplementary Table 7.** Energies of (*M*)-[123]tetramantane (**1**) on a Cu(111) surface computed using the GFN-xTB approach. | 18 |
| **Supplementary References** | 19 |

**Supplementary Discussion – AFM imaging**

**
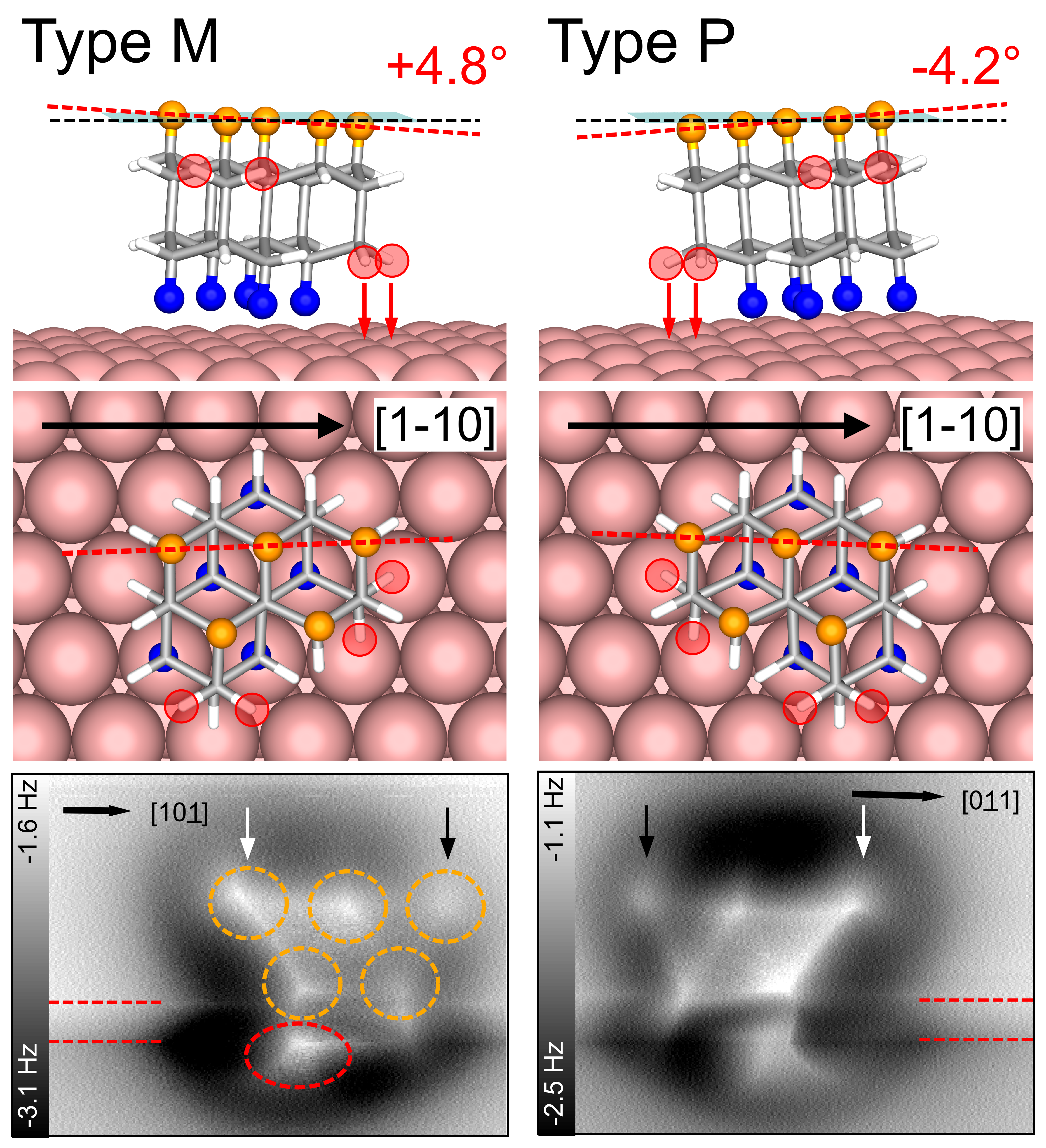
**

**Supplementary Figure 1.** Top and side view of computed (*M*)- and (*P*)-[123]tetramantane on Cu(111) (depicted in the first two rows). Hydrogens in the imaging and surface planes are displayed as orange and blue spheres. “Specific hydrogens” that are located either 130 below the orange or 130 pm above the blue Olympic ring patterns (see also Fig. 4 in the manuscript) are marked with red circles. The AFM images in the bottom row correspond to Figs. 4f and g, but are rotated by 180°. The two top views in the middle row reveal that the Olympic ring patterns of (*M*)- and (*P*)-type molecules align with the crystallographic [1-10] direction. Note that the shown computed configurations correspond to local minima on the potential energy surface. Since the experimentally observed tetramantanes always align with the [0–11], [10–1], and [–110] directions (see arrows in bottom row images and Supplementary Figure 2), we chose a computed configuration that was most similar to the experimental results. The two side views in the first row reveal that corresponding computed structures are tilted with regard to the x/y-plane by +4.8° ((*M*)-type) and –4.2° ((*P*)-type). This tilting is in agreement with the tilting of (*M*)- and (*P*)-type molecules in our AFM images. Presumably the observed tilting is caused by attractive forces between the two specific hydrogens and the Cu surface atoms (see two red arrows in the first row images).

**
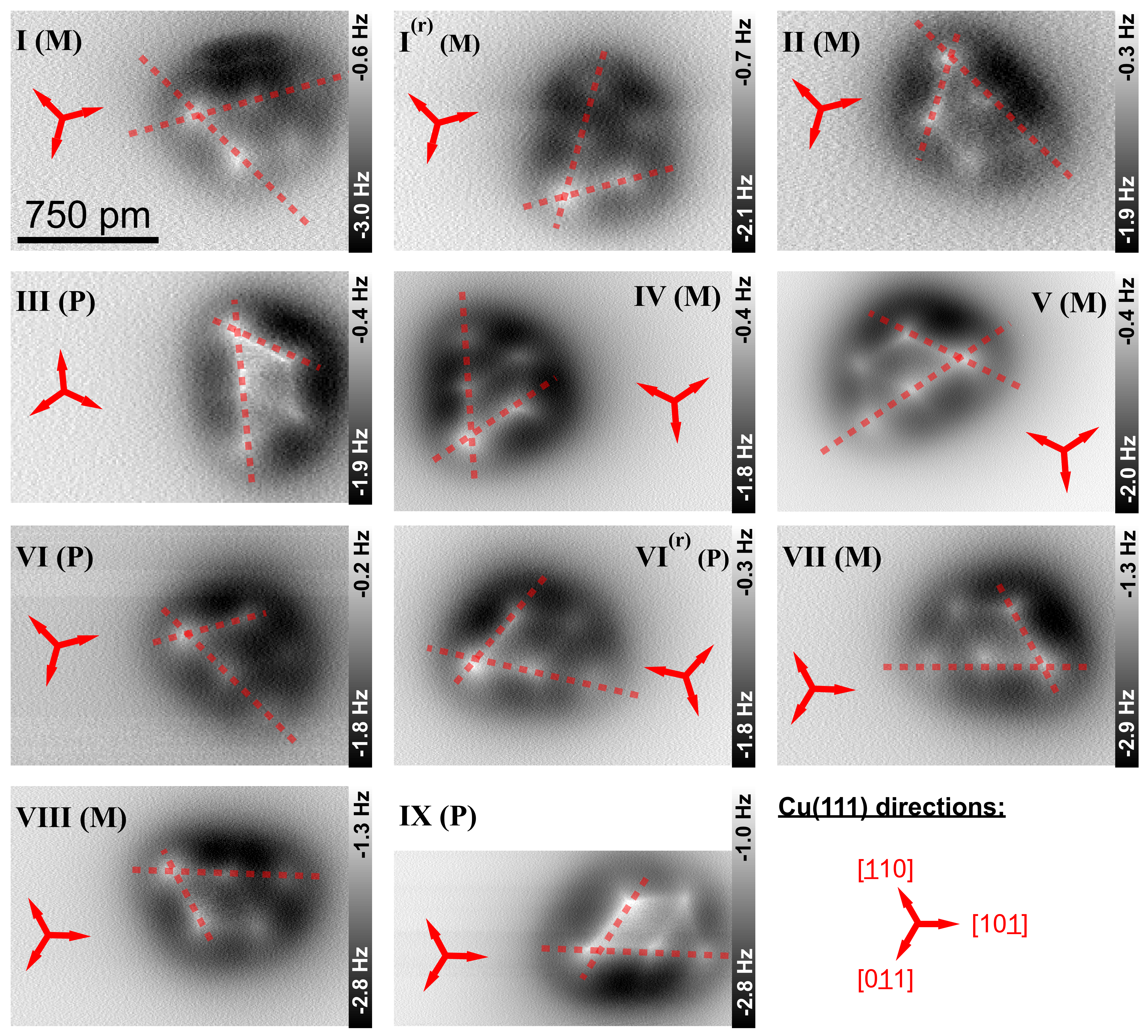
**

**Supplementary Figure 2.** Constant height AFM scans of nine different [123]tetramantanes (6× (*M*)-type, 3× (*P*)-type). Different molecules are indexed by Roman numerals. If a molecule has been imaged before and after manipulation with the CO tip, the rotated version is marked with an (r). Red arrows indicate substrate orientations [0–11], [10–1] and [–110], while dashed red lines are parallel to those directions. For all nine molecules the Olympic ring pattern aligns almost perfectly with one of those crystallographic directions. The crystallographic directions of our Cu single crystal were assigned using atomic resolution images of the Cu surface. Molecule VII is identical to the one shown in Fig. 3g.

**
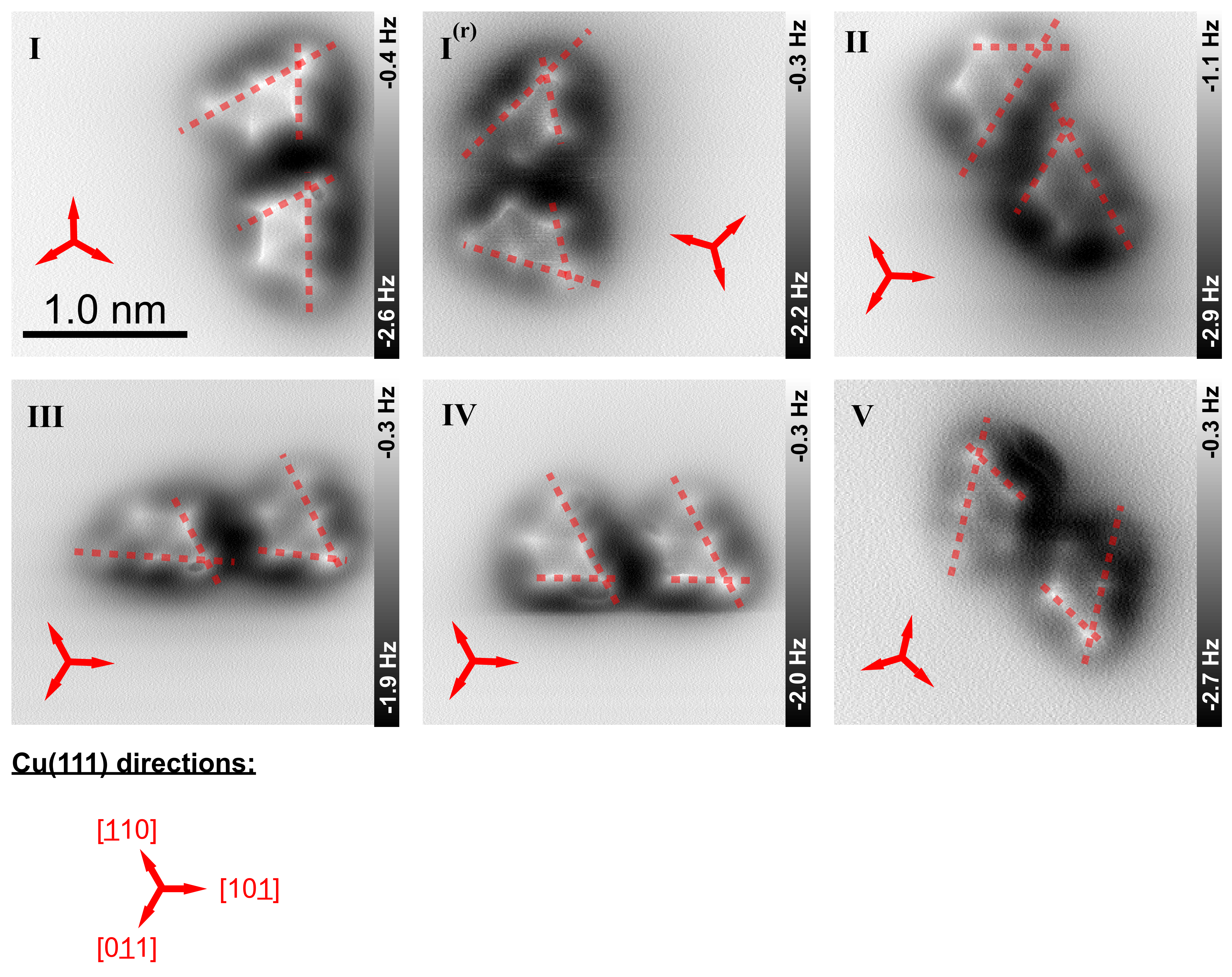
**

**Supplementary Figure 3.** Constant height AFM scans of five different [123]tetramantane dimers. Different dimers are indexed by Roman numerals. If a dimer has been imaged before and after manipulation with the CO tip, the rotated version is marked with an (r). Red arrows indicate substrate orientations [0–11], [10–1] and [–110], while dashed red lines are parallel to those directions. The point where the two dashed lines cross each other indicates the brightest part of the molecule, which can be used for identification of their handedness. For all ten molecules the Olympic ring pattern aligns almost perfectly with one of those crystallographic directions. Dimers I, II and V are identical to those shown in Figs. 5c, 5e and 5a, respectively.

**Supplementary Discussion – Computations**

Geometry optimizations were performed with the GAUSSIAN09 program package^1^ by taking the X-ray single-crystal structure of [123]tetramantane^2^ and molecule orientations observed by AFM imaging as a starting point for dimer computations. All structures were optimized using the B3LYP-D3(BJ)/6-31G(d,p) and M06-2X/6-31G(d,p) level of theory and the corresponding coordinates are available from the Supplementary Data file. Verification of the minima by frequency computations was performed and the energies given are corrected for zero-point vibrational energies (ZPVEs). Single-point energies on the B3LYP-D3(BJ)/6-31G(d,p)-optimized structures were computed using the ORCA 4.0.0 program^3^ at the RI-MP2/cc-pVDZ, RI-MP2/cc-pVTZ and DLPNO-CCSD(T)/cc-pVDZ levels of theory.

We have shown previously that the uncorrected B3LYP functional^4,5^ is unsuitable for computation of tetramantane hydrocarbons.^6^ Because this functional does not account for London dispersion (LD),^7-9^ it needs to be augmented by Grimme’s D3-dispersion correction^10^ in connection with Becke-Johnson (BJ) damping to avoid overbinding at short distances.^11,12^ A reliable level of theory to quantify LD contribution in tetramantanes was found to be B3LYP-D3(BJ)/6-31G(d,p), which we used to obtain all geometries of [123]tetramantane dimers presented herein. For comparison purposes, we also optimized the studied dimers using M06-2X/6-31G(d,p)^13^ level of theory which gave comparable structures. Accurate energies for the dimers were then computed using the complete basis set extrapolation method.^14^ For this purpose we used second order Møller–Plesset perturbation theory MP2^15^ and CCSD(T)^16^ *ab initio* method in conjunction with cc-pVDZ and cc-pVTZ basis sets for single-point computations, as described previously for [121]tetramantane dimers.^6^ All RI-MP2^17-20^ and DLPNO-CCSD(T)^21-23^ single-point computations were performed on optimized geometries obtained by B3LYP-D3(BJ)/6-31G(d,p) level of theory, and TightPNO accuracy settings were used for the DLPNO method.

In brief, the complete basis set extrapolation was performed by partitioning the total energy (Δ*E*_est_^CCSD(T)^) into the HF energy (Δ*E*_CBS_^HF^), the MP2 correlation energy (Δ*E*_CBS_^MP2,corr^) and into the contributions of higher order correlation effects (ΔCCSD(T)) (Supplementary Equations 1–5). Since MP2 and CCSD(T) correlation energies converge at a similar rate, it is possible to estimate the CCSD(T)/CBS energy using only moderately sized basis sets.^24^

In our case, for the cc-pVDZ and cc-pVTZ basis sets, the energy (Δ*E*_CBS_^HF^ and Δ*E*_CBS_^MP2,corr^, respectively) can be expressed as

${\Delta E}_{\mathrm{CBS}}^{\mathrm{HF}}= \frac{3^{\alpha}}{3^{\alpha}-2^{\alpha}} E_{3}^{\mathrm{HF}}- \frac{2^{\alpha}}{3^{\alpha}-2^{\alpha}} E_{2}^{\mathrm{HF}}$ Supplementary Equation 1

and

${\Delta E}_{\mathrm{CBS}}^{MP2, corr}= \frac{3^{\beta}}{3^{\beta}-2^{\beta}} E_{3}^{MP2, corr}- \frac{2^{\beta}}{3^{\beta}-2^{\beta}} E_{2}^{MP2, corr}$ Supplementary Equation 2

with the reported optimized exponent values α_23_ = 4.42 and β_23_ = 2.46.^25^

The basis set limit for MP2 is defined as

${\Delta E}_{\mathrm{CBS}}^{MP2}= {\Delta E}_{\mathrm{CBS}}^{\mathrm{HF}}+ {\Delta E}_{\mathrm{CBS}}^{MP2, corr}$ Supplementary Equation 3

and the estimate for the CCSD(T) energies corresponds to

${\Delta E}_{\mathrm{est}}^{CCSD(T)}= {\Delta E}_{\mathrm{CBS}}^{MP2}+ \Delta CCSD(T)$ Supplementary Equation 4

with ΔCCSD(T) equalling to

$\Delta CCSD(T)=(E^{\mathrm{CCSD}\left( T \right)}-E^{MP2})_{small basis set}$ Supplementary Equation 5

By applying this procedure we obtained RI-MP2/CBS and DLPNO-CCSD(T)/CBS interaction energies (Supplementary Tables 1 and 2 in parentheses). When adding the corresponding zero-point vibrational energies (ZPVEs) of (*M*)-[123]tetramantane (**1**), and the dimers **M1**‒**M3** and **MP1**‒**MP6** (computed at the B3LYP-D3(BJ)/6-31G(d,p) level of theory) to the computed RI-MP2 and DLPNO-CCSD(T) energies, Δ*H*(0 K) was determined (Supplementary Tables 1 and 2).

As expected, upon performing geometry optimization of enantiomers (*M*)-[123]tetramantane (**1**) and (*P*)-[123]tetramantane (**2**), we obtain analogous minima structures with same energies and enthalpies. Therefore we computed only selected LD-bound complexes of two (*M*)-[123]tetramantanes and LD-bound complexes of (*M*)- and (*P*)-[123]tetramantane, omitting computations for two (*P*)-[123]tetramantanes to avoid repetition. The structures of computed dimers are depicted in Supplementary Figures 4 and 5.

Before going into a detailed analysis of the computed structures, we first need to define structural markers of the [123]tetramantane cage that we will use in our discussion. [123]Tetramantane has a *C*_2_ symmetry and is helically chiral, resulting in *M* and *P* enantiomers.^2^ Chirality of the hydrocarbon cage becomes obvious when noticing a presence of a helical groove in the centre of the molecule and we will use it to define our first structural marker. When accounting for the nomenclature,^26^ carbon pairs 3, 22 and 12, 21 are placed on opposite adamantyl subcages and effectively form this groove.^27^ For our purposes, we will define a CH_2_ group with a carbon atom numbered as 22 and with two hydrogens, one pointing in the direction of the molecular groove and the other pointing in the opposite direction, as a ridge. Our second structural marker will be the five C‒H bonds that all point upward in the same direction (termed Olympic rings), with the five corresponding hydrogens forming a plane. As we will see, these markers are sufficient for comparing different orientations of the computed [123]tetramantane dimers.

Interaction energies obtained from the dispersion-corrected B3LYP method are in excellent agreement with the values from the MP2 approach (Supplementary Tables 1 and 2), further establishing the reliability of the used level of theory for large, bulky hydrocarbons.^6,28,29^ Although the MP2 method sometimes gives different interaction energies for dispersion-bound complexes when compared to CCSD(T),^30^ the values we obtained still make a good match and follow the same general trend. Local Energy Decomposition (LED) analysis^31^ gave values for dispersion contributions that are in line with other results presented herein, confirming the validity of our approach both for LD-bound complexes of [123]tetramantane as well as for complexes of [121]tetramantane described previously.^6^ To sum up, interaction energies for the most stable dimers are approaching 5 kcal mol^‒1^ and even though energy values differ slightly depending on the method used, the trends remain the same.

In continuation we will discuss dimer stabilities by taking into account intermolecular close contacts as well as the computed interaction energies. First we computed dimers consisting of two (*M*)-[123]tetramantanes, **M1**‒**M3** (Supplementary Figure 4, Supplementary Table 1). Orientations of **M1** and **M2** were found to be energetically more favourable due to the existence of a significant number of contacts between the cages. In contrast, only limited interaction in **M3** resulted in the least stable structure in the series. This trend points towards a LD-driven orienting of two bulky hydrocarbons in the gas phase. Both **M1** and **M3** feature the five C‒H bonds pointing upwards and forming a plane consisting of hydrogens of both molecules, albeit in case of **M1** a slight tilt between the cages exists. The difference in their energy arises from lessened interaction capability of **M3**. Additionally, in **M1** the respective ridges are placed on the opposite sides along the contact area, ensuring a good geometrical fit. Interaction in **M2** is accomplished along the five C‒H bond-area of both molecules, making this geometry less suitable to form on a metal surface, even though it is stable in the gas phase.

For **MP1**‒**MP6** dimers we found that, again, the most stable structures engage in numerous close contacts between the cages and the two molecules are preferentially oriented to have C‒H bond-rich areas adjacent (Supplementary Figure 5, Supplementary Table 2). The most stable dimer in the computed series was **MP2** that exploits the attracting potential of the ridges belonging to the two cages. The ridge areas are situated one next to the other and the five C‒H bonds of both hydrocarbons are pointing outwards, forming a plane that would enable the dimer to sit flatly on a metal surface. In contrast to **M1** where the ridges are placed opposite since two equal (*M*) enantiomers would not benefit from such close, borderline repulsive contact between the ridges, in **MP2** no crowding occurs since the dimer is composed of geometrically non-complementary *M* and *P* molecules. LD-bound complex **MP4** is also a very stable structure but, unlike **MP2**, here the interacting area is the two five C‒H bond region of both molecules. Due to so many interactions, this orientation is energetically favourable in the gas phase, although it may not be perfectly suited for on-surface deposition. Dimers **MP3**, **MP5** and **MP6** are comparable in energy and are also favourable structures. Complex **MP3** is structurally very similar to **MP4**, the only difference being that in **MP3** the cages are somewhat shifted in plane that goes along the five C‒H bond area. Both **MP5** and **MP6** are oriented in such a way that the five C‒H bonds point upwards, but the groves are not interacting with each other (a head-to-tail cage orientation). In **MP6** the cages are somewhat tilted compared to **MP5**. The least stable computed dimer is **MP1** in which the cages have only a limited number of close contacts and C‒H bond-rich areas of the hydrocarbons are facing away from each other. In this orientation [123]tetramantanes are markedly tilted and the complex lacks intermolecular stabilization by beneficial LD forces, making it an improbable candidate for formation on a metal surface.

When comparing the computed dimer orientations with the observed structures on the Cu(111) surface, it becomes apparent that the geometries where the cages sit on the surface along the five C‒H bond-plane are preferred. From the AFM images we could identify **MP2**, **MP5**, **MP6**, and **M3** orientations of [123]tetramantanes. As noted, these *M* and *P* LD-bound complexes have advantageous energetic gain upon complexations and are therefore accordingly formed on surface. A somewhat surprising finding was the discovery of the **M3** geometry on Cu(111) since this dimer was found to be of relatively lesser stability. However, this once more underlines that the surface itself is not just a passive bystander in the complexation process and affects the behaviour of molecules in UHV conditions, especially when the hydrocarbons do not engage in stronger interaction necessary for forming a 2D monolayer. Another point to consider is also the probability of finding a certain dimer structure in the AFM scanned area under study, meaning that other dimers are also probable on the surface but might not be found due to simple statistical chance.

In conclusion, we found that LD interactions stabilize complexes formed by two [123]tetramantane molecules and that the energetic gain upon complexation is of the order of magnitude of 5 kcal mol^‒1^. This is less than the stabilization for [121]tetramantane complexes, that we found to be of the order of magnitude of 10 kcal mol^‒1^.^6^ The reason for this difference lies in the structural nature of [121] vs. [123]tetramantane. [121]Tetramantane is a non-chiral, rod-shaped hydrocarbon with a very compact structure and it can therefore easily stack and create a uniform 2D lattice on a metal surface. On the other hand, [123]tetramantane has a helical twist and it can arrange with less efficacy, regardless if the pair is a (*M*,*M*) or a (*M*,*P*) complex. This is reflected in interaction energies since two [123]tetramantane molecules can interact in a number of different ways without a drastic change in the energetic gain. Another confirmation of its scaled down intermolecular stabilization is the observed formation of dimer pairs on a Cu(111) surface and not a periodic monolayer of hydrocarbons, as was the case for [121]tetramantane in the same experimental conditions.

| **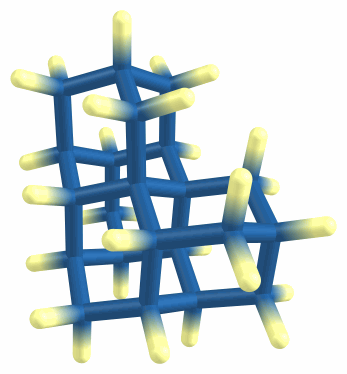** | **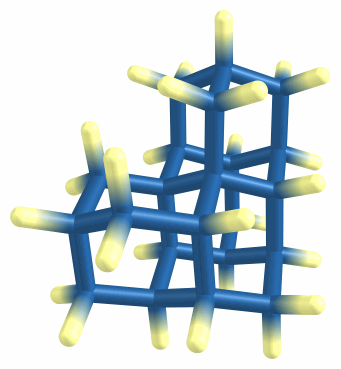** |  |
| --- | --- | --- |
| **1** | **2** |  |
| **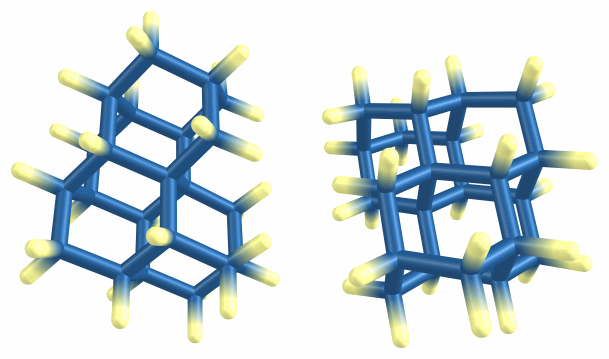** | **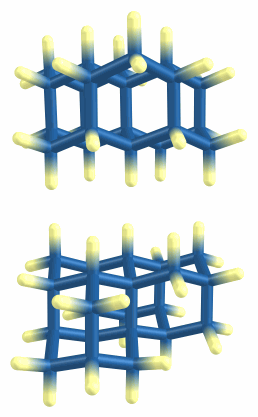** | **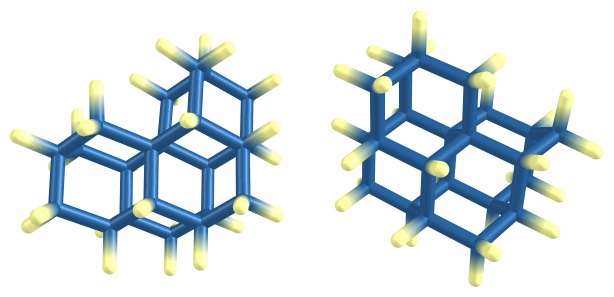** |
| **M1** | **M2** | **M3** |

**Supplementary Figure 4.** Optimized structures of (*M*)-[123]tetramantane (**1**), (*P*)-[123]tetramantane (**2**) and (*M*)-[123]tetramantane LD-bound complexes (**M1**-**M3**) computed at the B3LYP-D3(BJ)/6-31G(d,p) level of theory.

| **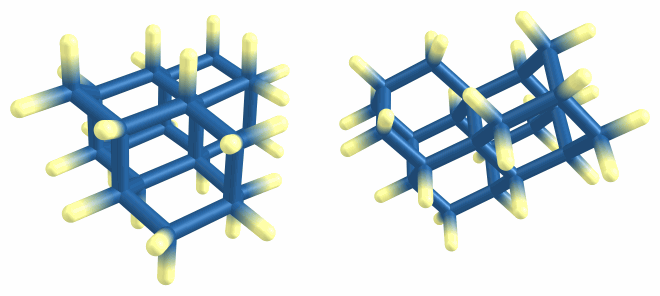** | **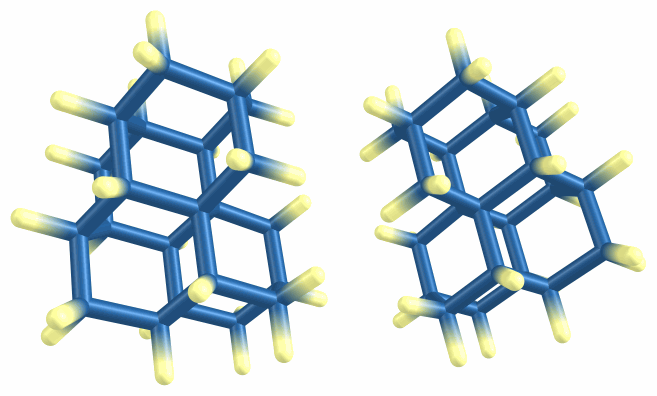** | **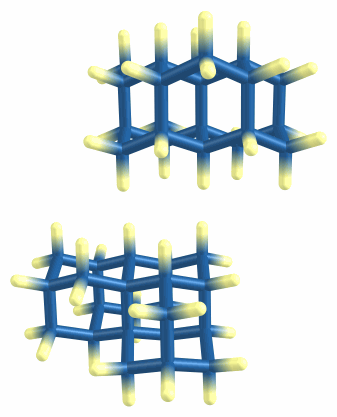** |
| --- | --- | --- |
| **MP1** | **MP2** | **MP3** |
| **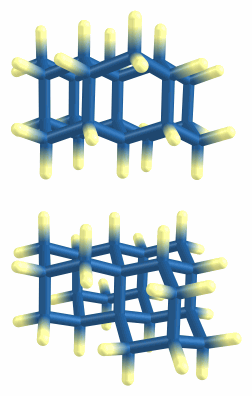** | **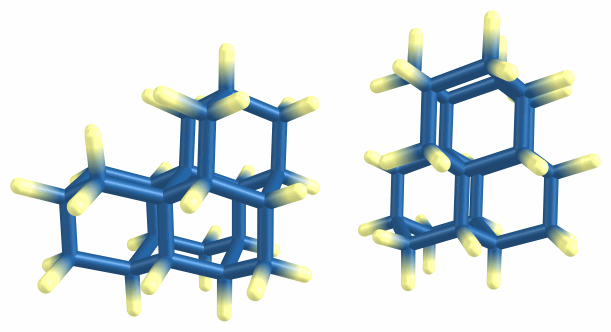** | **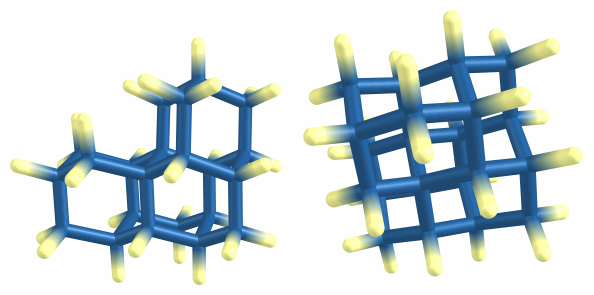** |
| **MP4** | **MP5** | **MP6** |

**Supplementary Figure 5.** Optimized structures of (*M*)- and (*P*)-[123]tetramantane LD-bound complexes (**MP1**-**MP6**) computed at the B3LYP-D3(BJ)/6-31G(d,p) level of theory.

**Supplementary Table 1.** Interaction energies, Δ*H*(0 K), of LD-bound complexes of (*M*)-[123]tetramantane (**M1**‒**M3**) in kcal mol^−1^. Geometries for RI-MP2 and DLPNO-CCSD(T) single-point computations were optimized at the B3LYP-D3(BJ)/6-31G(d,p) level of theory.^a, b, c^

| Level of theory | M1 | M2 | M3 |
| --- | --- | --- | --- |
| B3LYP-D3(BJ)/6-31G(d,p) | ‒7.5 (‒8.1) | ‒7.7 (‒8.3) | ‒5.5 (‒5.9) |
| M06-2X/6-31G(d,p) | ‒5.5 (‒5.7) | ‒4.6 (‒4.8) | ‒3.7 (‒3.9) |
| RI-MP2/cc-pVDZ | ‒6.8 (‒7.3) | ‒6.5 (‒7.1) | ‒4.5 (‒5.0) |
| RI-MP2/cc-pVTZ | ‒7.4 (‒7.9) | ‒7.5 (‒8.0) | ‒5.3 (‒5.7) |
| RI-MP2/CBS | ‒7.9 (‒8.4) | ‒8.2 (‒8.7) | ‒5.8 (‒6.3) |
| DLPNO-CCSD(T)/cc-pVDZ | ‒5.5 (‒6.1) | ‒5.1 (‒5.7) | ‒3.6 (‒4.0) |
| DLPNO-CCSD(T)/CBS | ‒6.6 (‒7.1) | ‒6.8 (‒7.4) | ‒4.9 (‒5.3) |
| LED – dispersion (CCSD)^d^ | (‒6.3) | (‒6.6) | (‒4.5) |

^a^ Interaction energies are defined as a difference between the energy of the dimer and the energy of two (*M*)-[123]tetramantane molecules.
^b^ Values in parentheses correspond to computed Δ*E* in kcal mol^−1^. ^c^ ZPVEs used to obtain Δ*H*(0 K) for RI-MP2 and DLPNO-CCSD(T) computed at the B3LYP-D3(BJ)/6-31G(d,p) level of theory. ^d^ Local Energy Decomposition analysis accounting for dispersion contributions implemented in ORCA 4.0.0 for the DLPNO-CCSD(T) method.

**Supplementary Table 2.** Interaction energies, Δ*H*(0 K), of LD-bound complexes of (*M*)- and (*P*)-[123]tetramantane (**MP1**‒**MP6**) in kcal mol^−1^. Geometries for RI-MP2 and DLPNO-CCSD(T) single-point computations were optimized at the B3LYP-D3(BJ)/6-31G(d,p) level of theory.^a, b, c^

| Level of theory | MP1 | MP2 | MP3 | MP4 | MP5 | MP6 |
| --- | --- | --- | --- | --- | --- | --- |
| B3LYP-D3(BJ)/6-31G(d,p) | ‒5.1 (‒5.4) | ‒7.7 (‒8.4) | ‒6.4 (‒7.0) | ‒7.7 (‒8.3) | ‒6.2 (‒6.7) | ‒6.4 (‒6.8) |
| M06-2X/6-31G(d,p) | ‒3.4 (‒3.5) | ‒5.9 (‒6.4) | ‒3.5 (‒3.7) | ‒4.7 (‒4.7) | ‒4.0 (‒4.6) | ‒4.2 (‒4.4) |
| RI-MP2/cc-pVDZ | ‒4.2 (‒4.6) | ‒6.9 (‒7.5) | ‒5.4 (‒5.9) | ‒6.4 (‒7.0) | ‒5.5 (‒5.9) | ‒5.4 (‒5.8) |
| RI-MP2/cc-pVTZ | ‒4.8 (‒5.2) | ‒7.7 (‒8.3) | ‒6.1 (‒6.6) | ‒7.4 (‒8.0) | ‒6.0 (‒6.5) | ‒6.2 (‒6.6) |
| RI-MP2/CBS | ‒5.2 (‒5.6) | ‒8.3 (‒8.9) | ‒6.7 (‒7.2) | ‒8.1 (‒8.7) | ‒6.5 (‒7.0) | ‒6.7 (‒7.1) |
| DLPNO-CCSD(T)/cc-pVDZ | ‒3.4 (‒3.8) | ‒5.5 (‒6.1) | ‒4.2 (‒4.8) | ‒5.0 (‒5.6) | ‒4.4 (‒4.9) | ‒4.3 (‒4.7) |
| DLPNO-CCSD(T)/CBS | ‒4.4 (‒4.8) | ‒6.9 (‒7.5) | ‒5.5 (‒6.0) | ‒6.7 (‒7.3) | ‒5.5 (‒6.0) | ‒5.6 (‒6.0) |
| LED – dispersion (CCSD)^d^ | (‒4.0) | (‒6.6) | (‒5.2) | (‒6.6) | (‒4.9) | (‒5.1) |

^a^ Interaction energies are defined as a difference between the energy of the dimer and the energy of two (*M*)-[123]tetramantane molecules.
^b^ Values in parentheses correspond to computed Δ*E* in kcal mol^−1^. ^c^ ZPVEs used to obtain Δ*H*(0 K) for RI-MP2 and DLPNO-CCSD(T) computed at the B3LYP-D3(BJ)/6-31G(d,p) level of theory. ^d^ Local Energy Decomposition analysis accounting for dispersion contributions implemented in ORCA 4.0.0 for the DLPNO-CCSD(T) method.

**Supplementary Table 3.** Electronic energies of (*M*)-[123]tetramantane (**1**) and LD-bound complexes of (*M*)-[123]tetramantane (**M1**‒**M3**) in hartree.^a^

| Level of theory | 1 | M1 | M2 | M3 |
| --- | --- | --- | --- | --- |
| B3LYP-D3(BJ)/6-31G(d,p) | ‒855.445530 | ‒1710.903951 | ‒1710.904257 | ‒1710.900440 |
| M06-2X/6-31G(d,p) | ‒854.961980 | ‒1709.933109 | ‒1709.931540 | ‒1709.930234 |
| RI-MP2/cc-pVDZ | ‒852.601266 | ‒1705.214207 | ‒1705.213773 | ‒1705.210471 |
| RI-MP2/cc-pVTZ | ‒853.428223 | ‒1706.869049 | ‒1706.869233 | ‒1706.865557 |
| RI-MP2/CBS | ‒853.838985 | ‒1707.691357 | ‒1707.691899 | ‒1707.687936 |
| DLPNO-CCSD(T)/cc-pVDZ | ‒852.887052 | ‒1705.783785 | ‒1705.783143 | ‒1705.780536 |
| DLPNO-CCSD(T)/CBS | ‒854.124771 | ‒1708.260935 | ‒1708.261269 | ‒1708.258002 |

^a^ Values for RI-MP2/CBS and DLPNO-CCSD(T)/CBS obtained from Supplementary Equations 3 and 4.

**Supplementary Table 4.** Electronic energies of LD-bound complexes of (*M*)- and (*P*)-[123]tetramantane (**MP1**‒**MP6**) in hartree.^a^

| Level of theory | MP1 | MP2 | MP3 | MP4 | MP5 | MP6 |
| --- | --- | --- | --- | --- | --- | --- |
| B3LYP-D3(BJ)/6-31G(d,p) | ‒1710.899735 | ‒1710.904385 | ‒1710.902199 | ‒1710.904254 | ‒1710.901719 | ‒1710.901863 |
| M06-2X/6-31G(d,p) | ‒1709.929553 | ‒1709.934125 | ‒1709.929866 | ‒1709.931463 | ‒1709.931370 | ‒1709.931044 |
| RI-MP2/cc-pVDZ | ‒1705.209849 | ‒1705.214549 | ‒1705.212010 | ‒1705.213749 | ‒1705.212013 | ‒1705.211854 |
| RI-MP2/cc-pVTZ | ‒1706.864672 | ‒1706.869733 | ‒1706.867040 | ‒1706.869199 | ‒1706.866844 | ‒1706.866945 |
| RI-MP2/CBS | ‒1707.686878 | ‒1707.692208 | ‒1707.689440 | ‒1707.691858 | ‒1707.689123 | ‒1707.689339 |
| DLPNO-CCSD(T)/cc-pVDZ | ‒1705.780102 | ‒1705.783903 | ‒1705.781713 | ‒1705.783091 | ‒1705.781932 | ‒1705.781606 |
| DLPNO-CCSD(T)/CBS | ‒1708.257131 | ‒1708.261562 | ‒1708.259143 | ‒1708.261201 | ‒1708.259042 | ‒1708.259091 |

^a^ Values for RI-MP2/CBS and DLPNO-CCSD(T)/CBS obtained from Supplementary Equations 3 and 4.

**Supplementary Table 5.** Zero-point vibrational energies (ZPVEs) of (*M*)-[123]tetramantane (**1**) and LD-bound complexes of (*M*)-[123]tetramantane (**M1**‒**M3**) and of (*M*)- and (*P*)-[123]tetramantane (**MP1**‒**MP6**) in hartree computed at the B3LYP-D3(BJ)/6-31G(d,p) level of theory.

| structure | ZPVE |  |  | structure | ZPVE |
| --- | --- | --- | --- | --- | --- |
| 1 | 0.462240 |  |  | **MP2** | 0.925558 |
| M1 | 0.925350 |  |  | **MP3** | 0.925344 |
| M2 | 0.925365 |  |  | **MP4** | 0.925460 |
| M3 | 0.925173 |  |  | **MP5** | 0.925254 |
| MP1 | 0.925044 |  |  | **MP6** | 0.925121 |

**Supplementary Table 6.** Zero-point vibrational energies (ZPVEs) of (*M*)-[123]tetramantane (**1**) and LD-bound complexes of (*M*)-[123]tetramantane (**M1**‒**M3**) and of (*M*)- and (*P*)-[123]tetramantane (**MP1**‒**MP6**) in hartree computed at the M06-2X/6-31G(d,p) level of theory.

| structure | ZPVE |  |  | structure | ZPVE |
| --- | --- | --- | --- | --- | --- |
| 1 | 0.465069 |  |  | **MP2** | 0.930945 |
| M1 | 0.930481 |  |  | **MP3** | 0.930497 |
| M2 | 0.930462 |  |  | **MP4** | 0.930086 |
| M3 | 0.930518 |  |  | **MP5** | 0.929605 |
| MP1 | 0.930236 |  |  | **MP6** | 0.930552 |

**Supplementary Table 7.** Energies of (*M*)-[123]tetramantane (**1**) on a Cu(111) surface computed using the GFN-xTB approach.^32^ Different orientations of **1** (Olympic rings, triangle and rhombus, **1a**, **1b** and **1c**, respectively) were modelled on a Cu(111) slab consisting of 216 copper atoms at 15 K.

| structure | *E*  / hartree | *G*(15 K)  / hartree | Δ*G*(15 K)^a,b^  / kcal mol^–1^ | Δ*G*_rel_(15 K)^a,c^  / kcal mol^–1^ |
| --- | --- | --- | --- | --- |
| 1 | –61.823987 | –61.405164 | –^d^ | –^d^ |
| Cu(111) slab | –984.316330 | –984.362010 | –^d^ | –^d^ |
| 1a on Cu(111) | –1046.209979 | –1045.815391 | –30.3 (–43.7) | 0.0 (0.0) |
| 1b on Cu(111) | –1046.192259 | –1045.797770 | –19.2 (–32.6) | 11.1 (11.1) |
| 1c on Cu(111) | –1046.195052 | –1045.799195 | –20.1 (–34.3) | 10.2 (9.4) |

^a^ Values in parentheses correspond to the computed electronic energies.

^b^ Energies obtained by subtracting the energies of the isolated (*M*)-[123]tetramantane (**1**) and the Cu(111) slab from the energies of orientations **1a**, **1b** and **1c**, respectively.

^c^ Relative energies of different orientations of (*M*)-[123]tetramantane (**1**) on Cu(111) with structure **1a** taken as an reference point.

^d^ Not applicable.

**Supplementary References**

1 Frisch, M. J. *et al.* Gaussian 09 (Gaussian, Inc., Wallingford, CT, USA, 2013).

2 Schreiner, P. R. *et al.* [123]Tetramantane: parent of a new family of σ-helicenes. *J. Am. Chem. Soc.* **131**, 11292‒11293 (2009).

3 Neese, F. The ORCA program system. *WIREs Comput. Mol. Sci.* **2**, 73‒78 (2012).

4 Becke, A. D. Density-functional thermochemistry. III. The role of exact exchange. *J. Chem. Phys.* **98**, 5648‒5652 (1993).

5 Lee, C., Yang, W. & Parr, R. G. Development of the Colle-Salvetti correlation-energy formula into a functional of the electron density. *Phys. Rev. B* **37**, 785‒789 (1988).

6 Ebeling, D. *et al.* London dispersion directs on-surface self-assembly of [121]tetramantane molecules. *ACS Nano* **11**, 9459‒9466 (2017).

7 Kristyán, S. & Pulay, P. Can (semi)local density functional theory account for the London dispersion forces? *Chem. Phys. Lett.* **229**, 175‒180 (1994).

8 Hobza, P., Šponer, J. & Reschel, T. Density functional theory and molecular clusters. *J. Comput. Chem.* **16**, 1315‒1325 (1995).

9 J. M. Perez-Jorda & Becke, A. D. A density-functional study of van der Waals forces: rare gas diatomics. *Chem. Phys. Lett.* **233**, 134‒137 (1995).

10 Grimme, S., Antony, J., Ehrlich, S. & Krieg, H. A consistent and accurate *ab initio* parametrization of density functional dispersion correction (DFT-D) for the 94 elements H-Pu. *J. Chem. Phys.* **132**, 154104 (2010).

11 Grimme, S., Ehrlich, S. & Goerigk, L. Effect of the damping function in dispersion corrected density functional theory. *J. Comput. Chem.* **32**, 1456‒1465 (2011).

12 Grimme, S. Density functional theory with London dispersion corrections. *WIREs Comput. Mol. Sci.* **1**, 211‒228 (2011).

13 Zhao, Y. & Truhlar, D. G. Density Functionals with broad applicability in chemistry. *Acc. Chem. Res.* **41**, 157‒167 (2008).

14 Truhlar, D. G. Basis-set extrapolation. *Chem. Phys. Lett.* **294**, 45‒48 (1998).

15 Head-Gordon, M., Pople, J. A. & Frisch, M. J. MP2 energy evaluation by direct methods. *Chem. Phys. Lett.* **153**, 503‒506 (1988).

16 Pople, J. A., Head-Gordon, M. & Raghavachari, K. Quadratic configuration interaction. A general technique for determining electron correlation energies. *J. Chem. Phys.* **87**, 5968‒5975 (1987).

17 Weigend, F., Häser, M., Patzelt, H. & Ahlrichs, R. RI-MP2: optimized auxiliary basis sets and demonstration of efficiency. *Chem. Phys. Lett.* **294**, 143‒152 (1998).

18 Weigend, F. & Häser, M. RI-MP2: first derivatives and global consistency. *Theor. Chem. Acc.* **97**, 331‒340 (1997).

19 Bernholdt, D. E. & Harrison, R. J. Large-scale correlated electronic structure calculations: the RI-MP2 method on parallel computers. *Chem. Phys. Lett.* **250**, 477‒484 (1996).

20 Feyereisen, M., Fitzgerald, G. & Komornicki, A. Use of approximate integrals in *ab initio* theory. An application in MP2 energy calculations. *Chem. Phys. Lett.* **208**, 359‒363 (1993).

21 Riplinger, C. & Neese, F. An efficient and near linear scaling pair natural orbital based local coupled cluster method. *J. Chem. Phys.* **138**, 034106 (2013).

22 Riplinger, C., Sandhoefer, B., Hansen, A. & Neese, F. Natural triple excitations in local coupled cluster calculations with pair natural orbitals. *J. Chem. Phys.* **139**, 134101 (2013).

23 Riplinger, C., Pinski, P., Becker, U., Valeev, E. F. & Neese, F. Sparse maps – A systematic infrastructure for reduced-scaling electronic structure methods. II. Linear scaling domain based pair natural orbital coupled cluster theory. *J. Chem. Phys.* **144**, 024109 (2016).

24 Riley, K. E. & Hobza, P. Noncovalent interactions in biochemistry. *WIREs Comput. Mol. Sci.* **1**, 3‒17 (2011).

25 Neese, F. & Valeev, E. F. Revisiting the atomic natural orbital approach for basis sets: robust systematic basis sets for explicitly correlated and conventional correlated *ab initio* methods? *J. Chem. Theory Comput.* **7**, 33‒43 (2011).

26 Balaban, A. T. & Schleyer, P. v. R. Systematic classification and nomenclature of diamond hydrocarbons–I. *Tetrahedron* **34**, 3599‒3609 (1978).

27 Balaban, A. T. *et al.* NMR spectral properties of the tetramantanes – nanometer-sized diamondoids. *Magn. Reson. Chem.* **53**, 1003‒1018 (2015).

28 Wagner, J. P. & Schreiner, P. R. Nature utilizes unusual high London dispersion interactions for compact membranes composed of molecular ladders. *J. Chem. Theory Comput.* **10**, 1353‒1358 (2014).

29 Wang, C. *et al.* The self-association of graphane is driven by London dispersion and enhanced orbital interactions. *J. Chem. Theory Comput.* **11**, 1621‒1630 (2015).

30 Cybulski, S. M. & Lytle, M. L. The origin of deficiency of the supermolecule second-order Møller-Plesset approach for evaluating interaction energies. *J. Chem. Phys.* **127**, 141102 (2007).

31 Schneider, W. B. *et al.* Decomposition of intermolecular interaction energies within the local pair natural orbital coupled cluster framework. *J. Chem. Theory Comput.* **12**, 4778‒4792 (2016).

32 Grimme, S., Bannwarth, C. & Shushkov, P. A robust and accurate tight-binding quantum chemical method for structures, vibrational frequencies, and noncovalent interactions of large molecular systems parametrized for all spd-block elements (Z = 1–86). *J. Chem. Theory Comput.* **13**, 1989‒2009 (2017).
