# Supplementary material for: Assigning the absolute configuration of single aliphatic molecules by visual inspection
Source: Nat Commun. 2018 Jun 20;9:2420. doi: 10.1038/s41467-018-04843-z (PMC6010418; doi:10.1038/s41467-018-04843-z)
Supplement: Supplementary file 2 — Description of Additional Supplementary Files [file 41467_2018_4843_MOESM2_ESM.pdf]

Descriptions of Additional Supplementary Files

File Name: Supplementary Dataset 1
Description: Supplementary Dataset 1 contains geometries in Cartesian coordinates in Å of [123]tetramantane and its dimers computed at the B3LYP-D3(BJ)/6-31G(d,p) level of theory.

File Name: Supplementary Dataset 2
Description: Supplementary Dataset 2 contains geometries in Cartesian coordinates in Å of [123]tetramantane and its dimers computed at the M06-2X/6-31G(d,p) level of theory.

File Name: Supplementary Dataset 3
Description: Supplementary Dataset 3 contains geometries in Cartesian coordinates in Å of different orientations of (M)-[123]tetramantane (Olympic rings, triangle and rhombus, 1a, 1b and 1c, respectively) computed using the GFN-xTB approach that were modelled on a Cu(111) slab consisting of 216 copper atoms at 15 K.
