# Supplementary material for: Assigning the absolute configuration of single aliphatic molecules by visual inspection
Source: Nat Commun. 2018 Jun 20;9:2420. doi: 10.1038/s41467-018-04843-z (PMC6010418; doi:10.1038/s41467-018-04843-z)
Supplement: Supplementary file 3 — Supplementary Data 1 [file 41467_2018_4843_MOESM3_ESM.pdf]

Supplementary Data 1

Assigning the absolute configuration of single aliphatic molecules by visual inspection

Daniel Ebeling,[1,‡,] Marina Šekutor,[2,‡,*] Marvin Stiefermann,[1] Jalmar Tschakert,[1] Jeremy E. P. Dahl,[3] Robert M. K. Carlson,[3] André Schirmeisen,[1,*] and Peter R. Schreiner[2,*]*

[1]Institute of Applied Physics, Justus-Liebig University, Heinrich-Buff-Ring 16, 35392 Giessen, Germany, Daniel.Ebeling@ap.physik.uni-giessen.de, Andre.Schirmeisen@ap.physik.uni-giessen.de

[2]Institute of Organic Chemistry, Justus-Liebig University, Heinrich-Buff-Ring 17, 35392 Giessen, Germany, Marina.Sekutor@org.Chemie.uni-giessen.de, prs@uni-giessen.de

[3]Stanford Institute for Materials and Energy Sciences, Stanford, CA 94305, USA

[‡]Both contributors are considered first authors.

Supplementary Data Set 1. Geometries in Cartesian coordinates in Å computed at the B3LYP-D3(BJ)/6-31G(d,p) level of theory.

**1**			
1	-1.473315000	1.988168000	-0.812104000
6	0.629903000	-0.464365000	0.473525000
6	-0.629903000	0.464365000	0.473525000
6	-0.600037000	1.419624000	1.685733000
1	-0.661439000	0.867209000	2.626883000
1	-1.488062000	2.065082000	1.646720000
6	0.674523000	2.281164000	1.679628000
1	0.681296000	2.928291000	2.565243000
6	0.696793000	3.139154000	0.407047000
1	1.592371000	3.773413000	0.390505000
1	-0.173265000	3.807880000	0.385598000
6	0.680442000	2.216435000	-0.816362000
1	0.688234000	2.819479000	-1.734774000
6	-0.586718000	1.336601000	-0.815806000
6	-0.627619000	0.448594000	-2.070741000
1	-0.611600000	1.090955000	-2.961800000
6	-1.901945000	-0.401404000	-2.065185000
1	-2.785901000	0.249301000	-2.076704000
1	-1.948331000	-1.023477000	-2.968370000
6	-1.916408000	-1.289430000	-0.816930000
1	-2.825450000	-1.905960000	-0.812949000
6	-1.892782000	-0.435316000	0.472811000
1	-2.780914000	0.212570000	0.488606000
6	-1.916408000	-1.371241000	1.694938000
1	-2.825706000	-1.984854000	1.663929000
1	-1.959382000	-0.799004000	2.627270000
6	-0.674523000	-2.281164000	1.679628000
1	-0.681296000	-2.928291000	2.565243000
6	-0.696793000	-3.139154000	0.407047000
1	0.173265000	-3.807880000	0.385598000
1	-1.592371000	-3.773413000	0.390505000
6	-0.680442000	-2.216435000	-0.816362000
1	-0.688234000	-2.819479000	-1.734774000
6	0.586718000	-1.336601000	-0.815806000
1	1.473315000	-1.988168000	-0.812104000
6	0.627619000	-0.448594000	-2.070741000
1	0.611600000	-1.090955000	-2.961800000
6	1.901945000	0.401404000	-2.065185000
1	2.785901000	-0.249301000	-2.076704000
1	1.948331000	1.023477000	-2.968370000
6	1.916408000	1.289430000	-0.816930000
1	2.825450000	1.905960000	-0.812949000
6	1.892782000	0.435316000	0.472811000
1	2.780914000	-0.212570000	0.488606000
6	1.916408000	1.371241000	1.694938000
1	2.825706000	1.984854000	1.663929000

1	1.959382000	0.799004000	2.627270000
6	0.600037000	-1.419624000	1.685733000
1	1.488062000	-2.065082000	1.646720000
1	0.661439000	-0.867209000	2.626883000

**2**			
6	-0.078968000	-0.473428000	0.778513000
6	-0.916278000	-1.685513000	1.239372000
1	-0.409684000	-2.626745000	1.011107000
1	-1.019665000	-1.646266000	2.332290000
6	-2.306746000	-1.679646000	0.581125000
1	-2.867045000	-2.565312000	0.904830000
6	-3.056734000	-0.407053000	0.998709000
1	-3.189091000	-0.385412000	2.088042000
1	-4.058640000	-0.390676000	0.550928000
6	-2.254289000	0.816365000	0.542685000
1	-2.777186000	1.734853000	0.842943000
6	-0.851855000	0.815855000	1.185363000
1	-0.961176000	0.812185000	2.280172000
6	-0.066570000	2.070695000	0.768541000
1	-0.627452000	2.961825000	1.081883000
6	0.066569000	2.070695000	-0.768541000
1	0.627451000	2.961826000	-1.081882000
6	-1.313821000	2.065039000	-1.432659000
1	-1.872855000	2.968289000	-1.156090000
1	-1.203680000	2.076420000	-2.524751000
6	-2.085696000	0.816852000	-0.993015000
1	-3.079073000	0.812864000	-1.461685000
6	-1.338358000	-0.472869000	-1.407355000
1	-1.232967000	-0.488868000	-2.501617000
6	-2.155913000	-1.694950000	-0.951005000
1	-3.147009000	-1.663968000	-1.421191000
1	-1.685253000	-2.627287000	-1.279354000
6	0.078968000	-0.473428000	-0.778513000
6	0.916278000	-1.685512000	-1.239372000
1	1.019665000	-1.646265000	-2.332290000
1	0.409684000	-2.626744000	-1.011109000
6	2.306746000	-1.679645000	-0.581126000
1	2.867046000	-2.565311000	-0.904831000
6	2.155913000	-1.694950000	0.951005000
1	3.147010000	-1.663968000	1.421192000
1	1.685254000	-2.627287000	1.279353000
6	1.338359000	-0.472869000	1.407355000
1	1.232968000	-0.488869000	2.501617000
6	2.085695000	0.816852000	0.993016000
1	3.079072000	0.812865000	1.461685000
6	1.313820000	2.065039000	1.432659000
1	1.872854000	2.968289000	1.156090000
1	1.203679000	2.076421000	2.524751000
6	0.851855000	0.815856000	-1.185363000

1	0.961176000	0.812185000	-2.280172000
6	2.254288000	0.816365000	-0.542684000
1	2.777186000	1.734853000	-0.842942000
6	3.056734000	-0.407052000	-0.998709000
1	4.058640000	-0.390675000	-0.550927000
1	3.189092000	-0.385411000	-2.088041000

**M1**			
1	-3.505162000	2.712000000	-2.475472000
6	-2.684566000	1.732215000	0.757929000
6	-2.396031000	2.009358000	-0.755183000
6	-1.986099000	0.707849000	-1.474243000
1	-1.046176000	0.319842000	-1.076815000
1	-1.803228000	0.931151000	-2.534069000
6	-3.080014000	-0.365442000	-1.345062000
1	-2.746792000	-1.286228000	-1.839265000
6	-4.366441000	0.148815000	-2.006166000
1	-5.158468000	-0.607939000	-1.938996000
1	-4.193487000	0.342234000	-3.072602000
6	-4.807617000	1.438269000	-1.303370000
1	-5.724467000	1.820193000	-1.773110000
6	-3.711591000	2.518756000	-1.412181000
6	-4.172596000	3.827866000	-0.749002000
1	-5.092130000	4.172075000	-1.241758000
6	-3.080014000	4.893321000	-0.884242000
1	-2.885343000	5.097418000	-1.945083000
1	-3.410695000	5.836493000	-0.430448000
6	-1.800737000	4.402592000	-0.198387000
1	-1.017062000	5.166934000	-0.288205000
6	-1.292015000	3.093984000	-0.846771000
1	-1.075990000	3.280677000	-1.908442000
6	-0.005095000	2.645233000	-0.131648000
1	0.755091000	3.431596000	-0.221304000
1	0.416031000	1.754860000	-0.606764000
6	-0.301388000	2.376446000	1.354944000
1	0.610639000	2.030125000	1.856893000
6	-0.795329000	3.674536000	2.008650000
1	-0.996116000	3.509193000	3.074878000
1	-0.024088000	4.452455000	1.941501000
6	-2.073867000	4.135675000	1.298728000
1	-2.441317000	5.060961000	1.763417000
6	-3.173654000	3.059127000	1.407364000
1	-3.374718000	2.860410000	2.470664000
6	-4.471808000	3.539953000	0.736925000
1	-4.802108000	4.467408000	1.224354000
6	-5.556467000	2.466462000	0.872260000
1	-5.768196000	2.279919000	1.933063000
1	-6.491913000	2.811512000	0.413192000
6	-5.085063000	1.175980000	0.193774000
1	-5.863313000	0.406126000	0.283583000

6	-3.788104000	0.647378000	0.849655000
1	-3.982973000	0.439750000	1.911587000
6	-3.358626000	-0.650265000	0.141997000
1	-4.159697000	-1.394876000	0.231762000
1	-2.476082000	-1.085725000	0.619845000
6	-1.393626000	1.301371000	1.483010000
1	-1.623863000	1.127169000	2.542910000
1	-1.022284000	0.353175000	1.090808000
1	3.505162000	-2.712000000	-2.475472000
6	2.684566000	-1.732215000	0.757929000
6	2.396031000	-2.009358000	-0.755183000
6	1.986099000	-0.707849000	-1.474243000
1	1.046176000	-0.319842000	-1.076815000
1	1.803228000	-0.931151000	-2.534069000
6	3.080014000	0.365442000	-1.345062000
1	2.746792000	1.286228000	-1.839265000
6	4.366441000	-0.148815000	-2.006166000
1	5.158468000	0.607939000	-1.938996000
1	4.193487000	-0.342234000	-3.072602000
6	4.807617000	-1.438269000	-1.303370000
1	5.724467000	-1.820193000	-1.773110000
6	3.711591000	-2.518756000	-1.412181000
6	4.172596000	-3.827866000	-0.749002000
1	5.092130000	-4.172075000	-1.241758000
6	3.080014000	-4.893321000	-0.884242000
1	2.885343000	-5.097418000	-1.945083000
1	3.410695000	-5.836493000	-0.430448000
6	1.800737000	-4.402592000	-0.198387000
1	1.017062000	-5.166934000	-0.288205000
6	1.292015000	-3.093984000	-0.846771000
1	1.075990000	-3.280677000	-1.908442000
6	0.005095000	-2.645233000	-0.131648000
1	-0.755091000	-3.431596000	-0.221304000
1	-0.416031000	-1.754860000	-0.606764000
6	0.301388000	-2.376446000	1.354944000
1	-0.610639000	-2.030125000	1.856893000
6	0.795329000	-3.674536000	2.008650000
1	0.996116000	-3.509193000	3.074878000
1	0.024088000	-4.452455000	1.941501000
6	2.073867000	-4.135675000	1.298728000
1	2.441317000	-5.060961000	1.763417000
6	3.173654000	-3.059127000	1.407364000
1	3.374718000	-2.860410000	2.470664000
6	4.471808000	-3.539953000	0.736925000
1	4.802108000	-4.467408000	1.224354000
6	5.556467000	-2.466462000	0.872260000
1	5.768196000	-2.279919000	1.933063000
1	6.491913000	-2.811512000	0.413192000
6	5.085063000	-1.175980000	0.193774000
1	5.863313000	-0.406126000	0.283583000

6	3.788104000	-0.647378000	0.849655000
1	3.982973000	-0.439750000	1.911587000
6	3.358626000	0.650265000	0.141997000
1	4.159697000	1.394876000	0.231762000
1	2.476082000	1.085725000	0.619845000
6	1.393626000	-1.301371000	1.483010000
1	1.623863000	-1.127169000	2.542910000
1	1.022284000	-0.353175000	1.090808000

**M2**			
1	-5.570421000	-1.060211000	0.739069000
6	-2.571271000	0.241837000	-0.441859000
6	-4.121117000	0.223537000	-0.229148000
6	-4.853693000	0.129032000	-1.584338000
1	-4.667858000	1.014423000	-2.197639000
1	-5.935980000	0.102562000	-1.398164000
6	-4.424656000	-1.129588000	-2.358391000
1	-4.939006000	-1.154966000	-3.326846000
6	-4.793269000	-2.374407000	-1.539453000
1	-4.508756000	-3.285364000	-2.081512000
1	-5.878739000	-2.417192000	-1.382687000
6	-4.069523000	-2.315673000	-0.189994000
1	-4.331833000	-3.198337000	0.409355000
6	-4.480079000	-1.049947000	0.591862000
6	-3.793575000	-1.017296000	1.967996000
1	-4.078073000	-1.917946000	2.529005000
6	-4.228314000	0.233854000	2.737485000
1	-5.314290000	0.217064000	2.896683000
1	-3.757843000	0.252882000	3.729034000
6	-3.830367000	1.483360000	1.945424000
1	-4.134138000	2.382932000	2.497654000
6	-4.513793000	1.501231000	0.557286000
1	-5.604400000	1.501880000	0.696540000
6	-4.090457000	2.776579000	-0.194576000
1	-4.392038000	3.656929000	0.387068000
1	-4.601744000	2.851554000	-1.159747000
6	-2.563716000	2.782327000	-0.393121000
1	-2.267140000	3.678660000	-0.951596000
6	-1.880814000	2.775735000	0.981367000
1	-0.790852000	2.802499000	0.864346000
1	-2.164533000	3.669748000	1.551290000
6	-2.299326000	1.512038000	1.740497000
1	-1.807095000	1.490336000	2.722629000
6	-1.891948000	0.246552000	0.958348000
1	-0.804620000	0.257835000	0.805024000
6	-2.266918000	-1.019258000	1.747127000
1	-1.759506000	-0.993679000	2.721304000
6	-1.836309000	-2.266610000	0.969849000
1	-0.748086000	-2.268363000	0.837290000
1	-2.090934000	-3.172642000	1.534767000

6	-2.537903000	-2.286546000	-0.391885000
1	-2.232620000	-3.181423000	-0.951024000
6	-2.175921000	-1.035261000	-1.226706000
1	-1.090700000	-1.017696000	-1.395353000
6	-2.901543000	-1.108333000	-2.582019000
1	-2.591363000	-2.018971000	-3.110065000
1	-2.620333000	-0.266638000	-3.223132000
6	-2.140616000	1.528502000	-1.177509000
1	-1.050701000	1.514524000	-1.303739000
1	-2.565009000	1.569633000	-2.183810000
1	0.804621000	0.257836000	-0.805027000
6	4.121116000	0.223537000	0.229149000
6	2.571271000	0.241837000	0.441858000
6	2.140615000	1.528501000	1.177508000
1	2.565007000	1.569631000	2.183810000
1	1.050699000	1.514523000	1.303736000
6	2.563715000	2.782326000	0.393122000
1	2.267138000	3.678660000	0.951596000
6	1.880816000	2.775735000	-0.981368000
1	2.164535000	3.669748000	-1.551290000
1	0.790853000	2.802499000	-0.864348000
6	2.299328000	1.512038000	-1.740498000
1	1.807099000	1.490337000	-2.722631000
6	1.891949000	0.246552000	-0.958350000
6	2.266920000	-1.019257000	-1.747129000
1	1.759509000	-0.993678000	-2.721306000
6	1.836310000	-2.266610000	-0.969851000
1	0.748087000	-2.268363000	-0.837295000
1	2.090936000	-3.172641000	-1.534770000
6	2.537903000	-2.286547000	0.391883000
1	2.232619000	-3.181424000	0.951021000
6	2.175919000	-1.035262000	1.226704000
1	1.090698000	-1.017697000	1.395349000
6	2.901540000	-1.108334000	2.582018000
1	2.591358000	-2.018972000	3.110063000
1	2.620328000	-0.266640000	3.223131000
6	4.424653000	-1.129590000	2.358392000
1	4.939001000	-1.154967000	3.326848000
6	4.793267000	-2.374408000	1.539454000
1	5.878737000	-2.417193000	1.382689000
1	4.508753000	-3.285365000	2.081512000
6	4.069523000	-2.315673000	0.189994000
1	4.331833000	-3.198337000	-0.409355000
6	4.480079000	-1.049947000	-0.591861000
1	5.570421000	-1.060211000	-0.739067000
6	3.793578000	-1.017296000	-1.967996000
1	4.078076000	-1.917945000	-2.529004000
6	4.228318000	0.233855000	-2.737484000
1	5.314294000	0.217064000	-2.896679000
1	3.757848000	0.252883000	-3.729033000

6	3.830370000	1.483360000	-1.945423000
1	4.134142000	2.382933000	-2.497652000
6	4.513794000	1.501231000	-0.557284000
1	5.604401000	1.501880000	-0.696536000
6	4.090457000	2.776579000	0.194578000
1	4.392039000	3.656929000	-0.387065000
1	4.601742000	2.851554000	1.159750000
6	4.853691000	0.129031000	1.584341000
1	5.935978000	0.102561000	1.398167000
1	4.667855000	1.014422000	2.197642000

**M3**			
1	-3.282957000	-0.315562000	-2.491761000
6	-4.362175000	0.400655000	0.734990000
6	-4.451788000	0.424961000	-0.827203000
6	-4.389418000	1.875406000	-1.352004000
1	-5.251766000	2.456199000	-1.014693000
1	-4.439539000	1.854774000	-2.449159000
6	-3.094146000	2.572658000	-0.900022000
1	-3.091441000	3.607569000	-1.263372000
6	-1.887975000	1.815661000	-1.472916000
1	-0.953137000	2.306299000	-1.171743000
1	-1.918575000	1.825809000	-2.569953000
6	-1.916817000	0.372536000	-0.959159000
1	-1.064431000	-0.184154000	-1.369330000
6	-3.216287000	-0.334155000	-1.393668000
6	-3.215905000	-1.797390000	-0.920781000
1	-2.342199000	-2.308599000	-1.347711000
6	-4.501986000	-2.491451000	-1.379849000
1	-4.559517000	-2.485134000	-2.476007000
1	-4.501186000	-3.542565000	-1.063597000
6	-5.712951000	-1.766591000	-0.783380000
1	-6.637015000	-2.265835000	-1.104845000
6	-5.761632000	-0.291044000	-1.246395000
1	-5.837381000	-0.262976000	-2.342872000
6	-6.996352000	0.388948000	-0.627481000
1	-7.901926000	-0.139073000	-0.952213000
1	-7.094680000	1.420589000	-0.980607000
6	-6.896657000	0.353936000	0.908516000
1	-7.764943000	0.862062000	1.345560000
6	-6.858525000	-1.107787000	1.375661000
1	-6.804237000	-1.154319000	2.470884000
1	-7.777044000	-1.627982000	1.075113000
6	-5.635987000	-1.796351000	0.759353000
1	-5.593075000	-2.842367000	1.092810000
6	-4.336940000	-1.087097000	1.194478000
1	-4.274327000	-1.099718000	2.292880000
6	-3.107891000	-1.810220000	0.618315000
1	-3.109090000	-2.850554000	0.971203000
6	-1.827689000	-1.105838000	1.078105000

1	-1.758125000	-1.130755000	2.173420000
1	-0.944919000	-1.629094000	0.689302000
6	-1.839143000	0.343154000	0.583046000
1	-0.921866000	0.850429000	0.904351000
6	-3.051481000	1.114774000	1.153770000
1	-2.990328000	1.114001000	2.251530000
6	-3.009146000	2.564082000	0.637207000
1	-2.071619000	3.035479000	0.959070000
1	-3.820567000	3.159347000	1.068321000
6	-5.608175000	1.062513000	1.360823000
1	-5.519410000	1.015129000	2.454708000
1	-5.665491000	2.122821000	1.101686000
1	4.274337000	-1.099713000	-2.292882000
6	4.451785000	0.424959000	0.827205000
6	4.362179000	0.400657000	-0.734989000
6	5.608182000	1.062515000	-1.360814000
1	5.665499000	2.122822000	-1.101674000
1	5.519423000	1.015133000	-2.454699000
6	6.896662000	0.353935000	-0.908502000
1	7.764950000	0.862061000	-1.345541000
6	6.858530000	-1.107787000	-1.375651000
1	7.777048000	-1.627983000	-1.075100000
1	6.804248000	-1.154316000	-2.470874000
6	5.635989000	-1.796350000	-0.759351000
1	5.593077000	-2.842366000	-1.092810000
6	4.336945000	-1.087094000	-1.194480000
6	3.107892000	-1.810217000	-0.618324000
1	3.109092000	-2.850550000	-0.971214000
6	1.827693000	-1.105833000	-1.078119000
1	1.758134000	-1.130747000	-2.173434000
1	0.944921000	-1.629089000	-0.689321000
6	1.839146000	0.343158000	-0.583056000
1	0.921871000	0.850434000	-0.904365000
6	3.051488000	1.114778000	-1.153773000
1	2.990340000	1.114008000	-2.251533000
6	3.009152000	2.564085000	-0.637207000
1	2.071627000	3.035484000	-0.959073000
1	3.820575000	3.159350000	-1.068315000
6	3.094145000	2.572658000	0.900022000
1	3.091439000	3.607568000	1.263375000
6	1.887971000	1.815660000	1.472909000
1	1.918565000	1.825806000	2.569946000
1	0.953134000	2.306300000	1.171734000
6	1.916814000	0.372537000	0.959149000
1	1.064426000	-0.184153000	1.369315000
6	3.216280000	-0.334156000	1.393662000
1	3.282946000	-0.315566000	2.491756000
6	3.215899000	-1.797390000	0.920772000
1	2.342191000	-2.308599000	1.347698000
6	4.501977000	-2.491454000	1.379845000

1	4.559503000	-2.485139000	2.476003000
1	4.501177000	-3.542568000	1.063591000
6	5.712946000	-1.766594000	0.783383000
1	6.637008000	-2.265840000	1.104852000
6	5.761626000	-0.291048000	1.246402000
1	5.837370000	-0.262982000	2.342880000
6	6.996350000	0.388944000	0.627495000
1	7.901921000	-0.139078000	0.952230000
1	7.094678000	1.420584000	0.980624000
6	4.389414000	1.875403000	1.352009000
1	4.439530000	1.854769000	2.449164000
1	5.251764000	2.456196000	1.014703000

**MP1**			
1	-5.174164000	0.373296000	2.354681000
6	-3.597898000	0.214856000	-0.740813000
6	-3.723803000	-0.031440000	0.799883000
6	-2.610485000	0.716548000	1.563343000
1	-1.622239000	0.330839000	1.301736000
1	-2.738861000	0.533420000	2.638762000
6	-2.660412000	2.226512000	1.277341000
1	-1.841028000	2.725479000	1.809096000
6	-4.008963000	2.783636000	1.754182000
1	-4.060892000	3.864642000	1.571544000
1	-4.120214000	2.632605000	2.835608000
6	-5.137964000	2.066936000	1.005117000
1	-6.109666000	2.451726000	1.344362000
6	-5.089474000	0.548289000	1.271579000
6	-6.252169000	-0.164420000	0.560603000
1	-7.201309000	0.252714000	0.924220000
6	-6.198599000	-1.667301000	0.853203000
1	-6.298530000	-1.841807000	1.932314000
1	-7.037703000	-2.179961000	0.365273000
6	-4.870295000	-2.239192000	0.347165000
1	-4.831740000	-3.318121000	0.549515000
6	-3.671044000	-1.560262000	1.049828000
1	-3.743715000	-1.737907000	2.132444000
6	-2.362520000	-2.169703000	0.515470000
1	-2.353542000	-3.248108000	0.718515000
1	-1.495306000	-1.749136000	1.034562000
6	-2.253583000	-1.924929000	-1.000444000
1	-1.308461000	-2.337921000	-1.372355000
6	-3.435440000	-2.605918000	-1.704917000
1	-3.366076000	-2.456885000	-2.790083000
1	-3.414218000	-3.688474000	-1.525304000
6	-4.743856000	-2.008584000	-1.175273000
1	-5.597807000	-2.482852000	-1.678415000
6	-4.796423000	-0.489939000	-1.441723000
1	-4.704392000	-0.314335000	-2.524014000
6	-6.129801000	0.100840000	-0.954040000

1	-6.955141000	-0.402630000	-1.476038000
6	-6.170033000	1.604357000	-1.245722000
1	-6.093776000	1.777740000	-2.326958000
1	-7.127638000	2.029610000	-0.918419000
6	-5.013641000	2.297379000	-0.517289000
1	-5.044351000	3.376749000	-0.718756000
6	-3.648518000	1.743831000	-0.990031000
1	-3.544623000	1.923048000	-2.069908000
6	-2.520261000	2.472628000	-0.236311000
1	-2.582146000	3.548531000	-0.443538000
1	-1.536348000	2.144501000	-0.586569000
6	-2.299913000	-0.414857000	-1.286144000
1	-2.250852000	-0.238164000	-2.369457000
1	-1.420281000	0.065277000	-0.856109000
6	4.014125000	-0.415497000	-0.826913000
6	2.826690000	-0.528286000	-1.806344000
1	2.744597000	0.362537000	-2.434385000
1	3.009463000	-1.371201000	-2.486542000
6	1.503722000	-0.744393000	-1.050417000
1	0.677898000	-0.790615000	-1.769323000
6	1.584616000	-2.057690000	-0.260376000
1	1.734694000	-2.902133000	-0.945378000
1	0.645349000	-2.240379000	0.275177000
6	2.748760000	-1.970843000	0.731702000
1	2.821910000	-2.908562000	1.299551000
6	4.080717000	-1.740189000	-0.011551000
1	4.240191000	-2.565786000	-0.721508000
6	5.254074000	-1.705129000	0.981765000
1	5.284912000	-2.656922000	1.529631000
6	5.010010000	-0.553839000	1.979677000
1	5.841834000	-0.515369000	2.696209000
6	3.688535000	-0.755310000	2.728003000
1	3.718049000	-1.686514000	3.308319000
1	3.534085000	0.064242000	3.441757000
6	2.535351000	-0.803160000	1.721157000
1	1.585740000	-0.953883000	2.252721000
6	2.447274000	0.515039000	0.916815000
1	2.274144000	1.348150000	1.613222000
6	1.271445000	0.418208000	-0.070567000
1	0.347762000	0.249830000	0.495941000
1	1.133595000	1.357985000	-0.615311000
6	3.787593000	0.762199000	0.178529000
6	3.799337000	2.117094000	-0.561151000
1	3.651909000	2.920002000	0.173848000
1	2.968733000	2.189450000	-1.267857000
6	5.127676000	2.327491000	-1.307852000
1	5.096653000	3.283352000	-1.845057000
6	5.348523000	1.178854000	-2.308844000
1	6.308849000	1.313868000	-2.822470000
1	4.576352000	1.194193000	-3.084762000

6	5.356064000	-0.168696000	-1.563824000
1	5.512943000	-0.985556000	-2.282824000
6	6.514956000	-0.171123000	-0.538821000
1	7.459858000	-0.015944000	-1.076981000
6	6.569844000	-1.497825000	0.225370000
1	7.414652000	-1.492179000	0.926133000
1	6.735407000	-2.329450000	-0.471741000
6	4.969205000	0.766874000	1.192566000
1	4.805828000	1.596171000	1.897170000
6	6.301239000	0.996987000	0.449309000
1	7.117872000	0.994554000	1.184419000
6	6.279655000	2.337305000	-0.293484000
1	7.236629000	2.501287000	-0.805216000
1	6.149211000	3.160974000	0.420030000

**MP2**			
1	4.843389000	0.685898000	-2.203516000
6	3.084796000	-0.044669000	0.704943000
6	3.269425000	0.088563000	-0.843205000
6	2.292110000	1.135869000	-1.417347000
1	1.254310000	0.821326000	-1.289319000
1	2.459110000	1.216705000	-2.500050000
6	2.499326000	2.507638000	-0.752591000
1	1.773629000	3.222887000	-1.159444000
6	3.927035000	2.994236000	-1.037834000
1	4.091469000	3.980225000	-0.584727000
1	4.080913000	3.105222000	-2.118839000
6	4.923077000	1.978287000	-0.467092000
1	5.949683000	2.311858000	-0.671939000
6	4.717019000	0.593055000	-1.114509000
6	5.748634000	-0.413697000	-0.578177000
1	6.758088000	-0.039699000	-0.797207000
6	5.542720000	-1.776595000	-1.246670000
1	5.683748000	-1.686584000	-2.331521000
1	6.289113000	-2.494838000	-0.883507000
6	4.131510000	-2.287368000	-0.938865000
1	3.982366000	-3.267693000	-1.411259000
6	3.059436000	-1.310848000	-1.477270000
1	3.174149000	-1.219033000	-2.566791000
6	1.662661000	-1.869723000	-1.152350000
1	1.546188000	-2.855019000	-1.621163000
1	0.879889000	-1.231819000	-1.574273000
6	1.494118000	-1.997384000	0.372494000
1	0.490206000	-2.371560000	0.602897000
6	2.547912000	-2.973458000	0.913105000
1	2.433107000	-3.091238000	1.998250000
1	2.413197000	-3.965799000	0.464095000
6	3.944055000	-2.431060000	0.587912000
1	4.708020000	-3.118326000	0.976669000
6	4.152093000	-1.046168000	1.235120000

1	4.018291000	-1.137145000	2.323420000
6	5.570147000	-0.523479000	0.950259000
1	6.300539000	-1.241288000	1.347917000
6	5.765946000	0.841382000	1.617731000
1	5.647915000	0.746804000	2.704960000
1	6.783182000	1.211325000	1.435414000
6	4.740181000	1.833365000	1.060211000
1	4.881553000	2.815102000	1.532026000
6	3.296304000	1.353939000	1.339676000
1	3.151929000	1.267333000	2.426169000
6	2.305580000	2.384534000	0.769722000
1	2.481609000	3.358048000	1.244647000
1	1.273801000	2.108023000	1.004071000
6	1.692943000	-0.622923000	1.033604000
1	1.594527000	-0.713900000	2.123953000
1	0.902198000	0.056280000	0.708822000
6	-3.084797000	0.044670000	-0.704944000
6	-1.692944000	0.622924000	-1.033606000
1	-0.902197000	-0.056278000	-0.708825000
1	-1.594529000	0.713901000	-2.123955000
6	-1.494119000	1.997385000	-0.372496000
1	-0.490208000	2.371562000	-0.602901000
6	-2.547914000	2.973459000	-0.913106000
1	-2.433110000	3.091239000	-1.998252000
1	-2.413199000	3.965800000	-0.464097000
6	-3.944056000	2.431060000	-0.587912000
1	-4.708023000	3.118325000	-0.976668000
6	-4.152094000	1.046168000	-1.235120000
1	-4.018293000	1.137145000	-2.323419000
6	-5.570147000	0.523478000	-0.950257000
1	-6.300541000	1.241287000	-1.347914000
6	-5.748634000	0.413696000	0.578179000
1	-6.758088000	0.039697000	0.797210000
6	-5.542720000	1.776594000	1.246672000
1	-6.289113000	2.494837000	0.883510000
1	-5.683747000	1.686583000	2.331523000
6	-4.131510000	2.287368000	0.938865000
1	-3.982366000	3.267693000	1.411259000
6	-3.059435000	1.310849000	1.477269000
1	-3.174146000	1.219033000	2.566790000
6	-1.662660000	1.869724000	1.152348000
1	-1.546188000	2.855020000	1.621160000
1	-0.879888000	1.231821000	1.574271000
6	-3.269424000	-0.088563000	0.843205000
6	-2.292108000	-1.135868000	1.417346000
1	-2.459107000	-1.216704000	2.500049000
1	-1.254309000	-0.821325000	1.289318000
6	-2.499324000	-2.507637000	0.752590000
1	-1.773627000	-3.222886000	1.159443000
6	-2.305579000	-2.384533000	-0.769723000

1	-2.481609000	-3.358048000	-1.244648000
1	-1.273801000	-2.108022000	-1.004073000
6	-3.296304000	-1.353939000	-1.339677000
1	-3.151931000	-1.267333000	-2.426170000
6	-4.740181000	-1.833365000	-1.060210000
1	-4.881553000	-2.815103000	-1.532024000
6	-5.765947000	-0.841383000	-1.617729000
1	-6.783183000	-1.211326000	-1.435411000
1	-5.647917000	-0.746805000	-2.704958000
6	-4.717018000	-0.593055000	1.114510000
1	-4.843386000	-0.685898000	2.203517000
6	-4.923076000	-1.978287000	0.467093000
1	-5.949681000	-2.311858000	0.671941000
6	-3.927032000	-2.994237000	1.037835000
1	-4.091467000	-3.980225000	0.584728000
1	-4.080910000	-3.105223000	2.118839000
**MP3**			
1	-5.268045000	1.264183000	-1.463080000
6	-2.814791000	-0.073369000	0.606926000
6	-4.283252000	0.271218000	0.189379000
6	-4.933450000	1.216760000	1.221733000
1	-5.028030000	0.733209000	2.197366000
1	-5.954740000	1.449687000	0.890987000
6	-4.122266000	2.515666000	1.370089000
1	-4.591423000	3.153774000	2.128917000
6	-4.090978000	3.247187000	0.020908000
1	-3.528490000	4.185515000	0.108999000
1	-5.109846000	3.509100000	-0.292056000
6	-3.437992000	2.336621000	-1.024384000
1	-3.416580000	2.845959000	-1.997664000
6	-4.234267000	1.023128000	-1.173595000
6	-3.609487000	0.131391000	-2.260096000
1	-3.603091000	0.680903000	-3.211339000
6	-4.424367000	-1.156949000	-2.409335000
1	-5.451821000	-0.917327000	-2.712436000
1	-3.995292000	-1.788858000	-3.197665000
6	-4.430352000	-1.913676000	-1.077171000
1	-5.008276000	-2.841830000	-1.182115000
6	-5.067879000	-1.058451000	0.043308000
1	-6.104807000	-0.820988000	-0.234504000
6	-5.058081000	-1.864940000	1.354716000
1	-5.626992000	-2.792696000	1.213542000
1	-5.556588000	-1.313992000	2.158826000
6	-3.608396000	-2.198351000	1.751589000
1	-3.603498000	-2.749332000	2.700014000
6	-2.968428000	-3.053516000	0.648994000
1	-1.936275000	-3.310779000	0.920383000
1	-3.516908000	-3.996829000	0.531256000
6	-2.983520000	-2.265203000	-0.665113000

1	-2.522152000	-2.865021000	-1.461712000
6	-2.188883000	-0.952386000	-0.515963000
1	-1.158384000	-1.195666000	-0.222747000
6	-2.157290000	-0.185748000	-1.848702000
1	-1.694524000	-0.823712000	-2.614237000
6	-1.347345000	1.103471000	-1.689136000
1	-0.316141000	0.861971000	-1.410184000
1	-1.303318000	1.646641000	-2.642122000
6	-1.991244000	1.983256000	-0.613897000
1	-1.412519000	2.910333000	-0.501135000
6	-2.029914000	1.256238000	0.751268000
1	-1.002277000	1.023697000	1.061491000
6	-2.680301000	2.182433000	1.794373000
1	-2.093855000	3.106704000	1.872838000
1	-2.669953000	1.722714000	2.787878000
6	-2.803572000	-0.897704000	1.912516000
1	-1.764352000	-1.133093000	2.175460000
1	-3.208155000	-0.319884000	2.747377000
6	2.814788000	0.073380000	-0.606920000
6	2.803557000	0.897748000	-1.912490000
1	3.208142000	0.319953000	-2.747366000
1	1.764335000	1.133136000	-2.175424000
6	3.608372000	2.198398000	-1.751533000
1	3.603465000	2.749404000	-2.699944000
6	2.968400000	3.053530000	-0.648914000
1	1.936244000	3.310791000	-0.920293000
1	3.516872000	3.996845000	-0.531153000
6	2.983503000	2.265184000	0.665173000
1	2.522133000	2.864979000	1.461789000
6	2.188876000	0.952364000	0.515994000
1	1.158374000	1.195642000	0.222788000
6	2.157294000	0.185693000	1.848713000
1	1.694527000	0.823634000	2.614266000
6	3.609496000	-0.131445000	2.260094000
1	3.603107000	-0.680981000	3.211323000
6	4.424366000	1.156898000	2.409363000
1	3.995288000	1.788784000	3.197710000
1	5.451822000	0.917277000	2.712454000
6	4.430340000	1.913658000	1.077218000
1	5.008256000	2.841815000	1.182183000
6	5.067869000	1.058467000	-0.043285000
1	6.104801000	0.821005000	0.234517000
6	5.058060000	1.864989000	-1.354673000
1	5.626964000	2.792746000	-1.213476000
1	5.556569000	1.314066000	-2.158798000
6	4.283252000	-0.271205000	-0.189387000
6	4.933454000	-1.216715000	-1.221767000
1	5.954748000	-1.449643000	-0.891030000
1	5.028027000	-0.733140000	-2.197388000
6	4.122280000	-2.515624000	-1.370153000

1	4.591441000	-3.153710000	-2.128999000
6	2.680311000	-2.182392000	-1.794423000
1	2.093873000	-3.106666000	-1.872910000
1	2.669956000	-1.722649000	-2.787917000
6	2.029921000	-1.256229000	-0.751293000
1	1.002281000	-1.023688000	-1.061508000
6	1.991261000	-1.983282000	0.613854000
1	1.412543000	-2.910360000	0.501071000
6	1.347359000	-1.103529000	1.689117000
1	1.303340000	-1.646723000	2.642090000
1	0.316152000	-0.862030000	1.410177000
6	4.234279000	-1.023149000	1.173568000
1	5.268060000	-1.264204000	1.463043000
6	3.438013000	-2.336645000	1.024327000
1	3.416609000	-2.846007000	1.997594000
6	4.091003000	-3.247179000	-0.020990000
1	3.528523000	-4.185510000	-0.109103000
1	5.109874000	-3.509092000	0.291964000

**MP4**			
1	5.685163000	-0.197638000	-0.987220000
6	2.557916000	0.047378000	0.511370000
6	4.097595000	0.300010000	0.397953000
6	4.843276000	-0.354028000	1.580535000
1	4.556123000	0.102785000	2.531054000
1	5.919970000	-0.171468000	1.461741000
6	4.571752000	-1.867475000	1.636662000
1	5.090400000	-2.298978000	2.501480000
6	5.082545000	-2.518839000	0.343975000
1	4.911781000	-3.602700000	0.369928000
1	6.164519000	-2.364298000	0.243599000
6	4.350045000	-1.897675000	-0.850154000
1	4.712809000	-2.350511000	-1.783142000
6	4.602215000	-0.376435000	-0.910747000
6	3.909456000	0.238588000	-2.138767000
1	4.296361000	-0.246271000	-3.045455000
6	4.188320000	1.743715000	-2.194449000
1	5.267789000	1.921318000	-2.284803000
1	3.714093000	2.185684000	-3.080258000
6	3.648144000	2.409363000	-0.924869000
1	3.839634000	3.490204000	-0.964385000
6	4.330763000	1.832156000	0.338219000
1	5.412782000	2.016675000	0.275061000
6	3.760611000	2.536620000	1.583196000
1	3.951645000	3.614673000	1.508496000
1	4.263687000	2.193909000	2.493150000
6	2.245076000	2.282223000	1.678762000
1	1.845878000	2.763121000	2.580225000
6	1.561725000	2.862859000	0.432963000
1	0.476948000	2.712565000	0.488376000

1	1.733429000	3.945318000	0.374962000
6	2.125846000	2.171854000	-0.812745000
1	1.634965000	2.570783000	-1.711263000
6	1.875833000	0.651138000	-0.750784000
1	0.795420000	0.473206000	-0.667217000
6	2.395013000	-0.033034000	-2.026332000
1	1.883288000	0.401841000	-2.895890000
6	2.120813000	-1.538632000	-1.961809000
1	1.041522000	-1.722018000	-1.903000000
1	2.478730000	-2.028899000	-2.876356000
6	2.827224000	-2.133986000	-0.739977000
1	2.634378000	-3.214345000	-0.692407000
6	2.322014000	-1.483830000	0.569801000
1	1.243206000	-1.666194000	0.668089000
6	3.058166000	-2.119841000	1.762542000
1	2.861840000	-3.199465000	1.775224000
1	2.681807000	-1.724559000	2.711607000
6	1.979987000	0.768472000	1.747341000
1	0.900612000	0.577618000	1.793988000
1	2.401920000	0.367795000	2.672513000
6	-4.097595000	-0.300009000	-0.397954000
6	-4.843276000	0.354031000	-1.580535000
1	-4.556122000	-0.102782000	-2.531055000
1	-5.919969000	0.171470000	-1.461741000
6	-4.571751000	1.867477000	-1.636659000
1	-5.090400000	2.298982000	-2.501476000
6	-5.082546000	2.518839000	-0.343972000
1	-6.164519000	2.364297000	-0.243595000
1	-4.911782000	3.602700000	-0.369923000
6	-4.350046000	1.897673000	0.850157000
1	-4.712809000	2.350508000	1.783146000
6	-4.602216000	0.376434000	0.910748000
1	-5.685163000	0.197636000	0.987220000
6	-3.909457000	-0.238592000	2.138767000
1	-4.296362000	0.246267000	3.045455000
6	-2.395014000	0.033031000	2.026333000
1	-1.883289000	-0.401845000	2.895890000
6	-2.120813000	1.538629000	1.961812000
1	-2.478731000	2.028895000	2.876360000
1	-1.041522000	1.722016000	1.903003000
6	-2.827225000	2.133985000	0.739981000
1	-2.634379000	3.214344000	0.692413000
6	-2.322014000	1.483831000	-0.569799000
1	-1.243207000	1.666195000	-0.668086000
6	-3.058166000	2.119844000	-1.762539000
1	-2.861840000	3.199468000	-1.775218000
1	-2.681807000	1.724564000	-2.711603000
6	-2.557916000	-0.047377000	-0.511370000
6	-1.979986000	-0.768469000	-1.747341000
1	-0.900611000	-0.577616000	-1.793988000

1	-2.401918000	-0.367791000	-2.672513000
6	-2.245076000	-2.282220000	-1.678765000
1	-1.845877000	-2.763117000	-2.580228000
6	-3.760611000	-2.536618000	-1.583200000
1	-3.951644000	-3.614671000	-1.508502000
1	-4.263686000	-2.193905000	-2.493153000
6	-4.330762000	-1.832156000	-0.338222000
1	-5.412782000	-2.016675000	-0.275065000
6	-3.648144000	-2.409364000	0.924866000
1	-3.839634000	-3.490205000	0.964379000
6	-4.188320000	-1.743719000	2.194446000
1	-3.714094000	-2.185689000	3.080255000
1	-5.267789000	-1.921321000	2.284800000
6	-1.875833000	-0.651139000	0.750784000
1	-0.795420000	-0.473206000	0.667217000
6	-2.125846000	-2.171855000	0.812743000
1	-1.634965000	-2.570785000	1.711260000
6	-1.561725000	-2.862859000	-0.432967000
1	-1.733429000	-3.945318000	-0.374968000
1	-0.476948000	-2.712563000	-0.488379000

**MP5**			
1	-4.786364000	-0.867724000	2.290596000
6	-3.903815000	0.486814000	-0.787674000
6	-4.303462000	0.546503000	0.724365000
6	-5.643247000	1.292140000	0.901771000
1	-5.558136000	2.337659000	0.594333000
1	-5.901140000	1.303018000	1.969493000
6	-6.765468000	0.611735000	0.098671000
1	-7.696633000	1.178194000	0.221727000
6	-6.955531000	-0.822047000	0.613119000
1	-7.763127000	-1.320745000	0.062039000
1	-7.245984000	-0.809615000	1.671457000
6	-5.642465000	-1.592687000	0.437956000
1	-5.763393000	-2.619224000	0.810472000
6	-4.504928000	-0.913284000	1.227931000
6	-3.200242000	-1.716351000	1.093687000
1	-3.368806000	-2.734617000	1.469888000
6	-2.088806000	-1.040950000	1.902377000
1	-2.364493000	-1.008527000	2.964304000
1	-1.160141000	-1.620330000	1.828838000
6	-1.864591000	0.378743000	1.372299000
1	-1.060418000	0.863258000	1.942685000
6	-3.149400000	1.230417000	1.503206000
1	-3.433117000	1.289311000	2.563709000
6	-2.870017000	2.645511000	0.965425000
1	-2.053534000	3.097785000	1.542676000
1	-3.741311000	3.295104000	1.097197000
6	-2.475239000	2.570829000	-0.520967000
1	-2.301521000	3.582580000	-0.907171000

6	-1.194820000	1.735535000	-0.655195000
1	-0.882432000	1.684715000	-1.706294000
1	-0.376662000	2.205819000	-0.096923000
6	-1.461881000	0.326416000	-0.117505000
1	-0.559319000	-0.287926000	-0.218409000
6	-2.597740000	-0.351324000	-0.908381000
1	-2.317256000	-0.390229000	-1.971600000
6	-2.824514000	-1.784891000	-0.401238000
1	-1.890735000	-2.353474000	-0.513047000
6	-3.941184000	-2.451179000	-1.210720000
1	-3.656080000	-2.502851000	-2.269472000
1	-4.096303000	-3.482426000	-0.868285000
6	-5.235529000	-1.646572000	-1.051413000
1	-6.040589000	-2.125729000	-1.624783000
6	-5.056570000	-0.198426000	-1.565764000
1	-4.786690000	-0.228267000	-2.631156000
6	-6.382377000	0.564273000	-1.391708000
1	-7.170730000	0.055669000	-1.961005000
1	-6.308949000	1.577488000	-1.799902000
6	-3.602639000	1.901866000	-1.325711000
1	-3.311394000	1.822012000	-2.381868000
1	-4.493918000	2.533890000	-1.298235000
6	3.404514000	0.063391000	0.711763000
6	2.310399000	1.079214000	1.101701000
1	1.318774000	0.718743000	0.821404000
1	2.303252000	1.187024000	2.194987000
6	2.565337000	2.445388000	0.442689000
1	1.758078000	3.137105000	0.714380000
6	3.911942000	2.996806000	0.931580000
1	3.892987000	3.134350000	2.020332000
1	4.106324000	3.980148000	0.484619000
6	5.022781000	2.012868000	0.546180000
1	5.991697000	2.393335000	0.897830000
6	4.772556000	0.633279000	1.189122000
1	4.725540000	0.752016000	2.281964000
6	5.912208000	-0.340030000	0.843394000
1	6.860789000	0.079872000	1.205015000
6	5.976090000	-0.487457000	-0.690918000
1	6.786171000	-1.181739000	-0.952943000
6	6.220310000	0.871251000	-1.355042000
1	7.182586000	1.286229000	-1.028368000
1	6.274925000	0.751010000	-2.444755000
6	5.083948000	1.830598000	-0.986874000
1	5.259220000	2.807983000	-1.456384000
6	3.720101000	1.286092000	-1.472443000
1	3.748901000	1.172889000	-2.565685000
6	2.614615000	2.285104000	-1.087805000
1	2.824789000	3.256027000	-1.554159000
1	1.642399000	1.961174000	-1.469422000
6	3.466324000	-0.107604000	-0.842868000

6	2.166610000	-0.749999000	-1.370318000
1	2.252597000	-0.876097000	-2.458181000
1	1.308602000	-0.095753000	-1.204489000
6	1.915265000	-2.114638000	-0.706143000
1	0.969425000	-2.531175000	-1.075213000
6	1.844461000	-1.949917000	0.822941000
1	1.693506000	-2.929927000	1.293422000
1	0.984049000	-1.336109000	1.103820000
6	3.153058000	-1.329750000	1.342783000
1	3.095140000	-1.211146000	2.434267000
6	4.330972000	-2.271759000	1.000412000
1	4.148072000	-3.247780000	1.469879000
6	5.657968000	-1.697070000	1.506839000
1	6.478023000	-2.390612000	1.280126000
1	5.627138000	-1.580741000	2.597916000
6	4.639668000	-1.074440000	-1.176220000
1	4.679215000	-1.192528000	-2.269389000
6	4.384937000	-2.453368000	-0.532899000
1	5.223613000	-3.117668000	-0.782871000
6	3.075204000	-3.059295000	-1.052322000
1	2.911093000	-4.046347000	-0.601367000
1	3.133451000	-3.205513000	-2.138499000

**MP6**			
1	3.255902000	-1.305965000	2.225091000
6	3.939865000	0.728966000	-0.510665000
6	4.151718000	0.191381000	0.943740000
6	3.902963000	1.310637000	1.977375000
1	4.630764000	2.118814000	1.868595000
1	4.047048000	0.897347000	2.984898000
6	2.480006000	1.880951000	1.844159000
1	2.342161000	2.690967000	2.570718000
6	1.463059000	0.762753000	2.111214000
1	0.439702000	1.151269000	2.042228000
1	1.589177000	0.370212000	3.128360000
6	1.676490000	-0.357205000	1.087902000
1	0.957858000	-1.167378000	1.270445000
6	3.103425000	-0.930906000	1.202081000
6	3.298368000	-2.091808000	0.212809000
1	2.555641000	-2.871857000	0.429441000
6	4.711928000	-2.664230000	0.355288000
1	4.857820000	-3.051787000	1.371860000
1	4.852550000	-3.507013000	-0.333758000
6	5.738323000	-1.566889000	0.056165000
1	6.753486000	-1.975553000	0.150463000
6	5.588845000	-0.382667000	1.040726000
1	5.748764000	-0.747642000	2.065419000
6	6.644449000	0.686228000	0.704409000
1	7.646103000	0.246535000	0.791682000
1	6.605372000	1.514914000	1.418789000

6	6.426290000	1.202326000	-0.729638000
1	7.163888000	1.981744000	-0.956698000
6	6.583083000	0.034084000	-1.713010000
1	6.447469000	0.385622000	-2.743859000
1	7.594137000	-0.387617000	-1.645697000
6	5.540443000	-1.039392000	-1.382466000
1	5.637669000	-1.878487000	-2.085040000
6	4.113517000	-0.463929000	-1.496800000
1	3.963323000	-0.082146000	-2.517707000
6	3.067356000	-1.555844000	-1.215413000
1	3.204997000	-2.374651000	-1.934663000
6	1.656869000	-0.974189000	-1.352537000
1	1.497022000	-0.610381000	-2.375764000
1	0.906072000	-1.752865000	-1.166386000
6	1.481328000	0.170146000	-0.350790000
1	0.473811000	0.591204000	-0.447423000
6	2.502827000	1.301074000	-0.607773000
1	2.351802000	1.694151000	-1.623395000
6	2.270522000	2.424524000	0.418802000
1	1.246604000	2.805145000	0.313394000
1	2.937906000	3.271949000	0.231481000
6	5.008840000	1.785648000	-0.860772000
1	4.839432000	2.129321000	-1.890279000
1	4.917204000	2.667932000	-0.222094000
6	-3.322529000	0.027158000	0.633697000
6	-2.046472000	-0.712604000	1.083692000
1	-1.155728000	-0.255685000	0.652461000
1	-1.946857000	-0.616294000	2.173512000
6	-2.099169000	-2.198337000	0.690962000
1	-1.167236000	-2.689367000	0.998382000
6	-3.295184000	-2.860647000	1.389099000
1	-3.182147000	-2.794106000	2.478785000
1	-3.344182000	-3.926858000	1.133414000
6	-4.582915000	-2.153157000	0.950607000
1	-5.446246000	-2.613952000	1.450106000
6	-4.536534000	-0.657481000	1.326497000
1	-4.399057000	-0.566936000	2.414385000
6	-5.847791000	0.042030000	0.930923000
1	-6.683947000	-0.451036000	1.445343000
6	-6.034441000	-0.103697000	-0.593473000
1	-6.968202000	0.392476000	-0.891464000
6	-6.080077000	-1.581718000	-0.994245000
1	-6.932101000	-2.079702000	-0.513675000
1	-6.224743000	-1.671255000	-2.078672000
6	-4.772605000	-2.264008000	-0.578599000
1	-4.804972000	-3.325576000	-0.858806000
6	-3.558391000	-1.605478000	-1.274814000
1	-3.676603000	-1.698952000	-2.363876000
6	-2.272231000	-2.326901000	-0.833136000
1	-2.336775000	-3.386693000	-1.110625000

1	-1.398095000	-1.922402000	-1.352807000
6	-3.511779000	-0.098199000	-0.914835000
6	-2.380536000	0.637863000	-1.662685000
1	-2.555847000	0.546180000	-2.743220000
1	-1.411036000	0.173787000	-1.469078000
6	-2.331014000	2.123469000	-1.266369000
1	-1.499209000	2.612541000	-1.788281000
6	-2.132998000	2.252844000	0.254768000
1	-2.127004000	3.313062000	0.538249000
1	-1.160300000	1.848793000	0.549232000
6	-3.275421000	1.534204000	0.994253000
1	-3.126231000	1.627069000	2.079591000
6	-4.619321000	2.197644000	0.611550000
1	-4.579183000	3.259151000	0.890949000
6	-5.790360000	1.519925000	1.330390000
1	-6.731377000	2.021160000	1.069331000
1	-5.668926000	1.609346000	2.417672000
6	-4.854974000	0.591370000	-1.294173000
1	-4.984956000	0.501113000	-2.382988000
6	-4.804225000	2.086972000	-0.918442000
1	-5.761077000	2.550743000	-1.195076000
6	-3.658174000	2.790246000	-1.655129000
1	-3.639871000	3.856402000	-1.395189000
1	-3.812029000	2.724264000	-2.739827000
