# Supplementary material for: Assigning the absolute configuration of single aliphatic molecules by visual inspection
Source: Nat Commun. 2018 Jun 20;9:2420. doi: 10.1038/s41467-018-04843-z (PMC6010418; doi:10.1038/s41467-018-04843-z)
Supplement: Supplementary file 4 — Supplementary Data 2 [file 41467_2018_4843_MOESM4_ESM.pdf]

Supplementary Data 2

Assigning the absolute configuration of single aliphatic molecules by visual inspection

Daniel Ebeling,[1,‡,] Marina Šekutor,[2,‡,*] Marvin Stiefermann,[1] Jalmar Tschakert,[1] Jeremy E. P. Dahl,[3] Robert M. K. Carlson,[3] André Schirmeisen,[1,*] and Peter R. Schreiner[2,*]*

[1]Institute of Applied Physics, Justus-Liebig University, Heinrich-Buff-Ring 16, 35392 Giessen, Germany, Daniel.Ebeling@ap.physik.uni-giessen.de, Andre.Schirmeisen@ap.physik.uni-giessen.de

[2]Institute of Organic Chemistry, Justus-Liebig University, Heinrich-Buff-Ring 17, 35392 Giessen, Germany, Marina.Sekutor@org.Chemie.uni-giessen.de, prs@uni-giessen.de

[3]Stanford Institute for Materials and Energy Sciences, Stanford, CA 94305, USA

[‡]Both contributors are considered first authors.

Supplementary Data Set 2. Geometries in Cartesian coordinates in Å computed at the M06-2X/6-31G(d,p) level of theory.

**1**			
1	1.341271000	2.079023000	0.808134000
6	-0.596200000	-0.503725000	-0.472795000
6	0.596200000	0.503725000	-0.472795000
6	0.502701000	1.452063000	-1.684160000
1	0.596509000	0.903670000	-2.626419000
1	1.347472000	2.154559000	-1.645134000
6	-0.823208000	2.227657000	-1.676570000
1	-0.873227000	2.872673000	-2.561622000
6	-0.900804000	3.081236000	-0.406656000
1	-1.837427000	3.652991000	-0.389986000
1	-0.075438000	3.804262000	-0.386194000
6	-0.823208000	2.163431000	0.815127000
1	-0.870831000	2.763382000	1.735016000
6	0.497211000	1.370432000	0.813469000
6	0.595395000	0.488413000	2.067068000
1	0.535510000	1.129182000	2.958079000
6	1.920697000	-0.275593000	2.062180000
1	2.759793000	0.431896000	2.072671000
1	2.005141000	-0.894734000	2.964540000
6	1.993226000	-1.158905000	0.815163000
1	2.940953000	-1.715359000	0.809725000
6	1.913944000	-0.308319000	-0.471315000
1	2.757086000	0.399034000	-0.485051000
6	2.000798000	-1.240135000	-1.690751000
1	2.947150000	-1.794789000	-1.653800000
1	2.009431000	-0.668098000	-2.624527000
6	0.823208000	-2.227657000	-1.676570000
1	0.873227000	-2.872673000	-2.561622000
6	0.900804000	-3.081236000	-0.406656000
1	0.075438000	-3.804262000	-0.386194000
1	1.837427000	-3.652991000	-0.389986000
6	0.823208000	-2.163431000	0.815127000
1	0.870831000	-2.763382000	1.735016000
6	-0.497211000	-1.370432000	0.813469000
1	-1.341271000	-2.079023000	0.808134000
6	-0.595395000	-0.488413000	2.067068000
1	-0.535510000	-1.129182000	2.958079000
6	-1.920697000	0.275593000	2.062180000
1	-2.759793000	-0.431896000	2.072671000
1	-2.005141000	0.894734000	2.964540000
6	-1.993226000	1.158905000	0.815163000
1	-2.940953000	1.715359000	0.809725000
6	-1.913944000	0.308319000	-0.471315000
1	-2.757086000	-0.399034000	-0.485051000
6	-2.000798000	1.240135000	-1.690751000
1	-2.947150000	1.794789000	-1.653800000

1	-2.009431000	0.668098000	-2.624527000
6	-0.502701000	-1.452063000	-1.684160000
1	-1.347472000	-2.154559000	-1.645134000
1	-0.596509000	-0.903670000	-2.626419000
**2**			
6	0.078340000	-0.472619000	-0.776489000
6	0.913425000	-1.683800000	-1.235810000
1	0.408246000	-2.626262000	-1.003820000
1	1.015937000	-1.644120000	-2.329678000
6	2.302446000	-1.676527000	-0.580086000
1	2.862815000	-2.561557000	-0.903451000
6	3.050693000	-0.406582000	-0.998130000
1	3.180489000	-0.385714000	-2.087699000
1	4.051889000	-0.390321000	-0.548900000
6	2.249827000	0.815179000	-0.542920000
1	2.771733000	1.735074000	-0.842715000
6	0.849493000	0.813594000	-1.184487000
1	0.957601000	0.808279000	-2.281229000
6	0.066305000	2.067078000	-0.767219000
1	0.628651000	2.958181000	-1.079890000
6	-0.066305000	2.067078000	0.767219000
1	-0.628651000	2.958181000	1.079890000
6	1.312937000	2.062002000	1.429252000
1	1.871611000	2.964343000	1.149291000
1	1.202872000	2.072301000	2.521281000
6	2.082792000	0.814993000	0.989969000
1	3.077573000	0.809571000	1.457101000
6	1.336190000	-0.471451000	1.405011000
1	1.228715000	-0.485449000	2.500302000
6	2.153630000	-1.691028000	0.949501000
1	3.146192000	-1.654600000	1.416495000
1	1.686417000	-2.624764000	1.279799000
6	-0.078340000	-0.472619000	0.776489000
6	-0.913425000	-1.683800000	1.235810000
1	-1.015937000	-1.644119000	2.329678000
1	-0.408246000	-2.626262000	1.003820000
6	-2.302446000	-1.676527000	0.580086000
1	-2.862815000	-2.561557000	0.903451000
6	-2.153630000	-1.691028000	-0.949501000
1	-3.146192000	-1.654600000	-1.416495000
1	-1.686417000	-2.624764000	-1.279799000
6	-1.336190000	-0.471451000	-1.405011000
1	-1.228715000	-0.485449000	-2.500302000
6	-2.082792000	0.814993000	-0.989969000
1	-3.077573000	0.809571000	-1.457101000
6	-1.312937000	2.062002000	-1.429252000
1	-1.871611000	2.964343000	-1.149291000
1	-1.202872000	2.072301000	-2.521281000
6	-0.849493000	0.813594000	1.184487000

1	-0.957601000	0.808279000	2.281229000
6	-2.249827000	0.815179000	0.542920000
1	-2.771733000	1.735074000	0.842715000
6	-3.050693000	-0.406582000	0.998130000
1	-4.051889000	-0.390320000	0.548900000
1	-3.180489000	-0.385714000	2.087699000

**M1**			
1	0.163558000	4.452265000	-2.457248000
6	-0.248669000	3.156888000	0.738701000
6	0.189511000	3.114037000	-0.758688000
6	-0.600937000	2.035198000	-1.522351000
1	-0.374746000	1.036325000	-1.137293000
1	-0.281155000	2.042451000	-2.574209000
6	-2.112885000	2.289599000	-1.433236000
1	-2.648811000	1.489848000	-1.958966000
6	-2.429245000	3.643665000	-2.078185000
1	-3.507967000	3.841244000	-2.037076000
1	-2.137866000	3.635812000	-3.136200000
6	-1.665598000	4.741509000	-1.332929000
1	-1.878510000	5.718217000	-1.790227000
6	-0.147445000	4.488128000	-1.400598000
6	0.620383000	5.614684000	-0.693167000
1	0.373961000	6.570923000	-1.175751000
6	2.125474000	5.355647000	-0.787029000
1	2.436098000	5.335729000	-1.839553000
1	2.680864000	6.166577000	-0.298524000
6	2.455795000	4.020520000	-0.116154000
1	3.537163000	3.833646000	-0.175074000
6	1.715197000	2.856053000	-0.808856000
1	2.024601000	2.811433000	-1.864071000
6	2.085768000	1.539493000	-0.105616000
1	3.171893000	1.389006000	-0.163948000
1	1.626885000	0.680650000	-0.610043000
6	1.649064000	1.595818000	1.366888000
1	1.888891000	0.645686000	1.861829000
6	2.387481000	2.743764000	2.063680000
1	2.100060000	2.790768000	3.121735000
1	3.471285000	2.575652000	2.025889000
6	2.034231000	4.059784000	1.366505000
1	2.553208000	4.893189000	1.860924000
6	0.516929000	4.316131000	1.433726000
1	0.204125000	4.341558000	2.490154000
6	0.167656000	5.660519000	0.777999000
1	0.711977000	6.461631000	1.297616000
6	-1.339228000	5.908151000	0.871391000
1	-1.646210000	5.949657000	1.924359000
1	-1.593095000	6.875640000	0.419458000
6	-2.085768000	4.783908000	0.150124000
1	-3.168797000	4.960850000	0.209199000

6	-1.773863000	3.413861000	0.790413000
1	-2.086145000	3.432171000	1.845655000
6	-2.551965000	2.321579000	0.039304000
1	-3.626240000	2.538739000	0.097600000
1	-2.406062000	1.340070000	0.504710000
6	0.135506000	1.844638000	1.446847000
1	-0.180992000	1.902586000	2.498574000
1	-0.397638000	0.995408000	1.007861000
1	-0.163558000	-4.452265000	-2.457248000
6	0.248669000	-3.156888000	0.738701000
6	-0.189511000	-3.114037000	-0.758688000
6	0.600937000	-2.035198000	-1.522351000
1	0.374746000	-1.036325000	-1.137293000
1	0.281155000	-2.042451000	-2.574209000
6	2.112885000	-2.289599000	-1.433236000
1	2.648811000	-1.489848000	-1.958966000
6	2.429245000	-3.643665000	-2.078185000
1	3.507967000	-3.841244000	-2.037076000
1	2.137866000	-3.635812000	-3.136200000
6	1.665598000	-4.741509000	-1.332929000
1	1.878510000	-5.718217000	-1.790227000
6	0.147445000	-4.488128000	-1.400598000
6	-0.620383000	-5.614684000	-0.693167000
1	-0.373961000	-6.570923000	-1.175751000
6	-2.125474000	-5.355647000	-0.787029000
1	-2.436098000	-5.335729000	-1.839553000
1	-2.680864000	-6.166577000	-0.298524000
6	-2.455795000	-4.020520000	-0.116154000
1	-3.537163000	-3.833646000	-0.175074000
6	-1.715197000	-2.856053000	-0.808856000
1	-2.024601000	-2.811433000	-1.864071000
6	-2.085768000	-1.539493000	-0.105616000
1	-3.171893000	-1.389006000	-0.163948000
1	-1.626885000	-0.680650000	-0.610043000
6	-1.649064000	-1.595818000	1.366888000
1	-1.888891000	-0.645686000	1.861829000
6	-2.387481000	-2.743764000	2.063680000
1	-2.100060000	-2.790768000	3.121735000
1	-3.471285000	-2.575652000	2.025889000
6	-2.034231000	-4.059784000	1.366505000
1	-2.553208000	-4.893189000	1.860924000
6	-0.516929000	-4.316131000	1.433726000
1	-0.204125000	-4.341558000	2.490154000
6	-0.167656000	-5.660519000	0.777999000
1	-0.711977000	-6.461631000	1.297616000
6	1.339228000	-5.908151000	0.871391000
1	1.646210000	-5.949657000	1.924359000
1	1.593095000	-6.875640000	0.419458000
6	2.085768000	-4.783908000	0.150124000
1	3.168797000	-4.960850000	0.209199000

6	1.773863000	-3.413861000	0.790413000
1	2.086145000	-3.432171000	1.845655000
6	2.551965000	-2.321579000	0.039304000
1	3.626240000	-2.538739000	0.097600000
1	2.406062000	-1.340070000	0.504710000
6	-0.135506000	-1.844638000	1.446847000
1	0.180992000	-1.902586000	2.498574000
1	0.397638000	-0.995408000	1.007861000

**M2**			
1	5.596533000	-1.046733000	-0.747025000
6	2.597472000	0.237768000	0.441589000
6	4.142684000	0.226716000	0.223580000
6	4.879150000	0.136808000	1.574332000
1	4.689078000	1.020640000	2.190740000
1	5.961463000	0.115307000	1.382540000
6	4.460384000	-1.122694000	2.347731000
1	4.978273000	-1.146372000	3.313688000
6	4.830877000	-2.363610000	1.529182000
1	4.550620000	-3.274830000	2.072860000
1	5.915731000	-2.399688000	1.367190000
6	4.102540000	-2.308540000	0.184783000
1	4.365114000	-3.190086000	-0.417121000
6	4.504747000	-1.042772000	-0.596651000
6	3.813711000	-1.014729000	-1.968252000
1	4.099922000	-1.915642000	-2.529023000
6	4.238561000	0.236602000	-2.739104000
1	5.324188000	0.225167000	-2.900586000
1	3.762015000	0.253101000	-3.727687000
6	3.838166000	1.482580000	-1.946447000
1	4.134687000	2.384941000	-2.499281000
6	4.526203000	1.503347000	-0.563653000
1	5.617425000	1.506971000	-0.707473000
6	4.099636000	2.775783000	0.186916000
1	4.391394000	3.655624000	-0.400699000
1	4.615110000	2.857358000	1.149679000
6	2.576111000	2.773765000	0.391568000
1	2.277149000	3.668922000	0.949930000
6	1.889336000	2.762991000	-0.977800000
1	0.798266000	2.781793000	-0.854974000
1	2.167445000	3.658123000	-1.548707000
6	2.310240000	1.503194000	-1.737004000
1	1.814978000	1.477063000	-2.718331000
6	1.912944000	0.238745000	-0.952960000
1	0.822359000	0.244062000	-0.791503000
6	2.290246000	-1.023286000	-1.742529000
1	1.779448000	-0.998795000	-2.715794000
6	1.868381000	-2.270206000	-0.963879000
1	0.778967000	-2.272716000	-0.825933000
1	2.125353000	-3.175542000	-1.528824000

6	2.574236000	-2.286153000	0.393571000
1	2.276185000	-3.182555000	0.955718000
6	2.210198000	-1.037971000	1.226962000
1	1.121823000	-1.022299000	1.397136000
6	2.940787000	-1.108998000	2.577491000
1	2.639176000	-2.024356000	3.102576000
1	2.658342000	-0.270564000	3.222784000
6	2.164593000	1.520281000	1.177869000
1	1.072344000	1.497746000	1.307525000
1	2.594868000	1.566228000	2.182880000
1	-0.818452000	0.241782000	0.778452000
6	-4.143968000	0.229072000	-0.218648000
6	-2.599969000	0.239312000	-0.444980000
6	-2.169735000	1.523009000	-1.180799000
1	-2.604643000	1.571280000	-2.183703000
1	-1.078048000	1.499989000	-1.315429000
6	-2.576526000	2.775245000	-0.390009000
1	-2.279913000	3.671287000	-0.948196000
6	-1.882594000	2.761407000	0.975682000
1	-2.157346000	3.655457000	1.549903000
1	-0.792209000	2.780090000	0.847144000
6	-2.300114000	1.500246000	1.734563000
1	-1.799463000	1.471727000	2.713098000
6	-1.908091000	0.236998000	0.945828000
6	-2.282082000	-1.026337000	1.734927000
1	-1.765896000	-1.004233000	2.705419000
6	-1.865635000	-2.272154000	0.951532000
1	-0.777001000	-2.275634000	0.807729000
1	-2.120560000	-3.178323000	1.516075000
6	-2.578738000	-2.284753000	-0.402141000
1	-2.284497000	-3.180280000	-0.967668000
6	-2.217978000	-1.035213000	-1.234956000
1	-1.130447000	-1.020214000	-1.410772000
6	-2.955893000	-1.102909000	-2.581682000
1	-2.657898000	-2.017443000	-3.110249000
1	-2.676285000	-0.263399000	-3.226814000
6	-4.474245000	-1.115785000	-2.343741000
1	-4.997332000	-1.137053000	-3.306952000
6	-4.841500000	-2.357990000	-1.525679000
1	-5.925503000	-2.393376000	-1.357954000
1	-4.564999000	-3.268381000	-2.072655000
6	-4.105880000	-2.306252000	-0.185112000
1	-4.365938000	-3.188789000	0.416426000
6	-4.502710000	-1.041718000	0.601032000
1	-5.593674000	-1.044988000	0.757276000
6	-3.804287000	-1.016991000	1.968964000
1	-4.088226000	-1.918802000	2.529448000
6	-4.223965000	0.233125000	2.744593000
1	-5.308705000	0.222191000	2.911962000
1	-3.742028000	0.247279000	3.730595000

6	-3.826894000	1.480372000	1.952256000
1	-4.119767000	2.381851000	2.508456000
6	-4.522305000	1.504376000	0.573211000
1	-5.612740000	1.508453000	0.722889000
6	-4.098917000	2.777936000	-0.177210000
1	-4.386849000	3.656866000	0.413650000
1	-4.619539000	2.861722000	-1.137006000
6	-4.887714000	0.142545000	-1.565599000
1	-5.969014000	0.121673000	-1.368101000
1	-4.700065000	1.027419000	-2.181259000

**M3**			
1	-3.274066000	-0.338465000	-2.495027000
6	-4.336544000	0.408057000	0.726286000
6	-4.432373000	0.415631000	-0.831668000
6	-4.375279000	1.858686000	-1.370002000
1	-5.236492000	2.443525000	-1.032905000
1	-4.429283000	1.825533000	-2.467549000
6	-3.080762000	2.560751000	-0.931585000
1	-3.080493000	3.591179000	-1.305761000
6	-1.877372000	1.801649000	-1.500385000
1	-0.943247000	2.297916000	-1.201980000
1	-1.911892000	1.801909000	-2.597266000
6	-1.901914000	0.365125000	-0.974182000
1	-1.049155000	-0.197750000	-1.381787000
6	-3.200909000	-0.345904000	-1.395429000
6	-3.195241000	-1.801809000	-0.906721000
1	-2.320405000	-2.315066000	-1.330889000
6	-4.480268000	-2.502443000	-1.352136000
1	-4.542103000	-2.507068000	-2.447971000
1	-4.476577000	-3.548974000	-1.021349000
6	-5.688234000	-1.773065000	-0.760027000
1	-6.614231000	-2.276437000	-1.071444000
6	-5.739813000	-0.305668000	-1.238765000
1	-5.817083000	-0.289870000	-2.336575000
6	-6.972102000	0.376907000	-0.623467000
1	-7.876188000	-0.161044000	-0.936247000
1	-7.077076000	1.404110000	-0.988324000
6	-6.865948000	0.359597000	0.909636000
1	-7.733157000	0.870386000	1.344270000
6	-6.823988000	-1.094015000	1.392015000
1	-6.764016000	-1.127223000	2.487336000
1	-7.742522000	-1.618043000	1.098467000
6	-5.604751000	-1.786609000	0.779688000
1	-5.557630000	-2.829731000	1.123314000
6	-4.307873000	-1.071149000	1.202152000
1	-4.241136000	-1.070021000	2.302189000
6	-3.082166000	-1.798442000	0.629515000
1	-3.082142000	-2.835647000	0.993010000
6	-1.802618000	-1.088443000	1.076623000

1	-1.730596000	-1.099299000	2.172185000
1	-0.920629000	-1.615846000	0.688946000
6	-1.817744000	0.352817000	0.565141000
1	-0.896803000	0.867420000	0.874918000
6	-3.027326000	1.126704000	1.133025000
1	-2.962809000	1.134381000	2.231716000
6	-2.988805000	2.568494000	0.602527000
1	-2.047615000	3.041189000	0.912874000
1	-3.797581000	3.169263000	1.031758000
6	-5.579245000	1.075061000	1.347750000
1	-5.485372000	1.039327000	2.442528000
1	-5.641372000	2.132759000	1.074388000
1	4.241129000	-1.070025000	-2.302188000
6	4.432376000	0.415632000	0.831667000
6	4.336542000	0.408055000	-0.726287000
6	5.579241000	1.075059000	-1.347756000
1	5.641368000	2.132757000	-1.074396000
1	5.485364000	1.039324000	-2.442534000
6	6.865944000	0.359596000	-0.909645000
1	7.733152000	0.870385000	-1.344282000
6	6.823984000	-1.094017000	-1.392022000
1	7.742519000	-1.618044000	-1.098476000
1	6.764008000	-1.127226000	-2.487342000
6	5.604749000	-1.786610000	-0.779690000
1	5.557627000	-2.829732000	-1.123314000
6	4.307869000	-1.071151000	-1.202150000
6	3.082164000	-1.798444000	-0.629509000
1	3.082140000	-2.835649000	-0.993002000
6	1.802615000	-1.088446000	-1.076614000
1	1.730589000	-1.099303000	-2.172176000
1	0.920627000	-1.615848000	-0.688934000
6	1.817742000	0.352815000	-0.565135000
1	0.896800000	0.867418000	-0.874910000
6	3.027322000	1.126702000	-1.133023000
1	2.962801000	1.134377000	-2.231714000
6	2.988802000	2.568493000	-0.602527000
1	2.047611000	3.041187000	-0.912873000
1	3.797577000	3.169261000	-1.031762000
6	3.080764000	2.560753000	0.931585000
1	3.080496000	3.591180000	1.305759000
6	1.877375000	1.801650000	1.500389000
1	1.911899000	1.801912000	2.597270000
1	0.943250000	2.297917000	1.201986000
6	1.901917000	0.365126000	0.974188000
1	1.049159000	-0.197749000	1.381797000
6	3.200913000	-0.345902000	1.395432000
1	3.274073000	-0.338462000	2.495030000
6	3.195244000	-1.801808000	0.906727000
1	2.320409000	-2.315065000	1.330898000
6	4.480273000	-2.502441000	1.352138000

1	4.542111000	-2.507064000	2.447974000
1	4.476581000	-3.548972000	1.021354000
6	5.688237000	-1.773063000	0.760025000
1	6.614235000	-2.276435000	1.071440000
6	5.739817000	-0.305666000	1.238760000
1	5.817090000	-0.289866000	2.336570000
6	6.972104000	0.376909000	0.623458000
1	7.876190000	-0.161042000	0.936236000
1	7.077079000	1.404112000	0.988313000
6	4.375282000	1.858688000	1.369999000
1	4.429290000	1.825537000	2.467546000
1	5.236494000	2.443527000	1.032898000

**MP1**			
1	-5.214193000	0.482484000	2.317611000
6	-3.559945000	0.154997000	-0.718018000
6	-3.749454000	-0.048921000	0.817377000
6	-2.611348000	0.638129000	1.599676000
1	-1.638247000	0.180043000	1.384230000
1	-2.790348000	0.491836000	2.674430000
6	-2.553051000	2.139787000	1.279987000
1	-1.718540000	2.595809000	1.825973000
6	-3.872772000	2.793001000	1.703960000
1	-3.846884000	3.870748000	1.498019000
1	-4.026007000	2.669422000	2.783686000
6	-5.023074000	2.139185000	0.934780000
1	-5.977271000	2.594851000	1.235462000
6	-5.084193000	0.629059000	1.233059000
6	-6.267501000	-0.017997000	0.497518000
1	-7.197122000	0.471417000	0.820544000
6	-6.325871000	-1.512495000	0.819318000
1	-6.470845000	-1.657199000	1.897706000
1	-7.180309000	-1.976932000	0.310347000
6	-5.025098000	-2.180864000	0.369739000
1	-5.064202000	-3.256048000	0.593476000
6	-3.810076000	-1.568878000	1.099732000
1	-3.932463000	-1.715239000	2.183749000
6	-2.533355000	-2.278714000	0.623174000
1	-2.614199000	-3.351525000	0.839968000
1	-1.656205000	-1.913961000	1.169882000
6	-2.354687000	-2.069110000	-0.888626000
1	-1.426782000	-2.550569000	-1.221380000
6	-3.552313000	-2.681041000	-1.623000000
1	-3.437375000	-2.552936000	-2.706972000
1	-3.608150000	-3.759044000	-1.425180000
6	-4.834195000	-1.991494000	-1.148527000
1	-5.701572000	-2.418717000	-1.671555000
6	-4.775004000	-0.481677000	-1.445855000
1	-4.634692000	-0.334640000	-2.529103000
6	-6.079786000	0.204300000	-1.014494000

1	-6.919123000	-0.257529000	-1.553318000
6	-6.013202000	1.698614000	-1.334882000
1	-5.891042000	1.844048000	-2.416016000
1	-6.949892000	2.191094000	-1.043472000
6	-4.838047000	2.327696000	-0.584059000
1	-4.788387000	3.402747000	-0.806718000
6	-3.503123000	1.675029000	-1.002112000
1	-3.356612000	1.819916000	-2.083330000
6	-2.356591000	2.347888000	-0.230546000
1	-2.353895000	3.422264000	-0.454883000
1	-1.384333000	1.959209000	-0.551071000
6	-2.286720000	-0.566746000	-1.194518000
1	-2.169127000	-0.406047000	-2.276153000
1	-1.400864000	-0.134156000	-0.707180000
6	4.003730000	-0.417595000	-0.825686000
6	2.863786000	-0.411135000	-1.863347000
1	2.926496000	0.462747000	-2.518694000
1	2.972329000	-1.295147000	-2.507794000
6	1.490663000	-0.432048000	-1.174926000
1	0.698658000	-0.399781000	-1.933849000
6	1.360973000	-1.712591000	-0.343942000
1	1.427353000	-2.596006000	-0.992406000
1	0.380481000	-1.742944000	0.150405000
6	2.475133000	-1.742477000	0.703693000
1	2.399141000	-2.661964000	1.301059000
6	3.857891000	-1.709693000	0.025579000
1	3.944686000	-2.572951000	-0.654066000
6	4.972674000	-1.790188000	1.078506000
1	4.851379000	-2.718471000	1.654272000
6	4.831348000	-0.585757000	2.027468000
1	5.626646000	-0.629176000	2.784803000
6	3.461668000	-0.591371000	2.708967000
1	3.342336000	-1.499851000	3.313042000
1	3.379427000	0.266022000	3.389385000
6	2.365053000	-0.526146000	1.644735000
1	1.376613000	-0.536900000	2.126785000
6	2.488402000	0.762185000	0.802434000
1	2.392536000	1.635074000	1.465872000
6	1.360962000	0.781729000	-0.241201000
1	0.394766000	0.742706000	0.280653000
1	1.366340000	1.714366000	-0.815712000
6	3.881749000	0.809711000	0.130606000
6	4.105880000	2.123220000	-0.644047000
1	4.026087000	2.963126000	0.060825000
1	3.330432000	2.275747000	-1.400814000
6	5.485338000	2.136963000	-1.319450000
1	5.606187000	3.069284000	-1.883361000
6	5.606606000	0.938667000	-2.273187000
1	6.603929000	0.931867000	-2.731207000
1	4.886328000	1.028292000	-3.092930000

6	5.399444000	-0.370511000	-1.494529000
1	5.484526000	-1.226880000	-2.180813000
6	6.493626000	-0.484567000	-0.410915000
1	7.476755000	-0.467592000	-0.901844000
6	6.338756000	-1.778619000	0.389973000
1	7.139967000	-1.854697000	1.136232000
1	6.429072000	-2.647208000	-0.275132000
6	4.999105000	0.699784000	1.204706000
1	4.907456000	1.568693000	1.876481000
6	6.383617000	0.730785000	0.531071000
1	7.154391000	0.646546000	1.310275000
6	6.573551000	2.034470000	-0.246292000
1	7.567811000	2.055082000	-0.710528000
1	6.513240000	2.891307000	0.436914000

**MP2**			
1	4.867700000	0.635137000	-2.191078000
6	3.049796000	-0.036456000	0.690454000
6	3.261244000	0.086211000	-0.851198000
6	2.320945000	1.155724000	-1.440469000
1	1.270299000	0.868096000	-1.327435000
1	2.509138000	1.230793000	-2.521061000
6	2.554559000	2.520757000	-0.775099000
1	1.854936000	3.254838000	-1.193894000
6	3.996756000	2.966981000	-1.041058000
1	4.180641000	3.948569000	-0.586041000
1	4.167717000	3.069146000	-2.120271000
6	4.956415000	1.928103000	-0.455186000
1	5.995268000	2.233130000	-0.645247000
6	4.722929000	0.549664000	-1.101949000
6	5.717071000	-0.481394000	-0.546442000
1	6.739877000	-0.133854000	-0.749259000
6	5.486061000	-1.839099000	-1.212410000
1	5.646221000	-1.756589000	-2.295101000
1	6.205549000	-2.575299000	-0.831538000
6	4.058211000	-2.308797000	-0.926834000
1	3.888302000	-3.286939000	-1.398100000
6	3.025653000	-1.306098000	-1.485821000
1	3.164187000	-1.218429000	-2.574097000
6	1.611219000	-1.827493000	-1.185203000
1	1.482449000	-2.815038000	-1.647166000
1	0.850997000	-1.174379000	-1.628519000
6	1.412844000	-1.940880000	0.335083000
1	0.393700000	-2.287895000	0.550738000
6	2.428032000	-2.942039000	0.897224000
1	2.291940000	-3.049578000	1.980861000
1	2.273036000	-3.931134000	0.447846000
6	3.842390000	-2.441579000	0.594298000
1	4.581219000	-3.148733000	0.997349000
6	4.077160000	-1.063547000	1.240651000

1	3.922174000	-1.146290000	2.328604000
6	5.511860000	-0.582323000	0.976540000
1	6.215447000	-1.320608000	1.386360000
6	5.736076000	0.776901000	1.641654000
1	5.598149000	0.689415000	2.727028000
1	6.765290000	1.117544000	1.470220000
6	4.747485000	1.793200000	1.066743000
1	4.907548000	2.773390000	1.537204000
6	3.290347000	1.353680000	1.326610000
1	3.129473000	1.269958000	2.412189000
6	2.338535000	2.409214000	0.742834000
1	2.542020000	3.379107000	1.214616000
1	1.294512000	2.165464000	0.969007000
6	1.636969000	-0.570916000	0.993080000
1	1.512564000	-0.652687000	2.083144000
1	0.873166000	0.132018000	0.643861000
6	-3.049794000	0.036453000	-0.690451000
6	-1.636964000	0.570909000	-0.993073000
1	-0.873164000	-0.132027000	-0.643850000
1	-1.512554000	0.652678000	-2.083136000
6	-1.412837000	1.940873000	-0.335078000
1	-0.393691000	2.287885000	-0.550729000
6	-2.428020000	2.942034000	-0.897223000
1	-2.291923000	3.049571000	-1.980860000
1	-2.273023000	3.931129000	-0.447846000
6	-3.842380000	2.441579000	-0.594303000
1	-4.581206000	3.148734000	-0.997357000
6	-4.077152000	1.063547000	-1.240654000
1	-3.922161000	1.146288000	-2.328606000
6	-5.511855000	0.582328000	-0.976549000
1	-6.215438000	1.320615000	-1.386372000
6	-5.717071000	0.481401000	0.546433000
1	-6.739880000	0.133865000	0.749247000
6	-5.486060000	1.839106000	1.212401000
1	-6.205544000	2.575308000	0.831524000
1	-5.646224000	1.756599000	2.295091000
6	-4.058207000	2.308800000	0.926830000
1	-3.888297000	3.286942000	1.398094000
6	-3.025655000	1.306098000	1.485822000
1	-3.164193000	1.218431000	2.574097000
6	-1.611218000	1.827488000	1.185208000
1	-1.482447000	2.815033000	1.647170000
1	-0.851000000	1.174372000	1.628528000
6	-3.261247000	-0.086211000	0.851200000
6	-2.320954000	-1.155726000	1.440476000
1	-2.509151000	-1.230792000	2.521067000
1	-1.270307000	-0.868102000	1.327445000
6	-2.554571000	-2.520760000	0.775107000
1	-1.854952000	-3.254842000	1.193906000
6	-2.338540000	-2.409219000	-0.742825000

1	-2.542026000	-3.379112000	-1.214607000
1	-1.294515000	-2.165473000	-0.968994000
6	-3.290346000	-1.353683000	-1.326607000
1	-3.129467000	-1.269963000	-2.412185000
6	-4.747487000	-1.793198000	-1.066745000
1	-4.907551000	-2.773388000	-1.537205000
6	-5.736072000	-0.776896000	-1.641661000
1	-6.765288000	-1.117536000	-1.470231000
1	-5.598141000	-0.689412000	-2.727035000
6	-4.722935000	-0.549659000	1.101945000
1	-4.867710000	-0.635130000	2.191074000
6	-4.956423000	-1.928098000	0.455184000
1	-5.995278000	-2.233121000	0.645241000
6	-3.996770000	-2.966978000	1.041061000
1	-4.180656000	-3.948567000	0.586045000
1	-4.167736000	-3.069141000	2.120273000

**MP3**			
1	-5.279144000	1.280118000	-1.491947000
6	-2.869189000	-0.072016000	0.612908000
6	-4.324459000	0.285345000	0.174361000
6	-4.977920000	1.239957000	1.192735000
1	-5.088439000	0.762695000	2.171151000
1	-5.992691000	1.481124000	0.845322000
6	-4.157382000	2.529503000	1.344881000
1	-4.629997000	3.176209000	2.093391000
6	-4.099561000	3.253275000	-0.004304000
1	-3.527194000	4.185013000	0.088898000
1	-5.111574000	3.522235000	-0.332837000
6	-3.442218000	2.332868000	-1.034684000
1	-3.400617000	2.836322000	-2.010984000
6	-4.249749000	1.029711000	-1.188197000
6	-3.619635000	0.128855000	-2.261029000
1	-3.593184000	0.675725000	-3.214058000
6	-4.444559000	-1.149998000	-2.416267000
1	-5.465170000	-0.899930000	-2.733428000
1	-4.009101000	-1.790096000	-3.194290000
6	-4.477549000	-1.899348000	-1.082501000
1	-5.063524000	-2.822843000	-1.190188000
6	-5.120501000	-1.033370000	0.022515000
1	-6.151192000	-0.783806000	-0.272164000
6	-5.137249000	-1.834270000	1.334772000
1	-5.710138000	-2.758444000	1.185493000
1	-5.643221000	-1.277448000	2.130548000
6	-3.699155000	-2.180039000	1.752982000
1	-3.713787000	-2.727155000	2.702866000
6	-3.054069000	-3.045038000	0.665191000
1	-2.027391000	-3.308701000	0.952073000
1	-3.611949000	-3.982293000	0.543823000
6	-3.042767000	-2.263762000	-0.650405000

1	-2.577417000	-2.870923000	-1.439836000
6	-2.237374000	-0.961006000	-0.494883000
1	-1.210112000	-1.213410000	-0.184305000
6	-2.179168000	-0.202155000	-1.828697000
1	-1.713377000	-0.849852000	-2.585248000
6	-1.358327000	1.077138000	-1.662253000
1	-0.333832000	0.822143000	-1.361754000
1	-1.293079000	1.615851000	-2.616709000
6	-2.007771000	1.967397000	-0.601609000
1	-1.421458000	2.890121000	-0.483408000
6	-2.073208000	1.247002000	0.762911000
1	-1.049617000	1.001980000	1.087930000
6	-2.727614000	2.184463000	1.790540000
1	-2.133720000	3.104166000	1.868037000
1	-2.735686000	1.732035000	2.787731000
6	-2.884593000	-0.889022000	1.920015000
1	-1.848657000	-1.133952000	2.195236000
1	-3.296271000	-0.304399000	2.748280000
6	2.869180000	0.072024000	-0.612899000
6	2.884571000	0.889038000	-1.920001000
1	3.296235000	0.304417000	-2.748275000
1	1.848633000	1.133976000	-2.195207000
6	3.699143000	2.180049000	-1.752971000
1	3.713764000	2.727172000	-2.702851000
6	3.054077000	3.045045000	-0.665165000
1	2.027396000	3.308716000	-0.952031000
1	3.611965000	3.982296000	-0.543799000
6	3.042790000	2.263761000	0.650426000
1	2.577455000	2.870919000	1.439867000
6	2.237387000	0.961010000	0.494907000
1	1.210121000	1.213424000	0.184347000
6	2.179195000	0.202152000	1.828717000
1	1.713419000	0.849846000	2.585280000
6	3.619666000	-0.128870000	2.261026000
1	3.593226000	-0.675746000	3.214053000
6	4.444600000	1.149977000	2.416261000
1	4.009158000	1.790072000	3.194294000
1	5.465214000	0.899901000	2.733405000
6	4.477576000	1.899335000	1.082499000
1	5.063558000	2.822826000	1.190184000
6	5.120507000	1.033360000	-0.022531000
1	6.151200000	0.783789000	0.272131000
6	5.137241000	1.834269000	-1.334783000
1	5.710137000	2.758438000	-1.185506000
1	5.643198000	1.277449000	-2.130570000
6	4.324454000	-0.285348000	-0.174374000
6	4.977895000	-1.239958000	-1.192764000
1	5.992670000	-1.481133000	-0.845367000
1	5.088403000	-0.762690000	-2.171178000
6	4.157347000	-2.529499000	-1.344906000

1	4.629948000	-3.176202000	-2.093427000
6	2.727575000	-2.184447000	-1.790543000
1	2.133674000	-3.104146000	-1.868037000
1	2.735636000	-1.732013000	-2.787731000
6	2.073190000	-1.246989000	-0.762899000
1	1.049596000	-1.001959000	-1.087904000
6	2.007766000	-1.967391000	0.601618000
1	1.421446000	-2.890111000	0.483419000
6	1.358343000	-1.077135000	1.662277000
1	1.293105000	-1.615853000	2.616730000
1	0.333845000	-0.822131000	1.361795000
6	4.249760000	-1.029723000	1.188179000
1	5.279157000	-1.280138000	1.491913000
6	3.442217000	-2.332874000	1.034670000
1	3.400627000	-2.836334000	2.010967000
6	4.099541000	-3.253278000	0.004275000
1	3.527166000	-4.185012000	-0.088925000
1	5.111556000	-3.522247000	0.332792000

**MP4**			
1	5.704024000	-0.196014000	-0.994522000
6	2.585665000	0.046203000	0.513743000
6	4.120650000	0.299657000	0.393357000
6	4.870581000	-0.351132000	1.572054000
1	4.584810000	0.104271000	2.525070000
1	5.947156000	-0.166794000	1.446941000
6	4.602079000	-1.862644000	1.629218000
1	5.124978000	-2.293213000	2.491219000
6	5.106480000	-2.513514000	0.337260000
1	4.934868000	-3.597091000	0.365829000
1	6.187422000	-2.355987000	0.231476000
6	4.368578000	-1.895337000	-0.852032000
1	4.725887000	-2.348137000	-1.787800000
6	4.619784000	-0.376387000	-0.914224000
6	3.921745000	0.234973000	-2.138496000
1	4.304679000	-0.252466000	-3.046150000
6	4.198303000	1.738380000	-2.197427000
1	5.277365000	1.915995000	-2.291410000
1	3.717352000	2.178150000	-3.080605000
6	3.664061000	2.403606000	-0.927479000
1	3.853251000	3.485435000	-0.967842000
6	4.352217000	1.829164000	0.330452000
1	5.435147000	2.013211000	0.261073000
6	3.786537000	2.534919000	1.574178000
1	3.972696000	3.613403000	1.491853000
1	4.294442000	2.197913000	2.483945000
6	2.274157000	2.278500000	1.676819000
1	1.878139000	2.760620000	2.578571000
6	1.586198000	2.855360000	0.435449000
1	0.500677000	2.700259000	0.495562000

1	1.757058000	3.937844000	0.375645000
6	2.145063000	2.164750000	-0.809765000
1	1.650596000	2.560852000	-1.708500000
6	1.897412000	0.646313000	-0.743212000
1	0.813879000	0.465186000	-0.650470000
6	2.410254000	-0.037035000	-2.019194000
1	1.894240000	0.398103000	-2.887020000
6	2.137735000	-1.540797000	-1.951285000
1	1.056921000	-1.722921000	-1.884178000
1	2.492322000	-2.032234000	-2.866482000
6	2.849241000	-2.133038000	-0.733346000
1	2.658639000	-3.214440000	-0.682164000
6	2.350308000	-1.482011000	0.575054000
1	1.268134000	-1.661336000	0.677610000
6	3.092147000	-2.116520000	1.762593000
1	2.899392000	-3.196921000	1.772360000
1	2.720354000	-1.723557000	2.714759000
6	2.013289000	0.766682000	1.749571000
1	0.931668000	0.572178000	1.798263000
1	2.441208000	0.369864000	2.675180000
6	-4.119352000	-0.298678000	-0.396245000
6	-4.864585000	0.353522000	-1.577136000
1	-4.575959000	-0.101577000	-2.529431000
1	-5.941753000	0.170102000	-1.455845000
6	-4.594508000	1.864816000	-1.632456000
1	-5.114033000	2.296395000	-2.495993000
6	-5.102760000	2.515384000	-0.341847000
1	-6.184210000	2.358817000	-0.239906000
1	-4.930030000	3.598816000	-0.369166000
6	-4.369595000	1.895782000	0.849635000
1	-4.729753000	2.348323000	1.784435000
6	-4.622451000	0.377035000	0.909985000
1	-5.707136000	0.197635000	0.986363000
6	-3.929289000	-0.235700000	2.136311000
1	-4.314965000	0.251535000	3.042914000
6	-2.417133000	0.034971000	2.022486000
1	-1.904621000	-0.401186000	2.891863000
6	-2.142783000	1.538468000	1.956344000
1	-2.499802000	2.029822000	2.870637000
1	-1.061505000	1.719331000	1.892847000
6	-2.849613000	2.132154000	0.736373000
1	-2.657838000	3.213411000	0.686523000
6	-2.346790000	1.481482000	-0.570708000
1	-1.264134000	1.659895000	-0.669472000
6	-3.083899000	2.117385000	-1.760435000
1	-2.890107000	3.197613000	-1.768855000
1	-2.709135000	1.724666000	-2.711536000
6	-2.583720000	-0.046550000	-0.511103000
6	-2.007849000	-0.766741000	-1.745453000
1	-0.926003000	-0.572871000	-1.790665000

1	-2.432577000	-0.369155000	-2.672205000
6	-2.270031000	-2.278401000	-1.674401000
1	-1.871227000	-2.760333000	-2.575034000
6	-3.782997000	-2.533587000	-1.577240000
1	-3.970405000	-3.611949000	-1.496165000
1	-4.287369000	-2.195633000	-2.488623000
6	-4.352506000	-1.828024000	-0.335134000
1	-5.435839000	-2.011154000	-0.269741000
6	-3.669332000	-2.403835000	0.924864000
1	-3.859626000	-3.485518000	0.963920000
6	-4.207423000	-1.738891000	2.193339000
1	-3.729970000	-2.179651000	3.077921000
1	-5.286970000	-1.915568000	2.283430000
6	-1.900403000	-0.648064000	0.747919000
1	-0.816336000	-0.467886000	0.659232000
6	-2.149707000	-2.166286000	0.812683000
1	-1.658832000	-2.563391000	1.712933000
6	-1.586956000	-2.856572000	-0.430932000
1	-1.758892000	-3.938958000	-0.372449000
1	-0.501096000	-2.702287000	-0.486948000

**MP5**			
1	-4.671557000	-0.804398000	2.344486000
6	-3.963238000	0.494893000	-0.797694000
6	-4.207375000	0.572150000	0.742104000
6	-5.442927000	1.440750000	1.049599000
1	-5.288277000	2.477488000	0.735406000
1	-5.594215000	1.462308000	2.138274000
6	-6.695721000	0.881241000	0.358598000
1	-7.551411000	1.532193000	0.572868000
6	-6.973349000	-0.531203000	0.883627000
1	-7.873873000	-0.942250000	0.409775000
1	-7.156317000	-0.503341000	1.965368000
6	-5.766320000	-1.421109000	0.580077000
1	-5.948417000	-2.436998000	0.958515000
6	-4.500249000	-0.864487000	1.257502000
6	-3.301222000	-1.786377000	0.992770000
1	-3.532188000	-2.789530000	1.377995000
6	-2.057402000	-1.234895000	1.690472000
1	-2.224672000	-1.189784000	2.774300000
1	-1.202709000	-1.902227000	1.519788000
6	-1.748448000	0.162425000	1.148462000
1	-0.850075000	0.561996000	1.643041000
6	-2.925142000	1.129290000	1.408174000
1	-3.099578000	1.197803000	2.492616000
6	-2.562757000	2.516526000	0.853761000
1	-1.650551000	2.875373000	1.348121000
1	-3.347842000	3.247101000	1.075203000
6	-2.324539000	2.426529000	-0.662057000
1	-2.093247000	3.422185000	-1.058078000

6	-1.150059000	1.478120000	-0.923819000
1	-0.942285000	1.413258000	-2.000400000
1	-0.242562000	1.864543000	-0.438623000
6	-1.496540000	0.093593000	-0.371343000
1	-0.668137000	-0.603458000	-0.563589000
6	-2.763706000	-0.459775000	-1.049953000
1	-2.594688000	-0.509947000	-2.137790000
6	-3.078248000	-1.869838000	-0.528882000
1	-2.217271000	-2.523469000	-0.730870000
6	-4.325428000	-2.415641000	-1.226564000
1	-4.151153000	-2.480060000	-2.308281000
1	-4.545231000	-3.430384000	-0.870637000
6	-5.512496000	-1.493787000	-0.939152000
1	-6.413139000	-1.884731000	-1.432896000
6	-5.243333000	-0.066110000	-1.463034000
1	-5.078426000	-0.109113000	-2.550386000
6	-6.465313000	0.815491000	-1.159442000
1	-7.349610000	0.386520000	-1.647934000
1	-6.337590000	1.822080000	-1.571389000
6	-3.581807000	1.880156000	-1.355027000
1	-3.402312000	1.785724000	-2.435508000
1	-4.400710000	2.596043000	-1.236305000
6	3.412334000	0.081130000	0.715946000
6	2.383921000	1.163549000	1.095758000
1	1.378121000	0.884479000	0.764564000
1	2.342185000	1.241483000	2.191893000
6	2.764194000	2.522103000	0.488318000
1	2.002221000	3.265671000	0.753198000
6	4.128382000	2.953871000	1.037807000
1	4.079881000	3.061865000	2.128833000
1	4.411869000	3.930195000	0.624446000
6	5.174116000	1.900593000	0.662298000
1	6.156935000	2.195862000	1.056352000
6	4.797908000	0.529617000	1.255090000
1	4.720219000	0.621674000	2.350549000
6	5.870644000	-0.517012000	0.917455000
1	6.835790000	-0.179481000	1.320790000
6	5.975652000	-0.627416000	-0.615173000
1	6.738625000	-1.376259000	-0.870542000
6	6.346137000	0.724544000	-1.228221000
1	7.323830000	1.055032000	-0.854517000
1	6.428869000	0.630391000	-2.318645000
6	5.274687000	1.756482000	-0.869852000
1	5.539168000	2.731170000	-1.303257000
6	3.894840000	1.331873000	-1.416756000
1	3.955634000	1.243060000	-2.512126000
6	2.857023000	2.401445000	-1.041439000
1	3.161936000	3.365612000	-1.468177000
1	1.876472000	2.166065000	-1.469339000
6	3.513393000	-0.051579000	-0.836476000

6	2.187292000	-0.574788000	-1.420913000
1	2.299089000	-0.674707000	-2.510085000
1	1.372461000	0.137502000	-1.255260000
6	1.814594000	-1.933441000	-0.807839000
1	0.853953000	-2.270009000	-1.218984000
6	1.703892000	-1.802954000	0.719690000
1	1.460878000	-2.780287000	1.157403000
1	0.885933000	-1.126006000	0.990183000
6	3.035718000	-1.302658000	1.297680000
1	2.952223000	-1.207501000	2.390992000
6	4.145903000	-2.322067000	0.964597000
1	3.871208000	-3.294169000	1.397474000
6	5.492320000	-1.867012000	1.530367000
1	6.264480000	-2.614328000	1.306808000
1	5.431520000	-1.777605000	2.622631000
6	4.618281000	-1.094422000	-1.161300000
1	4.685655000	-1.184399000	-2.257590000
6	4.239356000	-2.464838000	-0.567959000
1	5.034946000	-3.183916000	-0.809536000
6	2.908143000	-2.953057000	-1.144509000
1	2.654188000	-3.935055000	-0.725420000
1	2.991072000	-3.072701000	-2.232192000

**MP6**			
1	-3.146883000	0.994767000	2.334906000
6	-4.002870000	-0.689077000	-0.579448000
6	-4.160142000	-0.290024000	0.921075000
6	-3.987505000	-1.522702000	1.830408000
1	-4.773896000	-2.262155000	1.651055000
1	-4.090938000	-1.202754000	2.877086000
6	-2.614029000	-2.178756000	1.617587000
1	-2.531341000	-3.066127000	2.255876000
6	-1.514425000	-1.174037000	1.978424000
1	-0.522219000	-1.626967000	1.846808000
1	-1.600762000	-0.880320000	3.032371000
6	-1.654042000	0.055217000	1.079617000
1	-0.876666000	0.791363000	1.330474000
6	-3.030905000	0.716562000	1.274899000
6	-3.144080000	1.982175000	0.412739000
1	-2.340390000	2.677309000	0.695239000
6	-4.507029000	2.640640000	0.633974000
1	-4.614623000	2.931235000	1.686915000
1	-4.588837000	3.555533000	0.033204000
6	-5.614020000	1.659393000	0.242974000
1	-6.595944000	2.129427000	0.393565000
6	-5.545689000	0.376209000	1.099927000
1	-5.666104000	0.646971000	2.159928000
6	-6.681653000	-0.568142000	0.674741000
1	-7.644664000	-0.061795000	0.818895000
1	-6.703052000	-1.466004000	1.301431000

6	-6.515488000	-0.949919000	-0.804637000
1	-7.312122000	-1.643906000	-1.097182000
6	-6.590752000	0.318051000	-1.661290000
1	-6.489721000	0.062870000	-2.723865000
1	-7.565570000	0.805598000	-1.533027000
6	-5.468304000	1.269805000	-1.241954000
1	-5.506243000	2.182388000	-1.853592000
6	-4.092740000	0.604049000	-1.436846000
1	-3.980164000	0.315636000	-2.494522000
6	-2.966523000	1.580287000	-1.063363000
1	-3.049165000	2.476817000	-1.693589000
6	-1.605301000	0.914500000	-1.279019000
1	-1.477451000	0.650825000	-2.337128000
1	-0.798072000	1.611967000	-1.014682000
6	-1.511606000	-0.336784000	-0.405336000
1	-0.538632000	-0.826092000	-0.561174000
6	-2.617403000	-1.353135000	-0.758942000
1	-2.507887000	-1.648791000	-1.813435000
6	-2.459440000	-2.587216000	0.143959000
1	-1.464322000	-3.023798000	-0.015056000
1	-3.187381000	-3.362796000	-0.116645000
6	-5.150159000	-1.621206000	-1.015247000
1	-5.015195000	-1.869751000	-2.077601000
1	-5.122312000	-2.568030000	-0.467305000
6	3.265961000	-0.028760000	0.579200000
6	1.909355000	0.628674000	0.892271000
1	1.095373000	0.115727000	0.372868000
1	1.705616000	0.526660000	1.968840000
6	1.908224000	2.112143000	0.494858000
1	0.921007000	2.545043000	0.705280000
6	2.983648000	2.849354000	1.300106000
1	2.767974000	2.777193000	2.373688000
1	2.991158000	3.915057000	1.038209000
6	4.347948000	2.224212000	0.997217000
1	5.128922000	2.739840000	1.573963000
6	4.358061000	0.732182000	1.380260000
1	4.119374000	0.635006000	2.451750000
6	5.741575000	0.118076000	1.120075000
1	6.490959000	0.666776000	1.707817000
6	6.065961000	0.269713000	-0.377802000
1	7.053281000	-0.169581000	-0.578553000
6	6.058946000	1.744728000	-0.783771000
1	6.825565000	2.295715000	-0.223889000
1	6.302226000	1.840117000	-1.849815000
6	4.678487000	2.342151000	-0.504362000
1	4.669366000	3.403526000	-0.789325000
6	3.585357000	1.606481000	-1.309171000
1	3.806381000	1.701343000	-2.383194000
6	2.223033000	2.248388000	-1.003430000
1	2.254018000	3.310618000	-1.278020000

1	1.430941000	1.791839000	-1.606501000
6	3.595583000	0.103144000	-0.940758000
6	2.588738000	-0.704207000	-1.783943000
1	2.858445000	-0.601501000	-2.844814000
1	1.573125000	-0.306695000	-1.681342000
6	2.600090000	-2.187287000	-1.383813000
1	1.856825000	-2.731163000	-1.978995000
6	2.264300000	-2.325096000	0.109488000
1	2.303132000	-3.382834000	0.400275000
1	1.240748000	-1.985287000	0.303806000
6	3.278979000	-1.531887000	0.948317000
1	3.031834000	-1.628443000	2.016647000
6	4.690085000	-2.108812000	0.703073000
1	4.689233000	-3.170934000	0.985331000
6	5.739417000	-1.357308000	1.524851000
1	6.730847000	-1.797669000	1.357763000
1	5.517475000	-1.450699000	2.595695000
6	5.007298000	-0.498949000	-1.182896000
1	5.235518000	-0.402739000	-2.256754000
6	5.016445000	-1.990754000	-0.799766000
1	6.024395000	-2.392308000	-0.976471000
6	3.996173000	-2.766460000	-1.636436000
1	4.019811000	-3.830460000	-1.368503000
1	4.249662000	-2.691276000	-2.701567000
