# Supplementary material for: Assigning the absolute configuration of single aliphatic molecules by visual inspection
Source: Nat Commun. 2018 Jun 20;9:2420. doi: 10.1038/s41467-018-04843-z (PMC6010418; doi:10.1038/s41467-018-04843-z)
Supplement: Supplementary file 5 — Supplementary Data 3 [file 41467_2018_4843_MOESM5_ESM.pdf]

Supplementary Data 3

Assigning the absolute configuration of single aliphatic molecules by visual inspection

Daniel Ebeling,[1,‡,] Marina Šekutor,[2,‡,*] Marvin Stiefermann,[1] Jalmar Tschakert,[1] Jeremy E. P. Dahl,[3] Robert M. K. Carlson,[3] André Schirmeisen,[1,*] and Peter R. Schreiner[2,*]*

[1]Institute of Applied Physics, Justus-Liebig University, Heinrich-Buff-Ring 16, 35392 Giessen, Germany, Daniel.Ebeling@ap.physik.uni-giessen.de, Andre.Schirmeisen@ap.physik.uni-giessen.de

[2]Institute of Organic Chemistry, Justus-Liebig University, Heinrich-Buff-Ring 17, 35392 Giessen, Germany, Marina.Sekutor@org.Chemie.uni-giessen.de, prs@uni-giessen.de

[3]Stanford Institute for Materials and Energy Sciences, Stanford, CA 94305, USA

[‡]Both contributors are considered first authors.

Supplementary Data Set 3. Geometries in Cartesian coordinates in Å computed using the GFN-xTB approach. Different orientations of (*M*)-[123]tetramantane (**1**) (Olympic rings, triangle and rhombus, **1a**, **1b** and **1c**, respectively) were modelled on a Cu(111) slab consisting of 216 copper atoms at 15 K.

Orientation **1a** of (*M*)-[123]tetramantane on Cu(111) – Olympic rings			
29	0.112615560	0.156840241	-5.712189975
29	2.615541640	0.160205765	-5.744460218
29	5.168446719	0.149403059	-5.716561729
29	7.721939895	0.139001933	-5.688441103
29	10.263523704	0.129015235	-5.651664222
29	12.813471981	0.121760325	-5.615074203
29	15.362259140	0.114172994	-5.579852317
29	17.900988066	0.110373025	-5.520687900
29	1.351010352	2.358529248	-5.821727356
29	3.922594694	2.361666960	-5.850031195
29	6.468034466	2.353686597	-5.815756593
29	9.004404877	2.346326771	-5.786072995
29	11.542200150	2.339069233	-5.752331908
29	14.084175117	2.328909008	-5.718207618
29	16.633678325	2.324467160	-5.702445622
29	19.162357941	2.280053020	-5.513121388
29	0.113918253	4.556145667	-5.726778347
29	2.650375620	4.572691283	-5.828224234
29	5.201599711	4.565341635	-5.796628251
29	7.738511922	4.552103190	-5.738896165
29	10.280013257	4.541495712	-5.703461673
29	12.821232420	4.537567007	-5.685189801
29	15.361240995	4.530849187	-5.673341666
29	17.927417930	4.519356504	-5.581036114
29	1.369471471	6.774206795	-5.752559488
29	3.935948335	6.772148345	-5.777045315
29	6.469081497	6.755010641	-5.711489810
29	9.014928975	6.747306689	-5.693047050
29	11.561673272	6.741251676	-5.665498334
29	14.098773418	6.735642843	-5.636754226
29	16.643933087	6.730877317	-5.618679261
29	19.185681221	6.703712225	-5.452544332
29	0.121911057	8.973848794	-5.658408023
29	2.669077189	8.976057846	-5.767527637
29	5.210604451	8.963798454	-5.723318041
29	7.749854482	8.958650460	-5.681191734
29	10.298190588	8.950721930	-5.649357571
29	12.838364046	8.941278066	-5.617404044
29	15.373125323	8.932591675	-5.606498193
29	17.938433654	8.920733251	-5.521086070
29	1.386330479	11.176967728	-5.701279294
29	3.950456415	11.170225959	-5.717672386
29	6.483749811	11.163189310	-5.656389263

29	9.030965119	11.156905398	-5.640635048
29	11.574214060	11.144442944	-5.596060197
29	14.108646090	11.134679169	-5.585829346
29	16.656136865	11.130639273	-5.564395885
29	19.202481675	11.118692326	-5.394758211
29	0.138817641	13.386092923	-5.611695487
29	2.680209291	13.371832894	-5.712567506
29	5.230733458	13.359603961	-5.669679130
29	7.765161083	13.356385002	-5.625039446
29	10.307706856	13.346977815	-5.597755961
29	12.843892873	13.335980338	-5.570619394
29	15.389462756	13.326458652	-5.553190713
29	17.951840280	13.320329652	-5.473648821
29	1.400748950	15.583914518	-5.651533808
29	3.965107802	15.571716742	-5.680844961
29	6.507362839	15.561516689	-5.637336156
29	9.044055387	15.550354363	-5.611567806
29	11.582868704	15.543287413	-5.577187405
29	14.122665774	15.537740319	-5.552789004
29	16.669653627	15.530612046	-5.529670296
29	19.207868060	15.534383388	-5.357008514
29	0.155624789	17.795010141	-5.498415988
29	2.682904565	17.786159281	-5.560832349
29	5.233144580	17.776568546	-5.541917270
29	7.776276336	17.767387544	-5.499873224
29	10.319319068	17.759093680	-5.467219925
29	12.864974080	17.750894786	-5.444746595
29	15.408123122	17.744182230	-5.420298078
29	17.959743670	17.730564180	-5.340841526
29	1.324566222	0.835016637	-3.690543525
29	3.894735670	0.809417195	-3.628849892
29	6.437379149	0.805808215	-3.606119458
29	8.973256620	0.799955000	-3.584106688
29	11.509192072	0.791626631	-3.548359244
29	14.048364167	0.780760625	-3.506009769
29	16.591387721	0.759812529	-3.462904459
29	19.136447619	0.799009466	-3.477981025
29	0.045934330	3.069163946	-3.692783343
29	2.622732788	3.068470197	-3.659659731
29	5.157708506	3.055445940	-3.630812269
29	7.700844123	3.048044949	-3.596027502
29	10.245983669	3.039419302	-3.565330170
29	12.792481271	3.032013714	-3.533543791
29	15.344339696	3.024879800	-3.504151471
29	17.935093307	3.026937424	-3.463152922
29	1.306968486	5.279865735	-3.647126295
29	3.889009742	5.263958163	-3.606188882
29	6.436721586	5.255453233	-3.571699492
29	8.983921125	5.248569515	-3.546998096
29	11.531449164	5.241250196	-3.515453671

29	14.072462279	5.231566749	-3.480632886
29	16.614400680	5.218063737	-3.450399686
29	19.189905785	5.222804950	-3.414214416
29	0.059565146	7.482893514	-3.628092935
29	2.632802928	7.468870086	-3.602478819
29	5.172001203	7.461747659	-3.567353172
29	7.715661571	7.454531378	-3.548177329
29	10.260732710	7.447371850	-3.517355263
29	12.808274679	7.438459351	-3.480292615
29	15.358751488	7.427488736	-3.443870663
29	17.937313872	7.424228259	-3.412176968
29	1.323660499	9.679853062	-3.589465771
29	3.900441425	9.671652331	-3.554593762
29	6.449777970	9.662979861	-3.530033341
29	8.999053516	9.655416318	-3.508294627
29	11.541544331	9.646849481	-3.468396346
29	14.086926523	9.638965863	-3.428602856
29	16.627057406	9.629894947	-3.401785194
29	19.202359152	9.621099908	-3.370735800
29	0.073456835	11.884672531	-3.583013329
29	2.646867378	11.882371051	-3.544505341
29	5.185759414	11.872906181	-3.506647092
29	7.729756696	11.864126610	-3.486031698
29	10.274354067	11.855710759	-3.456946800
29	12.822436936	11.846892794	-3.418111461
29	15.372514442	11.841230467	-3.388865378
29	17.951274392	11.827181710	-3.359106675
29	1.334651524	14.080039553	-3.530207925
29	3.916899807	14.079031140	-3.506505956
29	6.464590092	14.070767638	-3.472429426
29	9.011960173	14.062346428	-3.440159915
29	11.558858313	14.053298187	-3.412833259
29	14.099938223	14.046126921	-3.381742160
29	16.642334795	14.042188454	-3.353460488
29	19.218237489	14.019329187	-3.324440721
29	0.087439352	16.298776523	-3.516689337
29	2.664687749	16.283172671	-3.485143736
29	5.199249047	16.279895270	-3.451564681
29	7.742547762	16.270243682	-3.420718498
29	10.287623487	16.262919081	-3.388495360
29	12.834142138	16.254347601	-3.362106966
29	15.385832531	16.244784295	-3.329863592
29	17.977126361	16.225084874	-3.303772447
29	1.381012238	18.526148218	-3.484867735
29	3.952003404	18.534900675	-3.482470518
29	6.494138547	18.522323052	-3.444208108
29	9.029322891	18.511830347	-3.404380820
29	11.566009616	18.504109882	-3.378643129
29	14.105207112	18.498724076	-3.358861125
29	16.647902424	18.503247112	-3.338202736

29	19.191812224	18.445622500	-3.260518265
29	0.053183517	1.574080712	-1.704224286
29	2.578542050	1.565549361	-1.579394323
29	5.128623476	1.558131190	-1.536955275
29	7.672789153	1.551974882	-1.515545685
29	10.216212087	1.543783809	-1.487619602
29	12.762281659	1.535651716	-1.448676913
29	15.305175407	1.525623767	-1.409079951
29	17.857574284	1.522065891	-1.423618380
29	1.308609351	3.775228849	-1.521784630
29	3.872369212	3.771260116	-1.427179859
29	6.416164895	3.766230024	-1.406544215
29	8.952336665	3.761485107	-1.374705620
29	11.491571444	3.752580260	-1.347572203
29	14.031145875	3.741034114	-1.307179076
29	16.578030424	3.729900106	-1.268320088
29	19.119401370	3.708763396	-1.376361404
29	0.062376614	5.981848930	-1.593316997
29	2.600584341	5.979369195	-1.429141508
29	5.153994841	5.974961215	-1.409678419
29	7.689707590	5.967549865	-1.384368385
29	10.228472547	5.960537240	-1.355553211
29	12.766396510	5.950982633	-1.323219830
29	15.311383478	5.942017875	-1.276939257
29	17.876023470	5.931147294	-1.292966201
29	1.321445333	8.182660823	-1.474567337
29	3.886457666	8.172675862	-1.394621851
29	6.428234907	8.166930605	-1.380160538
29	8.966733965	8.159189798	-1.356519342
29	11.504894715	8.151581731	-1.323912915
29	14.043532204	8.142494238	-1.278149167
29	16.593101337	8.129564407	-1.234377742
29	19.142869696	8.123758317	-1.342384989
29	0.073395088	10.394702884	-1.549088325
29	2.617917075	10.375326988	-1.375111407
29	5.166925871	10.370093240	-1.353289110
29	7.704808187	10.358796684	-1.338854263
29	10.242582345	10.352234829	-1.318338493
29	12.781991814	10.344822134	-1.266805431
29	15.323317524	10.335743814	-1.226562953
29	17.890712871	10.330754387	-1.247118945
29	1.333121854	12.585983300	-1.422782374
29	3.899414898	12.570865062	-1.335134712
29	6.440795055	12.565360232	-1.321835780
29	8.980344780	12.557068470	-1.295832037
29	11.520591440	12.549383251	-1.264063535
29	14.059407191	12.541083139	-1.220648923
29	16.608545102	12.529798840	-1.182238230
29	19.153665497	12.539361094	-1.284119655
29	0.093734915	14.813027622	-1.479735527

29	2.626950841	14.779897417	-1.313948621
29	5.178827824	14.770193913	-1.284096423
29	7.718558155	14.762365546	-1.268346872
29	10.257202861	14.754455242	-1.241512586
29	12.797786760	14.746756231	-1.202803907
29	15.339480679	14.739496736	-1.161535240
29	17.906795287	14.733196591	-1.186752516
29	1.343482380	17.002701747	-1.352383950
29	3.913423631	16.983111550	-1.260399949
29	6.459063258	16.974853680	-1.233502003
29	8.995665831	16.966746610	-1.203316567
29	11.533972060	16.957414866	-1.175032991
29	14.075709682	16.950820771	-1.144645707
29	16.624220868	16.937614373	-1.094355160
29	19.157942888	16.965165437	-1.223449727
29	0.122783510	19.213165937	-1.492354938
29	2.624286192	19.193661913	-1.397349174
29	5.177278534	19.187859700	-1.361757232
29	7.730489573	19.182275415	-1.328084463
29	10.272408837	19.176771309	-1.304589596
29	12.823222925	19.167042416	-1.277942327
29	15.371372272	19.157228838	-1.248920580
29	17.910717801	19.144706951	-1.247906429
6	10.213080330	6.088174894	-9.599365237
1	8.784138840	9.750595688	-12.662600065
6	8.941332080	8.267433583	-9.538918882
6	8.837971407	8.275515485	-11.086239799
6	7.509226483	7.644277746	-11.542013129
1	7.450216009	6.592437384	-11.254511119
1	7.452392595	7.688081126	-12.634127025
6	6.319314565	8.399388300	-10.938764381
1	5.387842163	7.913865636	-11.252403041
6	6.337807142	9.848289742	-11.428165133
1	5.489966400	10.399287665	-11.010647152
1	6.256954202	9.877964804	-12.518499386
6	7.649537633	10.499862066	-10.987543086
1	7.676769241	11.539130314	-11.338234665
6	8.853611778	9.741943341	-11.566882172
6	10.157321332	10.431704193	-11.148756891
1	10.153611995	11.466928816	-11.511413512
6	11.353380602	9.686897582	-11.741177066
1	11.291761192	9.697552278	-12.833251906
1	12.284650550	10.181744005	-11.450253637
6	11.350760118	8.244780579	-11.233971061
1	12.212227427	7.711502402	-11.654650927
6	10.059530914	7.518841234	-11.648095326
1	9.992171670	7.502072858	-12.743319556
6	10.118762808	6.076605637	-11.129678689
1	10.999309271	5.577850342	-11.545150737
1	9.239805128	5.510522919	-11.446792771

1	10.222570132	5.059086857	-9.218880870
6	11.495079990	6.803410154	-9.186289561
1	11.573163812	6.811214331	-8.083467038
1	12.373803576	6.280207614	-9.572003791
6	11.456903435	8.238953310	-9.698858884
1	12.372203111	8.761030124	-9.391282036
6	10.242191803	8.981157200	-9.136249513
1	10.308777383	8.977276958	-8.022566461
6	10.242105479	10.432668602	-9.614352580
1	11.170171158	10.922151460	-9.292505598
6	9.045764431	11.175763204	-9.034924817
1	9.118184047	11.174123846	-7.929627885
1	9.043685673	12.219850306	-9.358433265
6	7.755270886	10.483917174	-9.453808238
1	6.899473138	11.016407759	-9.016640424
6	7.727912550	9.025950344	-8.985892237
1	7.789691254	9.010816701	-7.869356951
6	6.407185967	8.384836088	-9.408860953
1	5.575581182	8.955923760	-8.980293953
1	6.330614935	7.362158939	-9.028049318
6	9.007221230	6.826012751	-9.023604156
1	9.103275356	6.859081783	-7.918023370
1	8.094127412	6.270137514	-9.241603784

Orientation **1b** of (*M*)-[123]tetramantane on Cu(111) – triangle			
29	0.105623351	0.040121712	-5.691896135
29	2.606580061	0.051941306	-5.727006729
29	5.159859248	0.048434562	-5.701507089
29	7.713652408	0.046880446	-5.681735832
29	10.255202246	0.045290467	-5.653761651
29	12.805743774	0.047560997	-5.628559821
29	15.353424299	0.047680123	-5.597911312
29	17.892979903	0.050845778	-5.540434774
29	1.334990218	2.244781795	-5.804701605
29	3.906346781	2.257444707	-5.841296801
29	6.451958535	2.258592128	-5.814664930
29	8.988405861	2.258471761	-5.790383508
29	11.526407443	2.258920485	-5.762572316
29	14.068340187	2.257647176	-5.738113915
29	16.618045552	2.261919683	-5.728392738
29	19.148558285	2.226726391	-5.544047202
29	0.089993895	4.439570781	-5.711363480
29	2.627081624	4.464148671	-5.820680342
29	5.179058014	4.465796889	-5.799369998
29	7.716809425	4.463924836	-5.757444499
29	10.256441218	4.462452590	-5.725223867
29	12.798031766	4.464300836	-5.711923035
29	15.338700034	4.464705622	-5.704142415
29	17.905203362	4.461698647	-5.618138502

29	1.338946432	6.661791295	-5.749289769
29	3.905801343	6.667606962	-5.778593607
29	6.445453414	6.664045605	-5.741022062
29	8.981333290	6.658497802	-5.705319913
29	11.532773607	6.657735034	-5.673329256
29	14.066358247	6.665624227	-5.677869618
29	16.613469362	6.668922100	-5.661187255
29	19.156179795	6.650034755	-5.499813936
29	0.084584612	8.857427414	-5.659973189
29	2.632017893	8.867416897	-5.773570504
29	5.179238994	8.864028593	-5.744886609
29	7.708917361	8.863764539	-5.694007195
29	10.256821988	8.865384704	-5.664287876
29	12.805037219	8.861839769	-5.646881568
29	15.336394839	8.866224203	-5.651480490
29	17.901660181	8.862747668	-5.570479050
29	1.341581164	11.064696378	-5.710187430
29	3.905714447	11.066563978	-5.731473426
29	6.443499216	11.063105453	-5.690124129
29	8.982165327	11.068602338	-5.677384024
29	11.529545144	11.067776558	-5.648081052
29	14.067871046	11.064137326	-5.623828072
29	16.612476178	11.068529627	-5.617327139
29	19.159063073	11.065815052	-5.452697052
29	0.087775897	13.270921056	-5.625814240
29	2.627433400	13.263430731	-5.730354796
29	5.180323658	13.259779645	-5.696244260
29	7.714358301	13.264455968	-5.664524163
29	10.254770127	13.264995670	-5.636995924
29	12.794686356	13.263199389	-5.613465962
29	15.338535294	13.259982949	-5.608359251
29	17.900783928	13.262976797	-5.536258400
29	1.342465236	15.472249168	-5.676049310
29	3.906240387	15.468646569	-5.709680365
29	6.449861959	15.465204653	-5.676362751
29	8.985410949	15.463586978	-5.656620531
29	11.524894872	15.464557798	-5.628169407
29	14.065030006	15.467933602	-5.609180226
29	16.612165023	15.468220167	-5.592568624
29	19.150109585	15.481048645	-5.429012232
29	0.090995958	17.678915862	-5.528206408
29	2.617250060	17.678230424	-5.595248506
29	5.168071926	17.677185261	-5.582557633
29	7.711121282	17.675777743	-5.549235195
29	10.254226617	17.676092554	-5.522750050
29	12.800343081	17.676927398	-5.505523681
29	15.343342778	17.677767165	-5.488717146
29	17.895313522	17.673501804	-5.414378929
29	1.318764482	0.728881599	-3.672298085
29	3.889622872	0.711428461	-3.615677127

29	6.432764969	0.716252875	-3.599289787
29	8.968115752	0.718553541	-3.584438694
29	11.504602048	0.719214764	-3.557650710
29	14.044153791	0.716419963	-3.522215152
29	16.586459772	0.703227184	-3.485481526
29	19.132399040	0.751185430	-3.506212540
29	0.033182803	2.958762260	-3.674687820
29	2.610413896	2.965899858	-3.649211193
29	5.145955211	2.961394650	-3.629808873
29	7.688319454	2.963198617	-3.604306205
29	10.233758804	2.962312221	-3.580072952
29	12.780950701	2.963123879	-3.553264382
29	15.332702584	2.964789545	-3.531376794
29	17.923538230	2.975324890	-3.496091977
29	1.287567897	5.173572676	-3.641059409
29	3.869419959	5.165884592	-3.606170878
29	6.417802671	5.166341674	-3.584541743
29	8.964639559	5.166429168	-3.557430768
29	11.511967532	5.167611749	-3.531844862
29	14.053237741	5.167056493	-3.511857126
29	16.595934500	5.162296848	-3.487714420
29	19.171756236	5.175603997	-3.456902251
29	0.032617938	7.372750482	-3.626066262
29	2.606339146	7.366585410	-3.606008213
29	5.146551098	7.368245921	-3.580580710
29	7.688956411	7.368531609	-3.554902891
29	10.234133394	7.369918418	-3.535262795
29	12.782552818	7.369297972	-3.506566765
29	15.332725305	7.367224269	-3.484013032
29	17.911445403	7.372474483	-3.457132141
29	1.289959474	9.573595247	-3.596870047
29	3.867712276	9.573470200	-3.570353775
29	6.415605719	9.574345107	-3.544392442
29	8.965307516	9.574775039	-3.531844260
29	11.508510921	9.574818224	-3.505999471
29	14.054411772	9.574464599	-3.469422506
29	16.594139651	9.573794529	-3.449697083
29	19.169196080	9.574088425	-3.424120152
29	0.033063616	11.774759656	-3.592643842
29	2.606081805	11.780320991	-3.560220460
29	5.145701427	11.779099297	-3.532609749
29	7.688781451	11.778828025	-3.515518165
29	10.233769649	11.778720987	-3.500601992
29	12.781582695	11.778559150	-3.468527250
29	15.332548768	11.781545238	-3.436635511
29	17.911193541	11.775505618	-3.415223082
29	1.286689078	13.974279148	-3.549429501
29	3.869658693	13.981372928	-3.531887609
29	6.417429232	13.981559036	-3.507351017
29	8.963936731	13.981262018	-3.480093628

29	11.511060303	13.981209498	-3.458571060
29	14.053278698	13.981871889	-3.432494471
29	16.595089004	13.986522719	-3.412219599
29	19.170541791	13.972012791	-3.390603302
29	0.032862313	16.189002964	-3.539911682
29	2.609866336	16.181668023	-3.513489990
29	5.144515003	16.186246341	-3.488222946
29	7.687701405	16.185529796	-3.463161107
29	10.232805289	16.186006308	-3.439163406
29	12.779703959	16.185815435	-3.417494700
29	15.331493900	16.185045935	-3.392970868
29	17.922744323	16.173631716	-3.373217321
29	1.319270047	18.420575277	-3.518801312
29	3.889739020	18.437352874	-3.522033149
29	6.431223320	18.432558874	-3.490437532
29	8.967911037	18.430464920	-3.458016600
29	11.504557026	18.432277796	-3.440433657
29	14.042651256	18.434998615	-3.425895783
29	16.586847810	18.446446912	-3.410314606
29	19.130711747	18.397860945	-3.338237857
29	0.051293882	1.469540387	-1.682445279
29	2.576539190	1.469701412	-1.564634793
29	5.126812326	1.470237568	-1.529710043
29	7.671589063	1.471890607	-1.516876909
29	10.215509397	1.472481116	-1.493921178
29	12.760369153	1.473384800	-1.460972528
29	15.303462042	1.470484522	-1.432267132
29	17.856878776	1.475926483	-1.452514826
29	1.300295852	3.674488571	-1.509386460
29	3.863813132	3.680398185	-1.423310726
29	6.406701159	3.681364878	-1.415744168
29	8.944139121	3.685411735	-1.385381822
29	11.482782175	3.684069494	-1.363440394
29	14.023407460	3.681669627	-1.334186810
29	16.570920422	3.680020423	-1.302655478
29	19.112145926	3.667707916	-1.416118559
29	0.045966607	5.877372563	-1.587140257
29	2.585867927	5.883160996	-1.427914439
29	5.136938097	5.887058769	-1.415335241
29	7.674061482	5.887818002	-1.396292273
29	10.213519006	5.889467133	-1.374189112
29	12.751192487	5.888528374	-1.351014129
29	15.297238377	5.887838038	-1.312489889
29	17.861650770	5.885693159	-1.334958199
29	1.298557607	8.082561812	-1.477848086
29	3.862804691	8.081067140	-1.403482890
29	6.404899995	8.083860499	-1.394226335
29	8.944361954	8.084908170	-1.376653564
29	11.482604165	8.085709190	-1.350271017
29	14.022146348	8.084788087	-1.317689256

29	16.571292408	8.079483450	-1.278718958
29	19.120565094	8.082013091	-1.392415555
29	0.043187298	10.290210870	-1.554064736
29	2.587848937	10.279915550	-1.387786061
29	5.136511827	10.283171134	-1.370796585
29	7.675081474	10.280464220	-1.365712623
29	10.213409260	10.282596016	-1.348525637
29	12.753093519	10.283975918	-1.309064621
29	15.293519265	10.282289905	-1.272686289
29	17.861343804	10.284823932	-1.299538880
29	1.296587056	12.486002046	-1.436554980
29	3.862232546	12.479888475	-1.357467580
29	6.404015082	12.482831372	-1.351063860
29	8.944068034	12.483051836	-1.334111582
29	11.484214184	12.483276012	-1.308172884
29	14.021998777	12.482503238	-1.270070187
29	16.571515603	12.479577188	-1.234934037
29	19.116892218	12.497495503	-1.346520449
29	0.049589685	14.709290415	-1.497090176
29	2.583121086	14.684478624	-1.337225572
29	5.134721326	14.683165483	-1.317123691
29	7.674448819	14.684066968	-1.307229527
29	10.213392495	14.683311497	-1.285525608
29	12.753338891	14.683645360	-1.252731697
29	15.295715111	14.685635432	-1.218900972
29	17.862848300	14.687559006	-1.251536863
29	1.292190057	16.903255843	-1.380689649
29	3.862609654	16.892163194	-1.293543337
29	6.407854020	16.892358679	-1.276588096
29	8.944655118	16.892034501	-1.252344804
29	11.482822673	16.891643912	-1.230879620
29	14.024895391	16.893409935	-1.208284128
29	16.573373171	16.888262083	-1.163217726
29	19.106321082	16.923390570	-1.296744339
29	0.063668469	19.110279879	-1.528055157
29	2.565833199	19.097693322	-1.434871205
29	5.117769369	19.100067156	-1.404869721
29	7.671794698	19.104290441	-1.382077430
29	10.214500522	19.106317971	-1.364344900
29	12.764131062	19.105802867	-1.347119864
29	15.313143337	19.103650229	-1.321010356
29	17.852280159	19.098585726	-1.323590430
6	7.558347171	9.187450663	-12.991171339
1	12.297857441	9.436522932	-11.468290418
6	8.912683783	8.984641268	-10.879286054
6	10.141317203	9.610898171	-11.571145475
6	10.202769376	11.130187088	-11.327249152
1	9.336399062	11.637372851	-11.756110631
1	11.095768357	11.537972336	-11.811162065
6	10.257509645	11.426939121	-9.822243765

1	10.267014792	12.512151428	-9.666172091
6	11.523585558	10.809222036	-9.236979023
1	11.566848731	11.009964665	-8.150210420
1	12.416660462	11.250065189	-9.687224587
6	11.495753615	9.302003166	-9.467548974
1	12.403377827	8.850037171	-9.043264070
6	11.423791258	8.993031916	-10.973241323
6	11.442851401	7.475220342	-11.185916445
1	12.363808386	7.059928326	-10.756867851
6	11.394958488	7.173980822	-12.684999011
1	12.267842696	7.611983067	-13.178379717
1	11.420411475	6.092568918	-12.849372480
6	10.109691947	7.759729695	-13.272395127
1	10.074349187	7.537525201	-14.346575512
6	10.062717977	9.284954244	-13.078998465
1	10.920643888	9.742789480	-13.588707869
6	8.763236791	9.818516422	-13.696351668
1	8.741041934	9.562364728	-14.760262006
1	8.717292367	10.908159491	-13.622285633
1	6.632627485	9.593419707	-13.416550649
6	7.594340134	7.671066101	-13.189328468
1	6.732344987	7.205695030	-12.701769205
1	7.551732707	7.428843892	-14.255368929
6	8.890557894	7.129327655	-12.583938612
1	8.923878163	6.039662324	-12.712764568
6	8.949138262	7.454453064	-11.083042213
1	8.077978388	7.009469325	-10.582664916
6	10.226917092	6.859420417	-10.482300591
1	10.227232683	5.771971353	-10.626077027
6	10.272457897	7.171323461	-8.988619778
1	9.400895318	6.731095462	-8.483632376
1	11.177148592	6.744547794	-8.535796224
6	10.267418024	8.681729823	-8.808497080
1	10.308781321	8.903607071	-7.709372344
6	8.993686842	9.311450019	-9.369838195
1	8.119597150	8.886780398	-8.852568218
6	9.034912383	10.821339133	-9.136860814
1	9.104009278	11.007251910	-8.046301196
1	8.116072469	11.301759529	-9.479000766
6	7.600477124	9.505686385	-11.492077131
1	6.754252594	9.027050662	-10.988274647
1	7.497920467	10.583995556	-11.351174790

Orientation **1c** of (*M*)-[123]tetramantane on Cu(111) – rhombus			
29	0.035290566	0.003201823	-5.534646642
29	2.537325317	0.012693545	-5.596703500
29	5.090517422	0.008148182	-5.597501902
29	7.644182454	0.004807887	-5.600566097
29	10.186051189	0.001841009	-5.595572969
29	12.736578769	0.002095728	-5.591950367

29	15.284996964	0.001232611	-5.586395498
29	17.824536197	0.003740251	-5.555002465
29	1.266167011	2.206227204	-5.684527262
29	3.837142456	2.216082845	-5.744935701
29	6.382867024	2.215646056	-5.741932681
29	8.919416440	2.214305001	-5.741744042
29	11.457621346	2.213560135	-5.737283958
29	13.999645028	2.210745771	-5.735857129
29	16.549172372	2.213029146	-5.750245357
29	19.080569016	2.177302391	-5.589201357
29	0.023917460	4.402022014	-5.600955730
29	2.559364925	4.423760703	-5.733810391
29	5.111202173	4.423723181	-5.733699948
29	7.649478013	4.420205348	-5.713990672
29	10.188661573	4.415788396	-5.703490532
29	12.731440483	4.417048303	-5.715873320
29	15.271397613	4.416506504	-5.733246065
29	17.838492030	4.412554862	-5.670832891
29	1.273270030	6.622853944	-5.669187944
29	3.840079595	6.627200929	-5.724781327
29	6.377443984	6.621538271	-5.702706397
29	8.916640747	6.619043152	-5.703399379
29	11.465651353	6.613852260	-5.698559753
29	14.002855263	6.618477844	-5.709660061
29	16.548326890	6.620167574	-5.721920136
29	19.092160393	6.601396200	-5.584178559
29	0.021067808	8.820497139	-5.587225046
29	2.567337725	8.827543895	-5.726761675
29	5.114667083	8.822329816	-5.721089617
29	7.649947829	8.823469691	-5.701928791
29	10.192866049	8.822067488	-5.686406557
29	12.737987891	8.819483988	-5.699516875
29	15.272012152	8.818543265	-5.720818453
29	17.838278806	8.814125714	-5.664712554
29	1.279063084	11.026105789	-5.672024597
29	3.843118292	11.025608875	-5.718166970
29	6.380913041	11.022164433	-5.700048678
29	8.919351916	11.024740386	-5.684744444
29	11.464018123	11.020479006	-5.702289506
29	14.002583915	11.017713029	-5.709257829
29	16.549648088	11.020176473	-5.720616294
29	19.097773806	11.016898329	-5.579435217
29	0.027128120	13.233430601	-5.595628343
29	2.566334090	13.223698622	-5.725110397
29	5.118311747	13.218913545	-5.713475109
29	7.653849925	13.220001513	-5.710438966
29	10.194311149	13.220052879	-5.702730101
29	12.732860385	13.215803106	-5.705181253
29	15.277380512	13.212431741	-5.720703029
29	17.840346385	13.214217273	-5.671284347

29	1.282940158	15.433744100	-5.678979641
29	3.846351806	15.427684365	-5.737348722
29	6.389738205	15.423071070	-5.726797282
29	8.925623231	15.419794920	-5.729792850
29	11.465086126	15.419359609	-5.725798184
29	14.005109608	15.420879932	-5.730466032
29	16.552319401	15.420096520	-5.737509218
29	19.091941448	15.432730414	-5.596339590
29	0.034073085	17.642557598	-5.540103748
29	2.559596270	17.639433238	-5.630524430
29	5.110384792	17.636844587	-5.641025747
29	7.653608294	17.634382852	-5.630164064
29	10.196974100	17.633046213	-5.627555716
29	12.742823460	17.631820489	-5.634906378
29	15.286160777	17.631488481	-5.640898848
29	17.838683283	17.625763540	-5.591229393
29	1.269357319	0.709288923	-3.535841353
29	3.840165970	0.690867827	-3.504873001
29	6.383066322	0.693976192	-3.512511267
29	8.918646698	0.694981074	-3.521152424
29	11.455252087	0.693863614	-3.516607028
29	13.994930823	0.689889729	-3.504655741
29	16.537817880	0.675463944	-3.491908378
29	19.082727246	0.721056163	-3.536595914
29	-0.015160641	2.940078990	-3.549078689
29	2.561968397	2.946214079	-3.547309269
29	5.097191407	2.939966380	-3.550290005
29	7.640198276	2.940069785	-3.548485223
29	10.185563812	2.937680642	-3.547205884
29	12.732645478	2.937150539	-3.544565817
29	15.284582515	2.936931823	-3.546074793
29	17.875703988	2.946085609	-3.535164977
29	1.240416444	5.154379281	-3.546542067
29	3.822846698	5.145525604	-3.537489907
29	6.370898444	5.144305412	-3.536776459
29	8.918068616	5.143542687	-3.538222033
29	11.465755376	5.142912341	-3.534913078
29	14.007012887	5.140362333	-3.534797664
29	16.549446776	5.133965705	-3.534091527
29	19.125190778	5.145756279	-3.528065551
29	-0.012681326	7.354479804	-3.538399004
29	2.560869817	7.347017212	-3.544940724
29	5.100584455	7.346804178	-3.541754976
29	7.643704193	7.346018321	-3.544269373
29	10.189020231	7.345209625	-3.546972494
29	12.737035251	7.343749923	-3.543440867
29	15.287984028	7.339802051	-3.539479175
29	17.866738599	7.343638078	-3.537071797
29	1.245925810	9.554778298	-3.542466897
29	3.823682352	9.553110083	-3.540994406

29	6.372471478	9.552175929	-3.541871387
29	8.921285546	9.550264086	-3.544491660
29	11.464991635	9.549736050	-3.544437385
29	14.010766093	9.548159210	-3.536304132
29	16.550814596	9.545977165	-3.539129034
29	19.126053005	9.544090609	-3.536613724
29	-0.009762097	11.756595666	-3.548124371
29	2.563781033	11.761033621	-3.540163806
29	5.103417476	11.758407574	-3.537300092
29	7.645885203	11.756856351	-3.538618839
29	10.192253205	11.754984985	-3.544818383
29	12.739665535	11.752934102	-3.541778523
29	15.290408964	11.754318612	-3.536841217
29	17.869461728	11.746716092	-3.536942465
29	1.245896825	13.955652362	-3.537332384
29	3.828581197	13.961289808	-3.543874148
29	6.376682976	13.959862723	-3.544926777
29	8.923378978	13.958007793	-3.541921357
29	11.470931689	13.956258938	-3.543809287
29	14.012608954	13.955567652	-3.541709593
29	16.554743940	13.958665904	-3.543489915
29	19.130464141	13.942594340	-3.544265017
29	-0.006710610	16.171219006	-3.537775812
29	2.570589250	16.162348650	-3.535260494
29	5.105319401	16.165752540	-3.533055605
29	7.648637505	16.163300396	-3.531667650
29	10.193841739	16.162467081	-3.530545357
29	12.740826067	16.160617703	-3.533658732
29	15.292726641	16.158070890	-3.532105773
29	17.884032282	16.145152052	-3.536619660
29	1.281174003	18.401975458	-3.548757723
29	3.851530395	18.417074880	-3.576153776
29	6.393741841	18.411149576	-3.567906340
29	8.929983975	18.407669596	-3.558694928
29	11.466655371	18.407305026	-3.563905272
29	14.005353450	18.408251753	-3.573338806
29	16.549143388	18.418636331	-3.581813456
29	19.093368519	18.368956652	-3.533498305
29	0.020085962	1.469546038	-1.542943678
29	2.546580938	1.469061178	-1.448839242
29	5.097108893	1.468263086	-1.437712381
29	7.641441055	1.468451038	-1.448190019
29	10.185424248	1.467564799	-1.448747745
29	12.730988987	1.466779849	-1.439638063
29	15.274184741	1.462913363	-1.432789321
29	17.826967129	1.465897804	-1.477643188
29	1.272008370	3.675633312	-1.402112545
29	3.836399705	3.679774462	-1.339635180
29	6.379556096	3.680225723	-1.353148082
29	8.916704745	3.682415880	-1.349269993

29	11.455740138	3.680036103	-1.350609638
29	13.996038445	3.675879623	-1.343090860
29	16.543320779	3.672393027	-1.333898612
29	19.083293466	3.656512776	-1.472021907
29	0.018775094	5.878332651	-1.486630604
29	2.559011330	5.883939173	-1.353396757
29	5.111133159	5.885906518	-1.365139068
29	7.648024829	5.884849760	-1.368912804
29	10.187299674	5.885197583	-1.369780899
29	12.725280990	5.882757649	-1.370178681
29	15.270976544	5.880956437	-1.354112142
29	17.834850041	5.876522398	-1.400050014
29	1.273321691	8.083589969	-1.410022444
29	3.838185105	8.081094422	-1.361623898
29	6.380099612	8.082003315	-1.376039540
29	8.920049763	8.080875793	-1.380407404
29	11.458026242	8.080212876	-1.379739956
29	13.997742083	8.077984090	-1.368547316
29	16.546669104	8.071948405	-1.352168771
29	19.094466373	8.071522710	-1.489453825
29	0.018536998	10.291391184	-1.496095976
29	2.564893641	10.280912684	-1.352803049
29	5.112878107	10.282391537	-1.361781479
29	7.651765102	10.277814099	-1.376316471
29	10.190004873	10.278103844	-1.384193953
29	12.730452131	10.277880701	-1.368846061
29	15.271270258	10.275217160	-1.357288752
29	17.837955049	10.276069691	-1.407172295
29	1.274152820	12.487324256	-1.411233428
29	3.840509693	12.479995933	-1.356151771
29	6.381740529	12.481782989	-1.373207382
29	8.922410939	12.480001869	-1.377298553
29	11.462816932	12.479083308	-1.376723986
29	14.001634846	12.477007540	-1.364819754
29	16.550576201	12.472348557	-1.352176864
29	19.094228860	12.487576394	-1.485301031
29	0.027850430	14.710666961	-1.481675385
29	2.563109779	14.685547435	-1.343515531
29	5.114689182	14.682981860	-1.348133365
29	7.654410609	14.682257024	-1.362921479
29	10.193275119	14.680259152	-1.364759214
29	12.733999077	14.679324609	-1.356207057
29	15.276197122	14.679177412	-1.344177809
29	17.843049522	14.678985146	-1.400482022
29	1.272932640	16.904720218	-1.397022287
29	3.843961612	16.892634289	-1.333326210
29	6.389555479	16.890912931	-1.338136506
29	8.926492487	16.889544195	-1.338051606
29	11.464755852	16.887434273	-1.340632278
29	14.006895635	16.887531890	-1.341139948

29	16.555799341	16.881102065	-1.320208539
29	19.087523591	16.913235031	-1.478446590
29	0.044611992	19.110562900	-1.551309758
29	2.547493802	19.097908172	-1.483690019
29	5.099837326	19.098856854	-1.477726839
29	7.653899066	19.100965098	-1.476642940
29	10.196230006	19.101641654	-1.482478805
29	12.746466558	19.099170584	-1.487534310
29	15.295229408	19.095682255	-1.486472626
29	17.834480089	19.088684387	-1.512393003
6	8.656246049	11.001516916	-9.217931934
1	12.327418559	8.555762042	-11.519269741
6	9.070994208	8.797906034	-10.366494798
6	10.594604387	9.035201149	-10.314707970
6	11.213440675	8.358670032	-9.080993521
1	10.801640426	8.780387805	-8.151767453
1	12.294154467	8.535816167	-9.063653525
6	10.928766591	6.858505811	-9.094351666
1	11.352238708	6.408469034	-8.172735066
6	11.577770494	6.222157739	-10.319223507
1	11.392047633	5.144370236	-10.327694622
1	12.659597007	6.382285456	-10.299190084
6	10.970961408	6.874026121	-11.564449016
1	11.429409914	6.433013599	-12.458717161
6	11.242858221	8.385436138	-11.558753435
6	10.682829507	9.013664476	-12.839917547
1	11.162814962	8.546051519	-13.708942923
6	10.976788668	10.514430876	-12.845040219
1	12.057624091	10.681964153	-12.811678389
1	10.592281404	10.968924804	-13.762958113
6	10.309866133	11.160053886	-11.629712037
1	10.516048034	12.237982647	-11.634698236
6	10.857685494	10.555753205	-10.324312777
1	11.938573104	10.736877237	-10.262645870
6	10.162203038	11.252058761	-9.152263382
1	10.362178168	12.327469814	-9.190594701
1	10.562378763	10.886504654	-8.193506394
1	8.172740291	11.472163569	-8.342134364
6	8.102681692	11.611568628	-10.503739446
1	7.021195264	11.457319277	-10.558944080
1	8.294906040	12.688113610	-10.524358944
6	8.792763774	10.930278640	-11.688535661
1	8.399755241	11.351375072	-12.622737753
6	8.518540073	9.418736308	-11.670306639
1	7.433765314	9.249633437	-11.708241940
6	9.170614504	8.761538024	-12.891876296
1	8.755928203	9.207649741	-13.804684577
6	8.876705111	7.260625293	-12.883246814
1	7.796221603	7.092746104	-12.924422408
1	9.326516397	6.784983801	-13.759870916

6	9.453367477	6.643365867	-11.608253706
1	9.247975064	5.565238127	-11.603970350
6	8.812179980	7.276033587	-10.359465756
1	7.730180684	7.092037749	-10.371312905
6	9.423943102	6.603070550	-9.127261267
1	9.228427057	5.526821014	-9.160891243
1	8.961051086	6.984738678	-8.202436757
6	8.357284882	9.502073032	-9.200289232
1	7.277692729	9.337944569	-9.279865321
1	8.673738599	9.091503888	-8.231328563

Orientation **a** of (*P*)-[123]tetramantane on Cu(111) – Olympic rings			
29	0.135939066	0.196461347	-5.400843710
29	2.605969804	0.186810134	-5.456139965
29	5.150134919	0.181828974	-5.442700803
29	7.706102320	0.169529497	-5.430453298
29	10.242273491	0.160306687	-5.406908436
29	12.791227259	0.157150543	-5.377590383
29	15.335563210	0.152010339	-5.350569700
29	17.849938845	0.150468132	-5.301909986
29	1.320015055	2.362494349	-5.583736739
29	3.910570796	2.379063182	-5.686192750
29	6.458931892	2.370534891	-5.668483392
29	8.988039605	2.362243007	-5.652414212
29	11.517435379	2.356914784	-5.623266364
29	14.057088894	2.351272596	-5.600001633
29	16.605656313	2.352748949	-5.608313290
29	19.107860303	2.289211670	-5.328920687
29	0.127698938	4.558742313	-5.461264717
29	2.637123622	4.589273776	-5.654414118
29	5.191108258	4.585125783	-5.641425671
29	7.725189889	4.574630866	-5.589918286
29	10.256457368	4.567856740	-5.571363817
29	12.793343828	4.562738343	-5.561808739
29	15.331779243	4.556097042	-5.562917401
29	17.914403694	4.540952264	-5.406883450
29	1.340195679	6.785358152	-5.512315832
29	3.924118258	6.792302006	-5.613266622
29	6.453523832	6.769299797	-5.537404369
29	8.998526949	6.758084168	-5.516727615
29	11.533999057	6.753735663	-5.505704667
29	14.068277855	6.756190597	-5.505746980
29	16.612902685	6.758461796	-5.506892745
29	19.130508071	6.720693861	-5.261682520
29	0.133595490	8.976076467	-5.390629610
29	2.655646592	8.990149578	-5.603669180
29	5.193421351	8.976321231	-5.553219114
29	7.727904791	8.969360323	-5.528414952
29	10.271341108	8.957136316	-5.506310878
29	12.812454537	8.954262835	-5.512147369

29	15.343094295	8.953179441	-5.484528512
29	17.921054368	8.939333755	-5.353789249
29	1.357169970	11.181429264	-5.469693291
29	3.936901593	11.179733832	-5.556973555
29	6.460123410	11.173267398	-5.475016844
29	9.005401935	11.170384934	-5.492312452
29	11.545598143	11.159303489	-5.462375041
29	14.082702464	11.151334270	-5.432803482
29	16.624217680	11.147746125	-5.455640969
29	19.145421067	11.131556044	-5.207274125
29	0.149569799	13.382756060	-5.352454746
29	2.664009890	13.374192867	-5.552036951
29	5.219375307	13.361414366	-5.512095670
29	7.743096278	13.365164386	-5.472408604
29	10.278526153	13.360515921	-5.441295398
29	12.811553976	13.347125050	-5.424412928
29	15.353247323	13.333478733	-5.440897044
29	17.929244117	13.329466104	-5.316889237
29	1.370302083	15.586571563	-5.415320064
29	3.949503472	15.574274023	-5.531502881
29	6.491958101	15.564584122	-5.494870416
29	9.020439286	15.554345408	-5.487136300
29	11.550336870	15.548646612	-5.458053622
29	14.085000077	15.545618577	-5.441108084
29	16.632403270	15.539703273	-5.425079131
29	19.144759494	15.540948109	-5.179472661
29	0.166418746	17.775127206	-5.206158483
29	2.664390368	17.785812492	-5.306617682
29	5.212345039	17.776188652	-5.305172730
29	7.752527632	17.763783207	-5.268348138
29	10.288883564	17.755550631	-5.243772965
29	12.830168219	17.750982987	-5.235139541
29	15.369532908	17.748437963	-5.220981924
29	17.916990636	17.727726171	-5.130504159
29	1.321295501	0.809736701	-3.382920694
29	3.901085927	0.768357121	-3.323562033
29	6.440916612	0.768648298	-3.318538975
29	8.966862683	0.766292652	-3.312939208
29	11.492540169	0.759677349	-3.286672273
29	14.025053999	0.749506576	-3.248320630
29	16.561537893	0.713331558	-3.210433227
29	19.095892772	0.818820104	-3.274666993
29	0.025118159	3.062518407	-3.428778749
29	2.630857079	3.086919424	-3.395979913
29	5.152711738	3.064484906	-3.382051169
29	7.689184336	3.060401057	-3.362761639
29	10.229073697	3.053606164	-3.345993275
29	12.773952466	3.046954575	-3.319431706
29	15.333000615	3.040351932	-3.301660298
29	17.969579883	3.056838823	-3.259171755

29	1.256056070	5.291586858	-3.367856597
29	3.877204038	5.277377116	-3.326656342
29	6.428027752	5.268353253	-3.297436058
29	8.973550096	5.263321644	-3.276350736
29	11.517572340	5.257853724	-3.260064181
29	14.053392187	5.248512685	-3.243359762
29	16.592378305	5.233900691	-3.231501254
29	19.197147543	5.254068683	-3.192823651
29	0.028220407	7.494251134	-3.335247819
29	2.628311145	7.476563522	-3.327012915
29	5.161983167	7.471434468	-3.294636149
29	7.701574783	7.465953637	-3.286538977
29	10.241696715	7.460381654	-3.263186095
29	12.789602319	7.453723474	-3.245868362
29	15.345519495	7.439799836	-3.220860439
29	17.956128615	7.442750717	-3.199193317
29	1.274374167	9.685799858	-3.308757247
29	3.883600894	9.679104277	-3.275966260
29	6.438129483	9.671064156	-3.266952952
29	8.987746210	9.665962043	-3.276721705
29	11.525122794	9.658688654	-3.250634115
29	14.065848190	9.651042500	-3.201571557
29	16.600904205	9.644443781	-3.179488887
29	19.205252894	9.636663990	-3.163004922
29	0.040239753	11.883618596	-3.306020762
29	2.639987698	11.887353287	-3.268871795
29	5.172816171	11.879159662	-3.230017553
29	7.713332482	11.871212867	-3.231520163
29	10.252757602	11.863985406	-3.224342630
29	12.801094508	11.856168918	-3.187241852
29	15.357595644	11.856218003	-3.152324951
29	17.968125701	11.838071444	-3.145625349
29	1.279045146	14.080154539	-3.249853363
29	3.900541379	14.079872654	-3.243708881
29	6.450475326	14.074153156	-3.213561907
29	8.996662367	14.066619288	-3.186133213
29	11.541396747	14.058749132	-3.167582721
29	14.077675260	14.053825299	-3.147092815
29	16.616094269	14.055559719	-3.137749076
29	19.220750087	14.019365340	-3.131243162
29	0.060426577	16.316077482	-3.207260716
29	2.665560910	16.277115147	-3.199133530
29	5.187092805	16.286103790	-3.170992695
29	7.723673931	16.275626680	-3.147986311
29	10.263500829	16.269511931	-3.124258820
29	12.808629162	16.262844698	-3.110650371
29	15.367646840	16.255842075	-3.087041473
29	18.004487184	16.225071505	-3.084431458
29	1.369235983	18.563126012	-3.227480098
29	3.949594430	18.590252243	-3.246084190

29	6.489044953	18.576641907	-3.212154970
29	9.014310489	18.565347812	-3.177171508
29	11.540601879	18.559014452	-3.162834622
29	14.073054504	18.555665132	-3.159620572
29	16.609719586	18.578770495	-3.157047467
29	19.142073281	18.457873846	-3.051961252
29	0.088592992	1.604972759	-1.424399308
29	2.585491543	1.578566360	-1.283490078
29	5.133318486	1.573632198	-1.245604993
29	7.674527658	1.572949290	-1.241768362
29	10.211595615	1.567512066	-1.226032009
29	12.752825629	1.558974517	-1.192996373
29	15.292074773	1.547769108	-1.165001748
29	17.840047991	1.554907119	-1.213113949
29	1.300451277	3.783942725	-1.200323069
29	3.877953877	3.781770902	-1.044863209
29	6.422887864	3.778791209	-1.037114597
29	8.951376852	3.777433458	-1.005870512
29	11.481066519	3.768832930	-0.996287517
29	14.016775681	3.757470482	-0.971094062
29	16.563879368	3.748273716	-0.941470425
29	19.079804340	3.733442399	-1.144586666
29	0.092444439	5.994690707	-1.285934125
29	2.603213758	5.989657495	-1.045309034
29	5.162891170	5.990462545	-1.040512160
29	7.691809842	5.985996604	-1.019018057
29	10.219218117	5.982419568	-1.005977362
29	12.750931929	5.972082017	-0.989107633
29	15.295786154	5.962935294	-0.949092075
29	17.874846949	5.951870559	-1.029919559
29	1.310140807	8.190170471	-1.147344673
29	3.892330819	8.178752694	-1.017030909
29	6.433269403	8.178870233	-1.017956659
29	8.965649906	8.172908319	-1.010309969
29	11.491566778	8.167848613	-0.993031760
29	14.025554435	8.156657514	-0.954674375
29	16.578263630	8.141670455	-0.910181785
29	19.105086595	8.143466213	-1.119563913
29	0.101117538	10.402304533	-1.246145821
29	2.620278204	10.374629001	-0.989336059
29	5.173676320	10.371423219	-0.980616117
29	7.706962962	10.360089065	-0.987689345
29	10.232160021	10.357353425	-0.994121502
29	12.765733834	10.351829590	-0.936569712
29	15.302195399	10.341731384	-0.896796292
29	17.888952300	10.342347702	-0.990063861
29	1.317189540	12.586384775	-1.105241888
29	3.901156006	12.564759812	-0.960866301
29	6.442670883	12.562220462	-0.962956107
29	8.977501565	12.553436421	-0.954640157

29	11.506513713	12.548664363	-0.929892481
29	14.038889150	12.540522788	-0.887160232
29	16.590903780	12.530597444	-0.857769865
29	19.111994075	12.555071462	-1.062908864
29	0.118944806	14.820270754	-1.174144378
29	2.623943218	14.777116004	-0.943073384
29	5.180124333	14.765863907	-0.913609510
29	7.716196728	14.759300161	-0.912642989
29	10.245044369	14.751302908	-0.896817265
29	12.780576752	14.745738758	-0.861268343
29	15.321072845	14.741450171	-0.829134955
29	17.906062842	14.742031543	-0.940335386
29	1.322448842	17.010246154	-1.030253609
29	3.910473290	16.980960328	-0.889662216
29	6.459431289	16.976318514	-0.866945822
29	8.988454745	16.970977952	-0.842581883
29	11.518290176	16.962402293	-0.829203160
29	14.057912317	16.955001376	-0.811602847
29	16.605282443	16.938883192	-0.759989427
29	19.112295771	16.988107199	-0.996776362
29	0.154266299	19.183308268	-1.227987665
29	2.622936946	19.180228368	-1.133923131
29	5.166828410	19.171644429	-1.107693627
29	7.722399297	19.170732180	-1.080499241
29	10.258836166	19.167299339	-1.064984334
29	12.808002325	19.155998297	-1.051011857
29	15.352182629	19.146793438	-1.035740939
29	17.866345315	19.135976735	-1.046651573
6	9.967613566	10.654281555	-10.910381355
6	9.826874865	12.118154756	-11.367826235
1	10.706719667	12.704665220	-11.095268317
1	9.742729070	12.142231940	-12.458736251
6	8.582311356	12.760278533	-10.744752743
1	8.520033518	13.807991377	-11.061097061
6	7.334600851	12.006239085	-11.206929393
1	7.245831542	12.056591950	-12.295852728
1	6.437469870	12.457339853	-10.772825430
6	7.449839353	10.547182002	-10.763749571
1	6.561991076	9.993376571	-11.093706206
6	8.703354673	9.894576770	-11.365446655
1	8.639492558	9.947956630	-12.460202590
6	8.779274452	8.422076121	-10.946428147
1	7.878449651	7.898639862	-11.289616258
6	8.852063779	8.352063465	-9.413203236
1	8.906282297	7.304787791	-9.089037971
6	7.618325638	9.009074046	-8.811067708
1	6.709459135	8.478165567	-9.105500699
1	7.686919784	8.954821153	-7.704453719
6	7.548671408	10.470163526	-9.231508539
1	6.663383209	10.936716482	-8.777855275

6	8.799198588	11.235756346	-8.790428971
1	8.865299077	11.195570480	-7.675217378
6	8.671042129	12.697672832	-9.216286880
1	7.765771479	13.124768380	-8.770363307
1	9.520468753	13.284507614	-8.855773943
6	10.057838658	10.572495896	-9.363962228
6	11.342747831	11.246590381	-8.869007413
1	11.383199225	11.145732591	-7.765957382
1	11.354061817	12.315570215	-9.086939996
6	12.578767182	10.581792017	-9.470125302
1	13.477780423	11.096536448	-9.109913509
6	12.510030267	10.668442836	-10.999969255
1	13.379421428	10.165566811	-11.433425400
1	12.542575084	11.713464443	-11.316971932
6	11.228948381	9.985818010	-11.494920782
1	11.189098319	10.051852433	-12.589667224
6	11.267489374	8.505314746	-11.080516337
1	12.155725282	8.033529687	-11.518494407
6	10.016780588	7.768957044	-11.561217878
1	10.069463933	6.716306315	-11.268073896
1	9.954235570	7.813490820	-12.652314690
6	10.111584918	9.089611222	-8.961893341
1	10.171387567	9.033576233	-7.848887088
6	11.356475861	8.418547591	-9.546661464
1	11.375549978	7.365065358	-9.238754779
6	12.622566356	9.113850829	-9.057503711
1	13.510973217	8.622997504	-9.463088249
1	12.678582184	9.040040481	-7.955173653
